# Efficient prime editing in mouse brain, liver and heart with dual AAVs

**DOI:** 10.1038/s41587-023-01758-z

**Published:** 2023-05-04

**Authors:** Jessie R. Davis, Samagya Banskota, Jonathan M. Levy, Gregory A. Newby, Xiao Wang, Andrew V. Anzalone, Andrew T. Nelson, Peter J. Chen, Andrew D. Hennes, Meirui An, Heejin Roh, Peyton B. Randolph, Kiran Musunuru, David R. Liu

**Affiliations:** 1 Merkin Institute of Transformative Technologies in Healthcare, Broad Institute of MIT and Harvard, Cambridge, MA USA; 2 Department of Chemistry and Chemical Biology, Harvard University, Cambridge, MA USA; 3 Howard Hughes Medical Institute, Harvard University, Cambridge, MA USA; 4 Cardiovascular Institute, Perelman School of Medicine at the University of Pennsylvania, Philadelphia, PA USA; 5 Division of Cardiovascular Medicine, Department of Medicine, Perelman School of Medicine at the University of Pennsylvania, Philadelphia, PA USA; 6 Department of Genetics, Perelman School of Medicine at the University of Pennsylvania, Philadelphia, PA USA

**Keywords:** Genetic engineering, Cell delivery, Targeted gene repair

## Abstract

Realizing the promise of prime editing for the study and treatment of genetic disorders requires efficient methods for delivering prime editors (PEs) in vivo. Here we describe the identification of bottlenecks limiting adeno-associated virus (AAV)-mediated prime editing in vivo and the development of AAV-PE vectors with increased PE expression, prime editing guide RNA stability and modulation of DNA repair. The resulting dual-AAV systems, v1em and v3em PE-AAV, enable therapeutically relevant prime editing in mouse brain (up to 42% efficiency in cortex), liver (up to 46%) and heart (up to 11%). We apply these systems to install putative protective mutations in vivo for Alzheimer’s disease in astrocytes and for coronary artery disease in hepatocytes. In vivo prime editing with v3em PE-AAV caused no detectable off-target effects or significant changes in liver enzymes or histology. Optimized PE-AAV systems support the highest unenriched levels of in vivo prime editing reported to date, facilitating the study and potential treatment of diseases with a genetic component.

## Abstract (web summary)

Prime editors are efficiently delivered to mouse organs with AAV vectors.

## Main

The ability to precisely correct pathogenic mutations or install protective ones in living systems has enormous therapeutic potential^1^. Prime editors (PEs) are precision gene editing agents that can perform virtually any substitution, small deletion and small insertion at target DNA sites in living cells^2^. PEs do not rely on double-strand breaks, minimizing unwanted outcomes associated with nucleases such as uncontrolled insertions and deletions (indels)^3–5^, large deletions^6,7^, chromosomal abnormalities^8–13^ or induction of the p53 DNA damage response^14^. PEs comprise a programmable nickase fused to an engineered reverse transcriptase (RT). A prime editing guide RNA (pegRNA) contains a spacer that guides the PE to the target DNA site as well as a 3′ extension that encodes the desired edit. PEs reverse transcribe the pegRNA extension directly into the genome using the nicked target DNA strand as a primer, leading to permanent changes in genomic DNA with relatively few byproducts^2^. PEs do not require a donor DNA template and have been used widely across many mammalian cell systems, including mitotic and post-mitotic cells^3,15–21^.

Before PEs can be translated into clinical settings, safe and efficient delivery methods capable of targeting therapeutically relevant tissue types are needed. Adeno-associated viruses (AAVs) are clinically validated and FDA approved for in vivo gene therapy and gene editing applications^22,23^. Although not without potential risks^24–27^, AAV remains one of the few effective and clinically validated in vivo delivery vectors for a variety of non-liver organs and tissue types^28,29^. At ~6.3 kilobases (kb) of encoded DNA sequence, however, PEs are currently too large to fit within the ~4.7-kb cargo size limit of AAV^30,31^.

Our laboratory and others have overcome the packaging capacity of AAV to accommodate large genome editing agents by splitting them into two halves, each fused to a fast-splicing intein^32–34^. Co-transduction of both AAVs reconstitutes the editing agent via association, or association and splicing, of the split intein-fused proteins. Existing in vivo PE delivery strategies with intein-split AAV or hydrodynamic DNA injection have thus far yielded modest in vivo editing efficiencies in postnatal animals in contexts without a survival advantage of edited cells, achieving maximum efficiencies corresponding to 1.7–13.5% editing of bulk liver or retina^15–21^. A recent report^35^ demonstrates efficient in vivo prime editing in enriched degenerating retinitis pigmentosa mouse retinas with dual-AAV delivery of PE-SpRY, but this system may not be applicable to uses that do not confer a survival advantage among edited cells. Adenoviral transduction has yielded the most efficient generalizable in vivo prime editing reported to date^16^ (up to 58% editing in isolated hepatocytes, corresponding to ~35–40% editing of bulk liver^36^) in neonatal animals at the highest dose but may be challenging to apply clinically due to the immunogenicity and toxicity of adenovirus^37^. The efficient in vivo delivery of PEs into therapeutically relevant cells using AAV, therefore, remains a major bottleneck to the use of prime editing for animal research and therapeutic applications.

Here we report the development, optimization and application of intein-split PE-AAVs that mediate efficient in vivo prime editing in multiple mouse organs. We systematically optimized the PE protein, pegRNA and AAV genomic elements, resulting in optimized ‘v3em PE-AAV’ delivery strategies that enabled therapeutically relevant levels of prime editing in mouse brain (42%), liver (46%) and heart (11%), representing, to our knowledge, the first prime editing in postnatal brain and heart and substantially higher AAV-mediated in vivo prime editing efficiencies than have been previously reported in the liver. We applied v3em PE3-AAV to install mutations of biomedical interest in mice that are not currently accessible with other in vivo genome editing technologies, including the rare Apolipoprotein E Christchurch (*APOE3* R136S) variant that may alter Alzheimer’s disease (AD) risk and the dominant variant of proprotein convertase subtilisin/kexin type 9 (*PCSK9)* Q152H (mouse *Pcsk9* Q155H) that is associated with a reduction in low-density lipoprotein (LDL) cholesterol levels and protection from coronary artery disease. Our results advance the potential of prime editing for basic research and therapeutic applications and establish optimized PE-AAV systems as an effective in vivo PE delivery method.

## Results

### Design and evaluation of split PE architectures

**Fig. 1:**
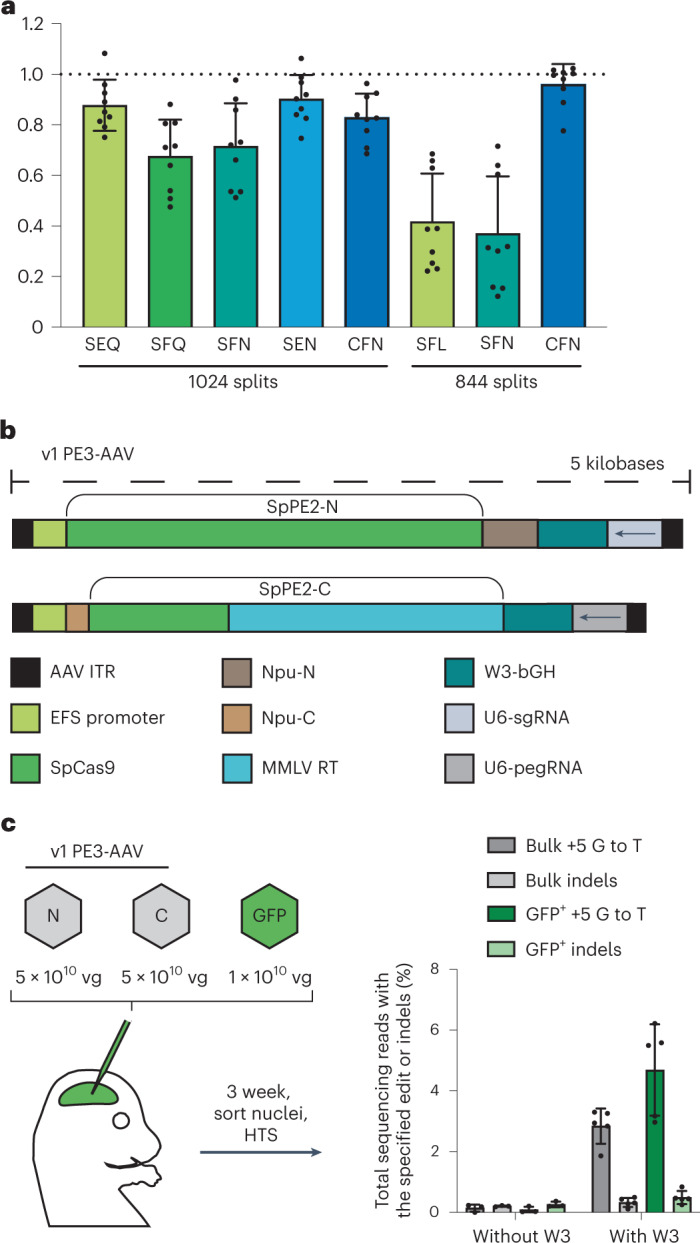
Initial development of a split PE in tissue culture and for AAV-mediated in vivo prime editing. **a**, Editing performance at three genomic loci of intein-split PE3 variants normalized to that of unsplit, canonical PE3 when delivered by plasmid transfection in HEK293T cells. The split site in SpCas9 and the identity of the three most N-terminal residues of the C-terminal extein are indicated below each bar. A suboptimal amount of editor plasmid was used to avoid saturating editing efficiencies. Dots represent values normalized to canonical PE3 activity, and error bars represent mean ± s.e.m. of *n* = 3 biological replicates at three genomic loci. **b**, Schematic of v1 PE3-AAV architecture. **c**, In vivo editing activity of v1 PE3-AAV9 with pegRNA encoding the *Dnmt1* +5 G-to-T edit delivered to neonatal C57BL/6 pups by P0 ICV injection at a dose of 1 × 10^11^ vg total (5 × 10^10^ vg per half). Cortex (neocortex and hippocampus) was harvested; nuclei were isolated and sorted by FACS into bulk and GFP^+^ populations; and genomic DNA was harvested and analyzed by HTS. Dots represent individual mice, and error bars represent mean ± s.e.m. of *n* = 3–5 mice; each condition includes both male and female mice.

**Extended Data Fig. 1:**
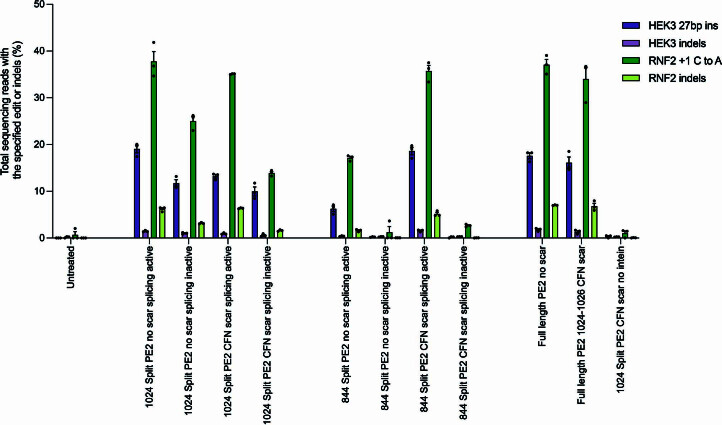
Dependence on splicing of split-intein PE2 activity. Full-length or intein-split PE2 were transfected into HEK239T cells with epegRNA and nicking sgRNA then analyzed by HTS three days after transfection. Splicing-inactive inteins contained a Cys1→Ala mutation in NpuN. A sub-optimal amount of editor plasmid was used to avoid saturating editing efficiencies. Two sites per condition are shown (*HEK3* 27bp insertion and *RNF2* +1 C to A). Dots represent values and error bars represent mean±SEM of *n* = 3 biological replicates at two genomic loci.

To deliver PE via AAV, we split the coding sequence of PE into two halves, one containing the N-terminus of the PE and the other containing the C-terminus, each fused to the N-terminal or C-terminal intein from *Nostoc punctiforme*^32–34,38^, respectively. We nominated positions 844 and 1024 as putative split sites that we hypothesized would be tolerant of structural modification (Supplementary Note 1) and would allow splitting of the PE into two halves, each of which could fit within the packaging limit of AAV while accommodating space for promoters, terminators, a pegRNA cassette and a single guide RNA (sgRNA) cassette. To maximize splicing efficiency, we also assessed mutation of the three N-terminal amino acids of the C-terminal extein from the native residues of Cas9 to the native *Npu* DnaB Cys-Phe-Asn sequence or to intermediate sequence variants. We transfected plasmids encoding the two candidate halves or full-length PE2 along with pegRNA and nicking sgRNA into HEK293T cells and measured editing efficiencies across three sites by high-throughput sequencing (HTS) (Fig. 1a). Among the eight split designs tested, average prime editing efficiencies ranged from 37% to 96% of full-length PE2 activity. The 1024-CFN and 844-CFN split designs yielded robust editing efficiencies (Fig. 1a and Extended Data Fig. 1), consistent with other reports showing that the 1024-NpuN split site reconstitutes prime editing activity^18,21^. Split designs were dependent on the presence and catalytic activity of the intein (Supplementary Note 1 and Extended Data Fig. 1).

### Initial in vivo prime editing with intein-split dual PE-AAV

We chose the 1024-CFN PE split in our initial PE-AAV architecture because this split allows packaging of the PE gene across two AAV genomes, with a U6 promoter-driven pegRNA and nicking sgRNA cassettes for PE3 applications (Fig. 1b). To remain within the ~4.7-kb packaging capacity of AAV^30,31^, we used the small constitutive EFS promoter with a bGH polyadenylation signal^39^. To assess whether mRNA transcript-stabilizing *cis*-elements on the AAV genome would increase PE efficiency, we tested the effect of including W3, the minimized gamma portion of the woodchuck hepatitis virus post-transcriptional regulatory element (WPRE), to the 3′ untranslated region (UTR) of the PE gene^40^. We tested the resulting candidate PE-AAV constructs for their ability to install a +5 G-to-T transversion in the endogenous genomic mouse *Dnmt1* locus that was previously validated in cultured primary neurons^2^ and that is unlikely to cause a phenotypic impact in edited cells^32,41^.

To assess initial in vivo prime editing efficiency, we administered PE-AAVs at a total dose of 1 × 10^11^ viral genomes (vg) (5 × 10^10^ vg each of the N-terminal and C-terminal PE-AAVs) packaged in AAV9 to C57BL/6 mice on postnatal day 0 (P0) via intracerebroventricular (ICV) injection, a method of direct injection into the cerebrospinal fluid that bypasses the blood–brain barrier. To enable the analysis of transduced cells, in addition to bulk tissue, we co-injected a 1 × 10^10^ vg AAV9 expressing EGFP fused to a nuclear membrane-localized Klarsicht/ANC-1/Syne-1 homology (KASH) domain^32,42^ (Fig. 1c). Three weeks after injection, we harvested the cortex from injected and untreated control mice, isolated both FACS-sorted bulk and GFP^+^ nuclei, extracted genomic DNA and analyzed *Dnmt1* editing by HTS. We observed 2.8% and 4.7% editing in bulk and GFP^+^ nuclei, respectively, in brains treated with PE-AAV containing the W3 (Fig. 1c). In mice treated with PE-AAV without W3 (bGH only), editing was very inefficient (≤0.2%; Fig. 1c). These results establish that prime editing is feasible in the mammalian brain when delivered via AAV and that addition of W3 is essential in this context to achieve even low levels of prime editing in the brain. The EFS-driven dual-AAV system with W3 (Fig. 1b) is hereafter referred to as ‘v1 PE3-AAV’.

### Impact of DNA repair on in vivo prime editing efficiency

**Fig. 2:**
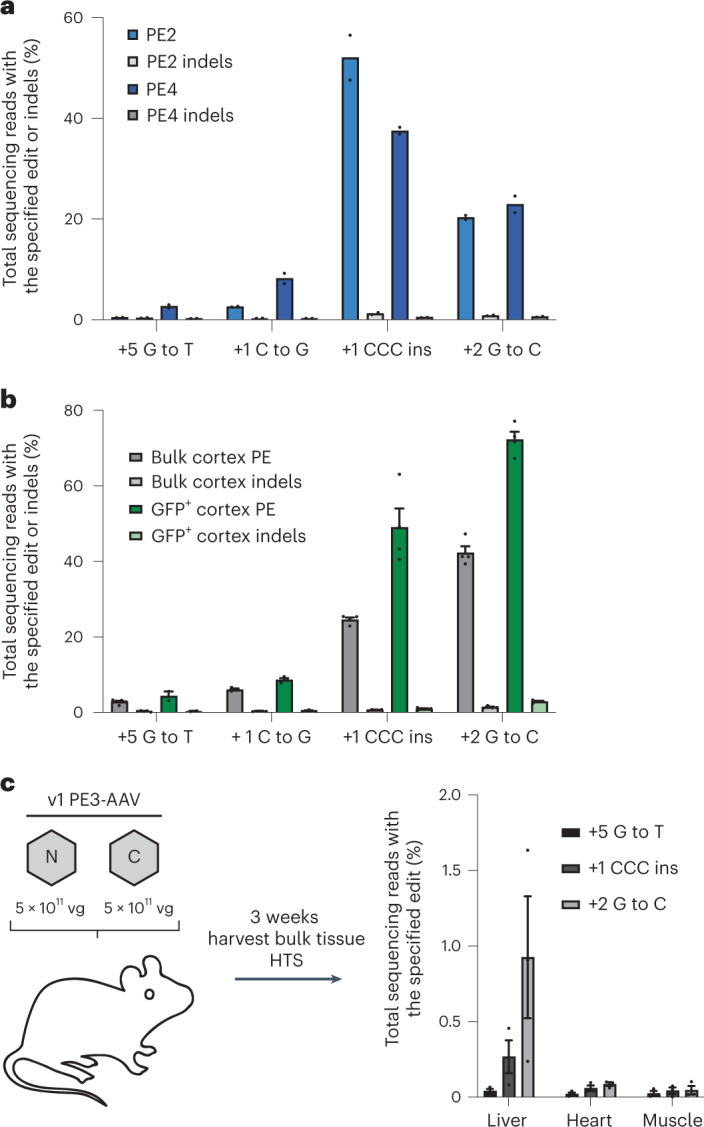
AAV-mediated in vivo prime editing efficiency is dependent on the type of edit. **a**, Editing activity of PE2 and PE4 in N2a cells by plasmid transfection for four different edits at *Dnmt1*. N2a cells were transfected with either PE2 and pegRNA or PE4 (PE2 + MLH1dn) and pegRNA. Three days later, gDNA was harvested and analyzed by HTS. Dots represent values, and bars represent means of *n* = 2 biological replicates. **b**, In vivo editing activity of v1 PE3-AAV9 delivered to neonatal C57BL/6 pups on P0 by ICV at a total dose of 1 × 10^11^ vg (5 × 10^10^ vg per half). Cortex (neocortex and hippocampus) was harvested, nuclei were isolated and sorted by FACS into bulk and GFP^+^ populations, and gDNA was analyzed by HTS. Dots represent individual mice, and error bars represent mean ± s.e.m. of *n* = 3–4 mice; each condition includes both male and female mice. **c**, In vivo editing activity of PE3 delivered via v1 PE3-AAV9 by RO injection to 6–8-week-old C57BL/6 mice at a total dose of 1 × 10^12^ vg. Three weeks after injection, bulk tissues were harvested and gDNA was isolated and analyzed by HTS. Dots represent individual mice, and error bars represent mean ± s.e.m. of *n* = 3 mice; each condition includes both male and female mice.

**Extended Data Fig. 2:**
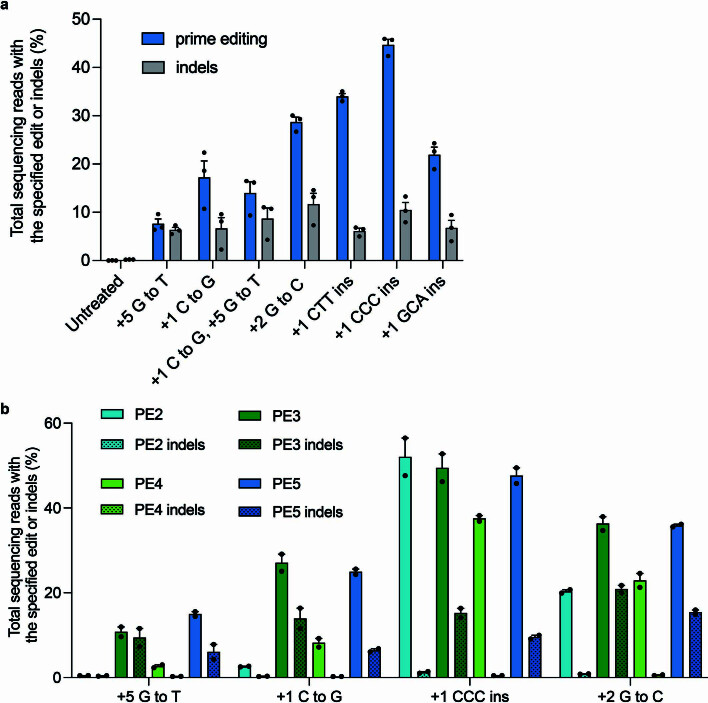
Efficiencies of various prime edits at *Dnmt1* and the effect of transient MMR inhibition. **a**, Mouse N2A cells were transfected with plasmids encoding PE2, a +53 nicking sgRNA, and one pegRNA encoding the edit indicated below each bar. **b**, The impact of MMR recognition of the installed edit on editing efficiency was assessed by installing one of four edits (*Dnmt1* +5 G-to-T, *Dnmt1* +1 CCC insertion, *Dnmt1* +1 C-to-G, or *Dnmt1* +2 G-to-C) as PE2, PE3, PE4 (PE2 + MLH1dn), or PE5 (PE3 + MLH1dn). Data are shown as individual data points and mean±SEM for *n* = 2-3 independent biological replicates.

We recently discovered that DNA mismatch repair (MMR) impedes prime editing efficiency and product purity in cultured cells^43,44^. We sought to evaluate whether the identity of the installed edit and its susceptibility to MMR may limit prime editing in vivo. We characterized a diversity of edits at the *Dnmt*1 locus in mouse N2a cells (Supplementary Note 2) and found that these new edits exhibited higher editing efficiency than the *Dnmt1* +5 G-to-T edit (Extended Data Fig. 2a) and were recognized to different degrees by MMR (Extended Data Fig. 2b). To assess whether MMR-recognized and MMR-evading edits show similar trends in prime editing efficiency in vivo, we performed ICV injections at P0 of 1 × 10^11^ vg of v1 PE3-AAV9 encoding +1 C-to-G, +1 CCC insertion or +2 G-to-C edits. We observed 6.1%, 25% and 42% prime editing for +1 C-to-G, +1 CCC insertion and +2 G-to-C edits, respectively (Fig. 2b), consistent with their relative MMR evasion and efficiencies in N2a cells (Fig. 2a and Extended Data Fig. 2). Although the activity of MMR in the mammalian brain is not well understood, Mlh1 is expressed in the mammalian brain^45–47^ and may limit prime editing in the central nervous system (CNS). These data suggest that MMR or related pathways likely also impede prime editing in vivo and that designing edits to natively evade MMR by adding nearby benign or silent bystander mutations^43^ may enhance prime editing efficiency in the mammalian brain.

### Development of a PE-AAV system for systemic administration

To further explore the utility of split-intein PE-AAVs in vivo, we investigated their ability to mediate prime editing in adult animals via systemic injection. We delivered the v1 PE3-AAV9 encoding the *Dnmt1* +5 G-to-T, +1 CCC insertion or +2 G-to-C edit at a dose of 1 × 10^12^ vg (5 × 10^11^ vg each of the N-terminal and C-terminal AAV) via retro-orbital (RO) injections into 6–8-week-old adult C57BL/6 mice. We harvested liver, heart, and skeletal muscle (tibialis anterior) 3 weeks after injection and analyzed editing by HTS. The +5 G-to-T edit was undetectable (≤0.1%), but we observed low prime editing in the liver for +1 CCC insertion (0.3%) and +2 G-to-C edits (0.9%), respectively (Fig. 2c). No prime editing was observed (≤0.1%) in the heart and skeletal muscle for any of the three edits (Fig. 2c). These results revealed that the PE-AAV architecture required further optimization for systemic injection.

### Factors limiting systemic in vivo PE efficiency

**Extended Data Fig. 3:**
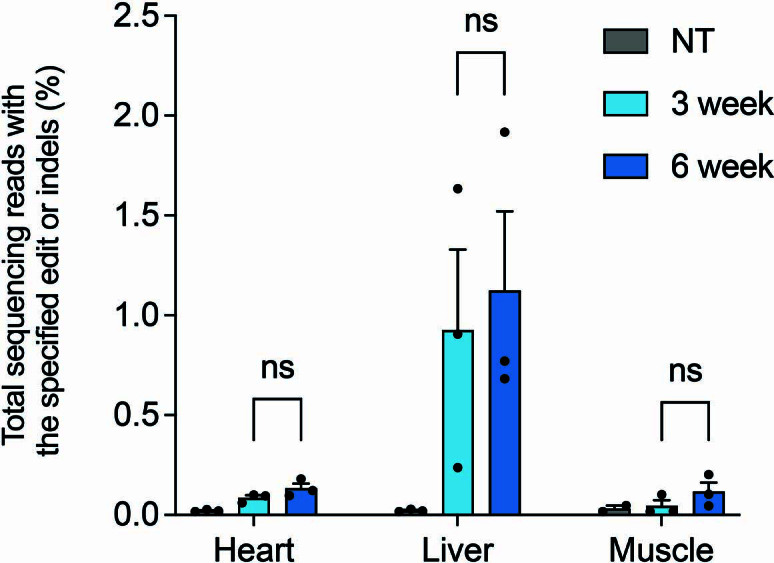
Effect of incubation time on systemic *in vivo* prime editing efficiency. v1 PE-AAV9 encoding the *Dnmt1* +2 G-to-C edit was administered at a dose of 1x10^12^ vg total (5x10^11^ vg per half) by RO injection to 6- to 8-week-old C57BL/6 mice and three weeks after, bulk tissues were harvested and isolated genomic DNA was analyzed by HTS. Dots represent individual mice and error bars represent mean±SEM of *n* = 3 different mice. Significance was calculated by two-tailed unpaired t-test with correction for multiple comparisons.

To understand the factors limiting systemic in vivo prime editing, we first assessed the effect of time after injection on prime editing efficiencies. We administered v1 PE3-AAV9 with pegRNA encoding the *Dnmt1* +2 G-to-C edit to C57BL/6 mice by RO injection and harvested tissues 3 weeks or 6 weeks after injection. We did not observe a significant increase in PE activity with increased AAV expression time, with editing for both conditions yielding ≤1.1%, indicating that time was not the main factor limiting prime editing efficiency (Extended Data Fig. 3).

**Fig. 3:**
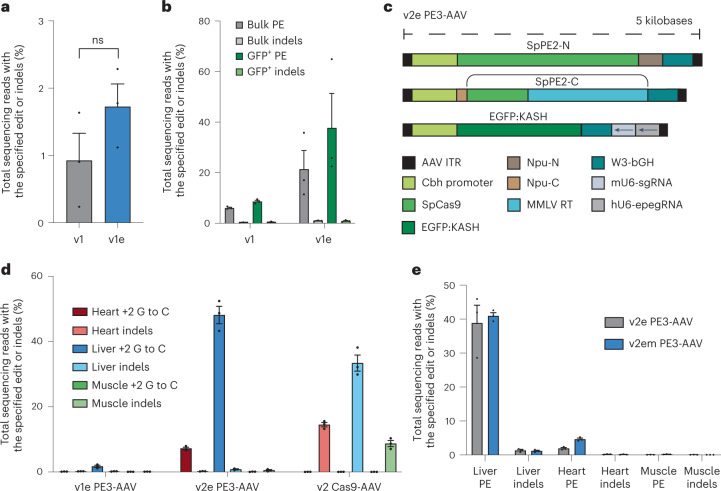
Evaluation of factors limiting systemic prime editing efficiency. **a**, Comparison of unmodified pegRNA or epegRNA installing a +2 G-to-C edit in bulk liver. v1 PE3-AAV9 was delivered by RO injection to 6–8-week-old C57BL/6 mice at a dose of 1 × 10^12^ vg (5 × 10^11^ vg per N-terminal and C-terminal AAVs). Bulk tissue was harvested 3 weeks after injection, and gDNA was isolated and analyzed by HTS. Significance was calculated by unpaired *t*-test. **b**, Comparison of unmodified pegRNA or epegRNA installing a +1 C-to-G edit in cortical tissue. v1 PE3-AAV9 was delivered via P0 ICV injection at a total dose of 1 × 10^11^ vg. Cortex was harvested 3 weeks after injection, nuclei were isolated, and bulk and GFP^+^ populations were isolated by FACS. gDNA was extracted and analyzed by HTS. **c**, Schematic of v2 PE3-AAV. **d**, C57BL/6 mice were RO injected with 1.25 × 10^12^ vg total of either v2 PE3-AAV9 (5 × 10^11^ vg each N-terminal and C-terminal AAVs encoding PEmax plus 2.5 × 10^11^ vg epegRNA/sgRNA AAV encoding the *Dnmt1* +2 G-to-C edit) or architecture-matched v2 Cas9 nuclease AAV9 (5 × 10^11^ vg each N-terminal and C-terminal AAVs encoding Cas9 nuclease plus 2.5 × 10^11^ vg sgRNA AAV). Bulk liver, heart, and muscle tissues were harvested 3 weeks after injection, and gDNA was analyzed by HTS. **e**, Comparison of PE3 and PE3max by v2 AAV9 PE at *Dnmt1* installing the +1 C-to-G edit. For **a**, **b**, **d** and **e**, dots represent individual mice, and error bars represent mean ± s.e.m. of *n* = 3 mice; each condition includes both male and female mice.

To assess whether the use of 3′ stabilized engineered pegRNAs (epegRNAs)^48^ augment prime editing in vivo, we delivered 1 × 10^12^ vg (5 × 10^11^ vg each half) of v1 PE3-AAV9 with either pegRNA or epegRNA (tevopreQ1 fused on the 3′ end of the pegRNA with an 8-nucleotide (nt) linker) via systemic RO injection. The use of an epegRNA resulted in an average of 1.7% +2 G-to-C prime editing of *Dnmt1* in the liver, compared to 0.9% prime editing with unmodified pegRNAs (*P* = 0.20) (Fig. 3a). When assessed in the brain by P0 ICV administration of 1 × 10^11^ vg of v1 PE3-AAV9, epegRNAs more strongly increased *Dnmt1* +1 C-to-G editing efficiencies over pegRNAs from 6.1% to 21% prime editing in bulk cortex and 8.6% to 38% prime editing in GFP^+^ cortex (*P* = 0.10 for both) (Fig. 3b). Although these findings also suggest that pegRNA stability is not the sole factor limiting editing by systemically administered PEs, we used epegRNAs for all subsequent designs and hereafter indicate use of an epegRNA with an ‘e’ (v1e PE3-AAV).

Next, we considered whether insufficient PE protein production driven by the EFS promoter may be a major limiting factor for efficient systemic in vivo prime editing. To improve PE expression, we tested the Cbh promoter, a strong and ubiquitous promoter that mediates efficient AAV-mediated base editing in mice when delivered systemically^32,49^. To accommodate the 0.7-kb Cbh promoter, we moved the pegRNA and sgRNA cassettes to a third AAV vector containing a human U6 promoter that drives epegRNA expression and a mouse U6 promoter that drives nicking sgRNA expression to avoid long stretches of homology on the AAV genome that can lead to recombination^50^ (Fig. 3c). We designated this triple-AAV system ‘v2e PE3-AAV’.

We co-delivered all three v2e PE3-AAV9 PE components at a 1:1:0.5 ratio (5 × 10^11^ vg each of the N-terminal and C-terminal PE-AAVs and 2.5 × 10^11^ vg epegRNA/sgRNA AAV) to 6–8-week-old C57BL/6 mice via systemic RO injection and evaluated prime editing of *Dnmt1* +2 G-to-C edit (Fig. 3d). This strategy yielded 48% prime editing in the bulk liver and 7.2% prime editing in the bulk heart, 27- and 70-fold higher editing compared to v1e PE3-AAV9 that uses the EFS promoter (Fig. 3d). Prime editing in heart was 7.2%, and prime editing in muscle was less than 1%, whereas Cas9 delivered at the same dose yielded 18% and 8.7% indels in heart and muscle, respectively (Fig. 3d), suggesting that cell-type-specific factors or insufficient PE expression may still limit prime editing in some tissues, which can potentially be overcome by increasing AAV dose or by changing delivery route or timing of injection^51^. These data suggest that PE expression level is a major determinant of in vivo prime editing efficiency, consistent with a report of prime editing in the retina^18^.

**Extended Data Fig. 4:**
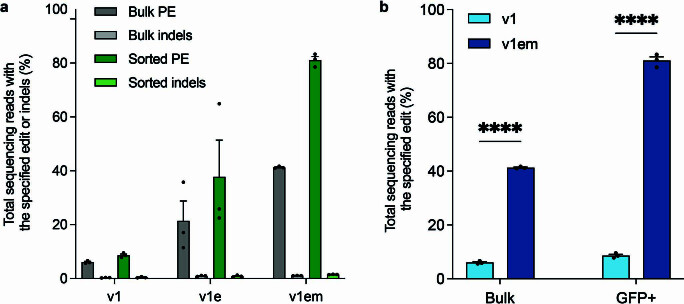
Incorporation of epegRNAs and PEmax together substantially enhance prime editing in CNS. **a-b**, PE3-AAVs encoding a *Dnmt1* +1 C-to-G edit were delivered via P0 ICV injection as v1, v1e, or v1em architecture at a dose of 1x10^11^ vg (5x10^10^ vg each N- and C-terminal AAVs) with 1x10^10^ vg promoter-matched AAV9 EGFP:KASH. At three weeks, nuclei from cortex were isolated and sorted by FACS to either all nuclei (bulk) or GFP+ populations. Genomic DNA was extracted and analyzed by HTS. While improvement from incorporation of epegRNA alone did not reach statistical significance (*P*=0.17), incorporation of epegRNA and PEmax (v1em) yielded highly statistically significant improvement in prime editing over v1 (for both bulk and GFP+ populations, *P*<0.0001). Significance was calculated by two-tailed unpaired t-test with correction for multiple comparisons. Dots represent individual data points and error bars represent mean±SEM for *n* = 3 different mice.

To further improve efficiency, we tested architectural improvements from PEmax^43^, including codon optimization of MMLV RT for improved expression, mutations in Cas9 for enhanced nickase activity and optimized nuclear localization signals (NLS) into the v2e PE3-AAV design, resulting in v2em PE3-AAV. We delivered all three v2e or v2em PE3-AAV9 vectors in a 1:1:0.5 ratio (5 × 10^11^ vg each of the N-terminal and C-terminal PE-AAVs and 2.5 × 10^11^ vg epegRNA/sgRNA AAV) to 6–8-week-old C57BL/6 mice via systemic RO injection and evaluated the efficiency of the *Dnmt1* +1 C-to-G edit. These improvements from PEmax resulted in a similar level of editing efficiency in the well-edited liver but 2.4-fold higher prime editing efficiency in the heart (*P* = 0.017; Fig. 3e), suggesting that the PEmax improvements improve in vivo editing in tissues that do not reach high editing levels with older PE architectures. In the brain, incorporation of epegRNA and PEmax together resulted in 41% prime editing, corresponding to a 6.8-fold increase in prime editing in bulk cortex compared to PE3 with pegRNA (Extended Data Fig. 4; *P* < 0.0001). We used the PEmax architecture for all subsequent experiments.

The observed liver editing efficiencies, which suggest prime editing in the majority of hepatocytes, would likely be relevant for the study or potential treatment of a variety of genetic diseases in the liver^16,52,53^ and are similar to bulk liver editing efficiencies achieved with optimized AAV-delivered base editors and Cas9 nuclease systems^32,33,39^. These data also represent the highest AAV-mediated in vivo liver prime editing efficiencies reported thus far, to our knowledge^15–21^.

### Highly active dual-AAV PE for systemic editing in adult mice

**Fig. 4:**
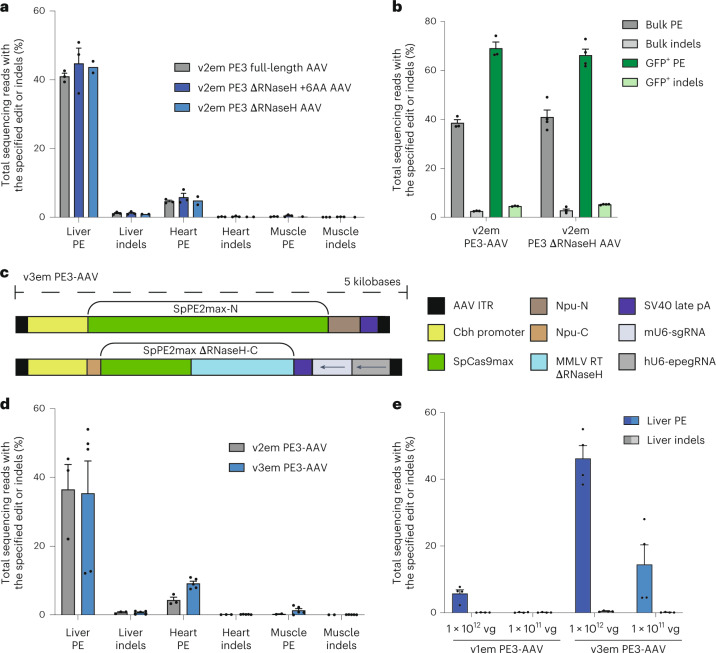
Development of a dual-AAV system for efficient in vivo prime editing. The RNaseH domain of PE3 is not essential in the liver, heart, or brain. **a**, C57BL/6 mice were RO injected with 1.25 × 10^12^ vg total of v2em PE3-AAV9 (5 × 10^11^ vg each N-terminal and C-terminal AAVs plus 2.5 × 10^11^ vg epegRNA/sgRNA AAV) encoding PE3max with its native full-length RT or PE3max with one of two truncated ΔRNaseH RTs to install a *Dnmt1* +1 C-to-G edit. Three weeks after injection, liver, heart, and muscle tissues were harvested and analyzed by HTS. Dots represent individual mice, and error bars represent mean ± s.e.m. of *n* = 1–3 mice; each condition includes both male and female mice. **b**, C57BL/6 pups were injected on P0 by ICV injection of a total of 5.7 × 10^10^ vg total of v2em PE3-AAV (2.3 × 10^10^ vg each N-terminal and C-terminal AAVs plus 1.1 × 10^10^ vg epegRNA/sgRNA AAV) encoding PE3max with full-length RT or PE3max with a truncated ΔRNaseH RT to install the *Dnmt1* +2 G-to-C edit. Three weeks after injection, mice were harvested; cortex was dissected; and nuclei were isolated and sorted by FACS. The bulk population (all nuclei) and a GFP^+^ subpopulation were lysed, and gDNA was analyzed by HTS. Dots represent individual mice, and error bars represent mean ± s.e.m. of *n* = 3–4 mice; each condition includes both male and female mice. **c**, Schematic of v3em PE3max AAV. **d**, C57BL/6 mice were RO injected with either 1.25 × 10^12^ vg total of v2em PE3 ΔRNaseH AAV9 (5 × 10^11^ vg each N-terminal and C-terminal AAVs plus 2.5 × 10^11^ vg epegRNA/sgRNA AAV) or 1 × 10^12^ vg total of v3em PE3-AAV9 (5 × 10^11^ vg each N-terminal and C-terminal AAVs), both installing the *Dnmt1* +1 C-to-G edit. Three weeks after injection, liver, heart, and muscle tissues were harvested and analyzed by HTS. Dots represent individual mice, and error bars represent mean ± s.e.m. of *n* = 3–5 mice; each condition includes both male and female mice. **e**, Prime editing efficiency in bulk liver of v1em or v3em PE3-AAV9 after a single injection of AAV at two different doses. C57BL/6 mice were RO injected with 1 × 10^11^ vg or 1 × 10^12^ vg total of either v1em or v3em PE3-AAV9 containing an epegRNA encoding the *Dnmt1* +2 G-to-C edit. Three weeks after injection, liver tissue was harvested and analyzed by HTS. Dots represent individual mice, and error bars represent mean ± s.e.m. of *n* = 4 mice; each condition includes both male and female mice.

**Extended Data Fig. 5:**
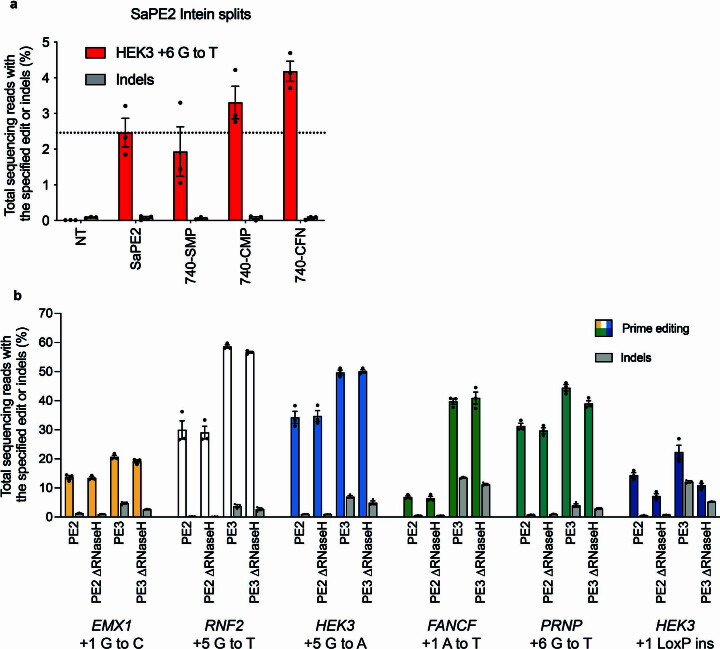
Efforts to minimize the size of the prime editor protein. Activity of full-length and intein-split SaPE2 by plasmid transfection in HEK293T cells with a pegRNA encoding the *HEK3* +6 G-to-T edit. An intein split at previously validated position 740^1^ was assessed to maintain on-target editing efficiency compared to full-length (SaPE2). The three N-terminal residues of the C-terminal extein are indicated, with ‘SMP’ being the native residues to SaCas9, and CFN as the consensus residues of Npu intein. **b**. PE ∆RNaseH maintains prime editing activity in cultured cells across a variety of edits. HEK293T cells were transfected with plasmids encoding PE2 or PE2 ∆RNaseH and pegRNA (PE2) or pegRNA and nicking sgRNA (PE3). Genomic DNA was harvested after three days and analyzed by HTS. Dots represent individual data points and error bars represent mean±SEM for *n* = 3 independent biological replicates.

We next sought to design a dual PE3-AAV system to simplify delivery and reduce the total AAV dose while preserving the improvements that enhanced in vivo prime editing efficiency of the triple-AAV v2em PE3 system. To reduce the size of the prime editing system to fit into two AAV, we considered using smaller Cas variants, such as SaCas9 (Supplementary Note 3 and Extended Data Fig. 5a), but chose to focus on SpCas9-based PEs owing to their more robust editing activity. To minimize the size of SpCas9-based PE, we assessed truncated MMLV RT variants that lack the RNaseH domain (Supplementary Note 3 and Extended Data Fig. 5b). To directly assess whether PE ΔRNaseH maintains its full-length activity in vivo after systemic injection, we used the v2em PE3-AAV system to compare full-length PE programmed to edit *Dnmt1* +1 C-to-G with two variants of RNaseH-deleted PEs. The first ΔRNaseH variant uses a previously published truncation at residue 497 of MMLV RT that appends six additional amino acids^54^. The second RT ΔRNaseH variant is a clean truncation at residue 497 of MMLV RT without additional amino acids. All three PEmax variants (PEmax, PEmax ΔRNaseH+6AA, and PEmax ΔRNaseH) delivered systemically as v2em PE3-AAVs injected at 1.25 × 10^12^ vg total with AAV9 (5 × 10^11^ vg each of the N-terminal and C-terminal PE-AAVs plus 2.5 × 10^11^ vg epegRNA/sgRNA AAV) into 6–8-week-old mice were similarly active in the liver and heart (Fig. 4a), indicating that PE3max ΔRNaseH maintains full-length prime editing efficiency in vivo at the target site assessed. Similarly, when PEmax or PEmax ΔRNaseH v2em PE3-AAVs were injected by P0 ICV injection at 5 × 10^10^ vg total with AAV9 (2.5 × 10^10^ vg each N-terminal and C-terminal PE3-AAV plus 1.1 × 10^10^ vg epegRNA/sgRNA AAV), PEmax ΔRNaseH performed similarly to PEmax (Fig. 4b), indicating that the RNaseH domain is also not necessary for prime editing in the brain.

We next optimized the AAV genome architecture to enable packaging of intein-split PE3max ΔRNaseH into two AAV genomes by retaining the Cbh promoter and substituting with SV40 late poly(A), the size of which allows the pegRNA and nicking sgRNA cassettes to both fit in the C-terminal PE-AAV, resulting in v3em PE3-AAV (Fig. 4c). We compared the performance of v2em PE3-AAV9 with v3em PE3-AAV9, each with an epegRNA programmed to install the +1 C-to-G edit at *Dnmt1* in 6–8-week-old C57BL/6 mice via systemic RO injection. Doses were 1.25 × 10^12^ vg total v2em PE3-AAV (5 × 10^11^ vg each N-terminal and C-terminal PE-AAV and 2.5 × 10^11^ vg epegRNA/sgRNA AAV) or 1 × 10^12^ vg of total v3em PE3-AAV (5 × 10^11^ vg each of the N-terminal and C-terminal v3em PE3-AAVs). Three weeks after injection, we harvested tissues for analysis by HTS. In bulk liver tissue, v3em PE3-AAV9 yielded efficiencies similar to v2em PE3-AAV9 (35% for v3em PE3-AAV versus 36% for v2em PE3-AAV). In bulk heart and skeletal muscle, prime editing increased 2.1-fold (9.1%, *P* = 0.026) and 4.9-fold (1.3%, not significant by unpaired *t*-test), respectively, for v3em compared to v2em PE3-AAV (Fig. 4d). The largely unchanged editing efficiency in liver but strong improvement in extra-hepatic tissues upon reduction of the co-transduction requirement likely reflects that transduction efficiency in liver is only modestly limiting for either AAV system^39^, in contrast with less efficiently transduced tissues in which AAV delivery remains a key bottleneck.

**Extended Data Fig. 6:**
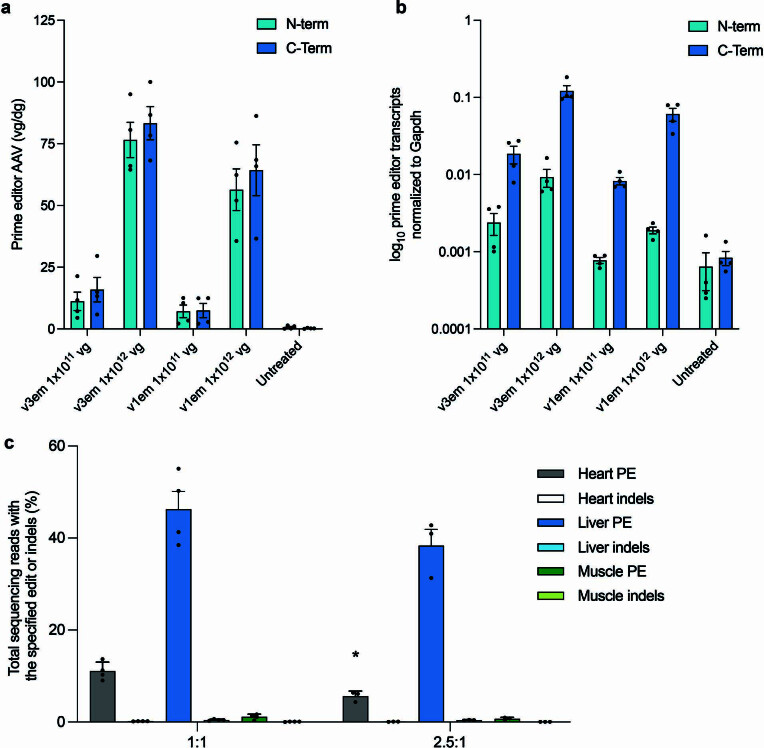
Transduction, RNA expression, and assessment of higher N:C ratio of PE-AAVs in liver. **a**, *In vivo* comparison of viral genomes per diploid genome by promoter and dose in liver tissue. Prime editors were expressed from either the v3em (Cbh promoter) or v1em (EFS promoter) architecture at the doses indicated. Isolated DNA was quantified by ddPCR using primers and a probe specific to either the N- or C-terminal half of SpCas9. Viral genome concentrations were normalized to *Gapdh*. **b**, *In vivo* comparison of PE transcript levels by promoter and dose in liver tissue. Prime editors were expressed from either the v3em (Cbh promoter) or v1em (EFS promoter) architecture at the doses indicated. Mature mRNA transcripts were reverse transcribed, and PE transcript quantities from the resulting cDNA were measured by droplet digital PCR (ddPCR) using primers and a probe specific to either the N- or C-terminal half of SpCas9 (Supplementary Table 4). Transcript concentrations were normalized to *Gapdh*. **c**, *In vivo* comparison of higher ratio of N-terminal v3em PE-AAV to C-terminal half v3em PE-AAV. v3em PE3-AAV9 encoding the *Dnmt1* +2 G-to-C edit was delivered at a total dose of 1×10^12^ vg at the ratio indicated: either a ratio of 1:1 (5×10^11^ vg each N- and C-terminal halves) or a ratio of 2.5:1 (7.1×10^11^ vg N-terminal half and 2.9×10^11^ vg C-terminal half) and bulk tissues were analyzed three weeks post injection by HTS. Dots represent individual mice and error bars represent mean±SEM of *n* = 3-4 different mice.

Next, we directly compared v1em and v3em PE3-AAV architectures. We RO injected 6–8-week-old C57BL/6 mice with v1em or v3em PE3-AAV9, each at a high dose of 1 × 10^12^ vg total (5 × 10^11^ vg N-terminal and C-terminal PE-AAVs) or a 10-fold lower dose of 1 × 10^11^ vg total (5 × 10^10^ vg N-terminal and C-terminal PE-AAVs) and with each epegRNA encoding the *Dnmt1* +2 G-to-C edit. Three weeks after injection, DNA and RNA were isolated from bulk liver tissue. We observed 46% and 14% prime editing with v3em PE3-AAV9 at the high dose and low dose, respectively (Fig. 4e), and 5.7% and 0.1% prime editing with v1em PE3-AAV9 in bulk liver, consistent with the observed improved PE mRNA expression from the Cbh promoter (Supplementary Note 4 and Extended Data Fig. 6).

**Extended Data Fig. 7:**
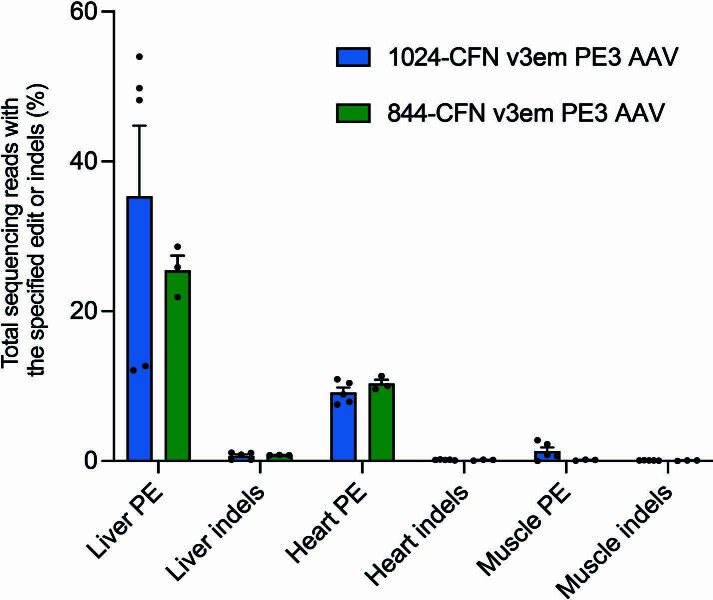
Comparison of v3em PE3-AAV split at two different positions. 6- to 8-week-old C57BL/6 mice were injected with 1x10^12^ vg total of v3em PE3-AAV9 (5x10^11^ vg each N & C terminal AAVs) using the intein-split indicated (residue 1024 or 844, in both cases with extein residues mutated to CFN) installing a *Dnmt1* +1 C-to-G edit. Dots represent individual mice and error bars represent mean±SEM of *n* = 3-5 mice.

Finally, we assessed v3em PE3-AAVs using the 844-CFN intein split architecture that also yielded high-efficiency prime editing in vitro (Fig. 1a and Extended Data Fig. 7). We delivered 1 × 10^12^ vg of total 1024-CFN or 844-CFN v3em PE3-AAV (5 × 10^11^ vg each of the N-terminal and C-terminal PE-AAVs) to 6–8-week-old C57BL/6 mice via systemic RO injection and analyzed prime editing 3 weeks after injection. Editing efficiency differences were statistically insignificant by unpaired two-tailed *t*-test (Extended Data Fig. 7). Thus, 884-CFN offers an alternate PE protein split that can accommodate other elements on the AAV genome that may be necessary for different applications. We continued to use the 1024-CFN (v3em PE-AAV) for further characterization.

### Improved PE-AAV prime editing in the CNS

**Fig. 5:**
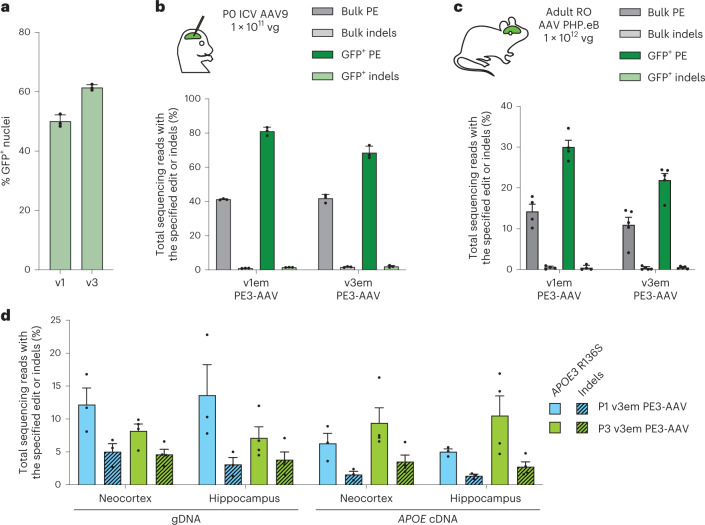
Improved prime editing in the mouse CNS. Comparison of prime editing from v1em and v3em PEmax AAVs in the CNS via direct injection in neonatal mice or systemic injection in adult mice. **a**, Percent GFP^+^ nuclei of capsid-matched, promoter-matched and terminator-matched EGFP:KASH used for enrichment. Dots represent individual mice, and error bars represent mean ± s.e.m. of *n* = 3 mice; each condition includes both male and female mice. **b**, Either v1em or v3em PE3-AAV9 encoding the +1 C-to-G edit at *Dnmt1* was delivered via P0 ICV injection at a dose of 1 × 10^11^ vg. Cortex was harvested after 3 weeks, and nuclei were isolated. GFP^+^ subpopulations were sorted by FACS. gDNA from bulk or GFP^+^ nuclei was extracted and analyzed by HTS. Dots represent individual mice, and error bars represent mean ± s.e.m. of *n* = 3 mice; each condition includes both male and female mice. **c**, Adult 6–8-week-old mice were RO injected with a total dose of 1 × 10^12^ vg AAV PHP.eB with either v1em or v3em PE3-AAV encoding the +2 G-to-C edit at *Dnmt1*. Cortex was harvested after 3 weeks, and nuclei were isolated. GFP^+^ subpopulations were sorted by FACS. gDNA from bulk or GFP^+^ nuclei was extracted and analyzed by HTS. Dots represent individual mice, and error bars represent mean ± s.e.m. of *n* = 4–5 mice; each condition includes both male and female mice. **d**, Installation of *APOE3* R136S (*APOE* Christchurch) in humanized *APOE3* mice. 1 × 10^11^ vg v3em PE3-AAV9 was administered by ICV injection to mice on P1 or P3. gDNA or total RNA was isolated from neocortex and hippocampus (matched hemispheres). RNA was converted to cDNA, and both gDNA and cDNA were analyzed by HTS. Dots represent individual mice, and error bars represent mean ± s.e.m. of *n* = 3–4 mice; each condition includes both male and female mice.

**Extended Data Fig. 8:**
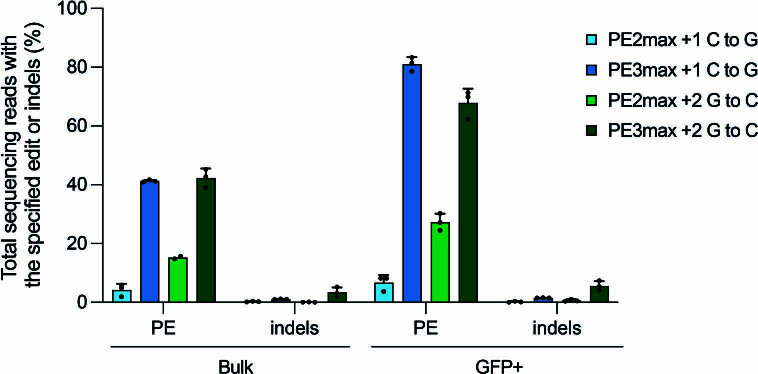
Comparison of PE2 and PE3 *in vivo*. v1em AAV9 with an epegRNA encoding a +1 C-to-G edit or +2 G-to-C edit at *Dnmt1* was delivered via P0 ICV injection at a dose of 1x10^11^ vg (5x10^10^ vg each N- and C-terminal AAVs) with 1x10^10^ vg promoter-matched AAV9 EGFP:KASH. At three weeks, nuclei from cortex were isolated and sorted by FACS to either all nuclei (bulk) or GFP+ populations. Genomic DNA was extracted and analyzed by HTS. Dots represent individual mice (*n* = 2-4) and error bars represent mean±SEM of *n* = 3-4 mice.

Although v1 PE3-AAV supported robust CNS prime editing when injected directly to the brain in neonatal mice (Figs. 2b and 3b), improvements in v3em PE3-AAV design might offer increased CNS editing efficiency over v1em PE3-AAV. To test this possibility, we delivered PE3max with epegRNA installing the *Dnmt1* +1 C-to-G edit via P0 ICV injection using the v1em or v3em PE3-AAV9 architecture at a dose of 1 × 10^11^ vg (5 × 10^10^ vg per half) with 1 × 10^10^ vg capsid-matched, promoter-matched and terminator-matched EGFP:KASH to facilitate sorting of transduced cells. The capsid-, promoter-, and terminator-matched GFP AAVs yielded 50% and 61% GFP^+^ nuclei for v1 and v3, respectively (Fig. 5a), indicating that the Cbh promoter may be active in more cells across the CNS than the EFS promoter. Although both approaches led to similarly high levels of editing in bulk cortex (41% and 42% for v1em and v3em, respectively), v1em PE3-AAV yielded higher editing among GFP^+^ nuclei (81% for v1em and 69% for v3em, *P* = 0.0087) (Fig. 5b). We also assessed the importance of inclusion of a nicking sgRNA in vivo (the PE3 strategy) and found that PE3 increased editing over PE2 by 9.8-fold and 2.8-fold in bulk cortex for the *Dnmt1* +1 C-to-G edit and +2 G-to-C edit, respectively (Supplementary Note 5 and Extended Data Fig. 8).

To test whether delivery of PEs in adult animals via systemic injection with a blood–brain-barrier-crossing capsid might enable efficient CNS editing, we injected C57BL/6 mice with a total dose of 1 × 10^12^ vg of either v1em or v3em PE3-AAV PHP.eB^55–57^ encoding an epegRNA to install the *Dnmt1* +2 G-to-C edit (5 × 10^11^ vg each half) with promoter- and terminator-matched 1 × 10^11^ vg AAV PHP.eB EGFP:KASH to facilitate enrichment of transduced nuclei (Fig. 5c). With v1em PE3-AAV PHP.eB, we observed 14% prime editing in bulk cortex and 30% prime editing among GFP^+^ nuclei, with few indels (0.5%), whereas v3em PE3-AAV PHP.eB yielded 11% prime editing and 0.4% indels in bulk cortex and 22% prime editing and 0.5% indels in the GFP^+^ population (Fig. 5c). Taken together, these results demonstrate prime editing of the CNS in adult mice after a systemic injection of a blood–brain-barrier-crossing AAV capsid.

### Installation of a disease-relevant mutation in the CNS

To test the capabilities of PE-AAV systems to edit mutations of biomedical interest in vivo, we first used PE-AAVs to install the putatively protective *APOE* Christchurch *(APOE3* R136S) coding variant, a G-to-T transversion mutation that cannot be installed via base editing and that would be difficult to install in post-mitotic cells via homology-directed repair (HDR). This mutation is of biological and therapeutic interest, as it has been observed in an individual who carried the risk-associated *PSEN1* (presenilin 1) E280A mutation but who did not develop cognitive impairment until three decades after the expected age of onset of AD among *PSEN1 E280A* carriers^58^. A subsequent study suggests that the *APOE* Christchurch variant may be deleterious in other genetic contexts^59^. The ability to precisely install the *APOE* Christchurch allele in relevant cells in vivo could help illuminate the mutation’s influence on AD pathology.

To develop a strategy for the installation of *APOE3* R136S, we first optimized a prime editing strategy for this mutation in HEK293T cells, achieving 40% prime editing by plasmid transfection (Supplementary Fig. 1a), and verified prime editing in cultured humanized mouse astrocytes, observing 15% prime editing with PE3 and 27% prime editing with PE5 (Supplementary Fig. 1d). These results indicate that prime editing is feasible in cultured astrocytes, the major apoE-expressing cells in the CNS and the primary cell type of interest for this mutation^60,61^. To assess installation of *APOE3* R136S in vivo, we performed ICV injection of 1 × 10^11^ vg (5 × 10^10^ vg per half) of v3em PE3-AAV9 carrying the optimized epegRNA and nicking sgRNA to humanized *APOE3* mice via ICV injection. We also assessed the impact of injection timing by injecting via ICV at P1 or P3, as the extent of non-neuronal cell transduction is known to increase with age^62–64^. Three weeks after injection, we analyzed prime editing efficiencies in bulk nuclei from neocortex and hippocampus, two brain regions relevant to AD pathology. DNA editing efficiencies in bulk neocortical and hippocampal tissues for P1 injected mice were 12% prime editing with 5.0% indels and 14% prime editing with 3.1% indels, respectively, whereas P3 injections resulted in 8.2% prime editing with 4.6% indels and 7.1% prime editing with 3.8% indels in the bulk neocortex and hippocampus tissues, respectively (Fig. 5d). To measure installation of *APOE3* R136S in apoE-expressing cells, we isolated total RNA from AAV-injected and control brain tissues 3 weeks after injection, generated cDNA and observed 9.4% prime editing with 3.5% indels in neocortex *APOE* cDNA and 11% prime editing with 2.8% indels in hippocampal *APOE* cDNA. Together, these data reveal that prime editing can install mutations of therapeutic interest in relevant CNS cell types in vivo.

### Installation of a protective mutation in adult mouse liver

To further assess in vivo editing activity of PE-AAV systems, we tested their ability to mediate therapeutically relevant prime edits in adult animals. We targeted *Pcsk9*, a gene involved in cholesterol homeostasis^65–70^. Although the use of genome editing to knock down PCSK9 has yielded encouraging results^39,71–73^, and base editing of *PCSK9* to lower LDL cholesterol levels has recently entered clinical trials^74^, the ability of prime editing to precisely introduce virtually any small substitution, insertion or deletion provides access to *PCSK9* variants that could offer unique strengths.

To test whether PE-AAV could edit the liver of an adult animal to confer a protective phenotypic change, we designed and produced PE-AAV to install the mouse homolog of *PCSK9* Q152H, a G-to-C substitution that blocks autocatalytic processing of PCSK9 (ref. ^75^) with retention of both the mutated protein as well as autocatalytically competent wild-type PCSK9 in the endoplasmic reticulum (ER) without inducing ER stress or the unfolded protein response in humans or in mice overexpressing *PCSK9* Q152H^76^. Individuals homozygous for *PCSK9* Q152H show a marked reduction in LDL cholesterol levels, and heterozygous individuals also show a reduction in levels of circulating PCSK9 and LDL^75,77^. The development of an in vivo prime editing strategy to install this mutation in postnatal animals may help illuminate its physiological consequences and test a potential therapeutic strategy to lower coronary heart disease risk.

We first optimized prime editing in cultured cells to install the mouse homolog of *PCSK9* Q152H (*Pcsk9* Q155H) (Supplementary Fig. 2a–c). We transfected plasmids encoding PEmax, an epegRNA and a nicking sgRNA, resulting in up to 17% prime editing in mouse N2a cells (Supplementary Fig. 2a–c). We next assessed whether the inclusion of MMR-evading silent mutations near the intended edit^43^ could improve installation of *Pcsk9* Q155H and found a 2.8-fold improvement when the silent mutation was incorporated with PE2max and 1.3-fold with PE3max (Supplementary Fig. 2d), achieving up to 25% prime editing with 5.5% indels in N2a cells.

We then delivered v3em PE3-AAV9 into 6–8-week-old mice via systemic RO injection at a total dose of 1 × 10^12^ vg per mouse and compared epegRNAs encoding *Pcsk9 Q155H* with a PAM-disrupting edit or with MMR-evading silent edits (Supplementary Fig. 2e), achieving 31% and 38% prime editing, respectively. Inclusion of MMR-evading silent edits modestly increased average liver editing efficiency 8 weeks after injection (*P* = 0.06, unpaired *t*-test) (Supplementary Fig. 2e). Together, these findings demonstrate that MMR-evading silent edits can increase prime editing efficiency in vitro and in vivo and should be considered for the design of prime editing experiments in animals when possible.

**Fig. 6:**
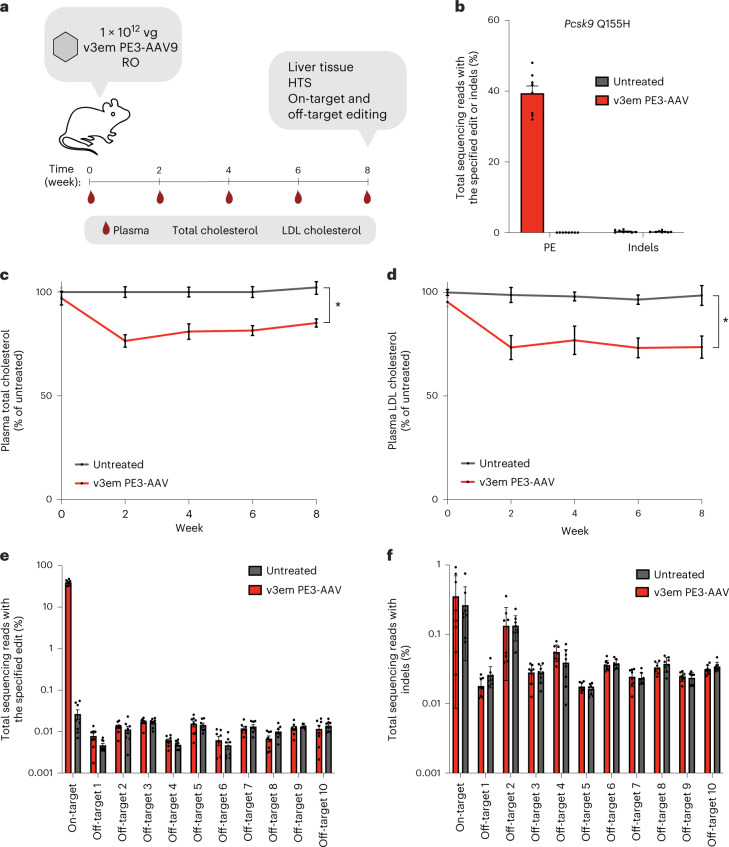
In vivo prime editing to install *Pcsk9* Q155H and its effect on plasma cholesterol levels. **a**, Timing of injections of v3em PE3-AAV9, evaluation of prime editing and plasma analysis. **b**, Bulk liver editing efficiencies for installation of *Pcsk9* Q155H with an epegRNA that includes additional MMR-evading silent edits. **c**,**d**, Plasma total cholesterol and plasma LDL cholesterol in mice treated with prime editing AAV normalized to untreated mice at the same age. Data are shown as mean ± s.e.m. for *n* = 8 mice (four male and four female), *P = 0.0064 and *P* = 0.023, respectively. Significance was calculated using two-way repeated-measures ANOVA with Sidak multiple comparisons and is shown for the 8-week timepoint. **e**,**f**, Off-target prime editing for 10 CIRCLE-seq-nominated off-target loci (Off-target1–Off-target10) for pegRNA and nicking sgRNA from the livers of v3em PE3-AAV-treated and untreated mice. For **b**, **e** and **f**, dots represent individual mice, and error bars represent mean ± s.e.m. of *n* = 8 mice (four male and four female).

**Extended Data Fig. 9:**
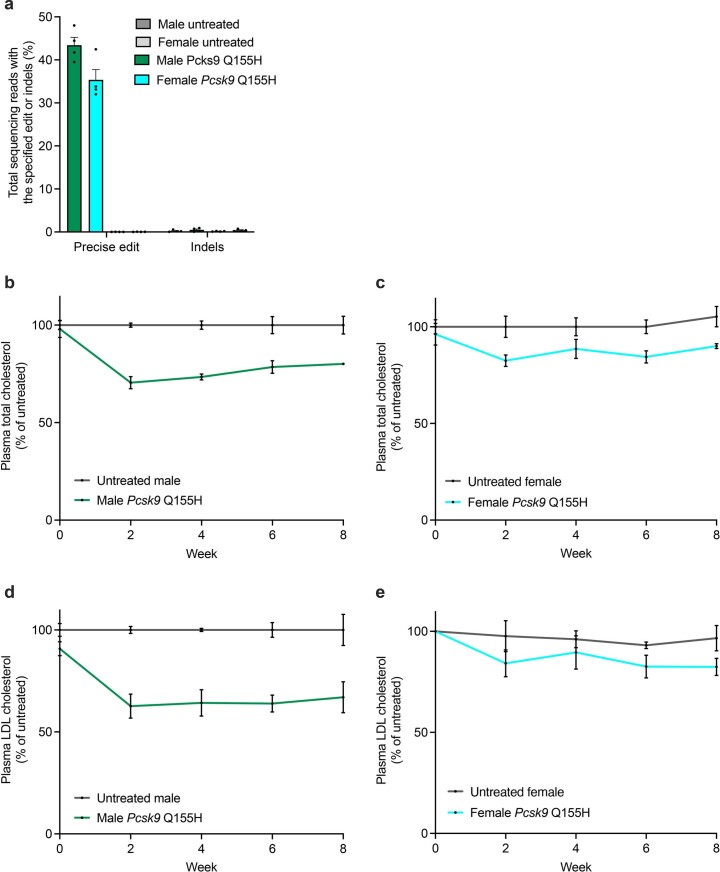
Prime editing and plasma analytes (normalized to untreated) data of either untreated or v3em PE3-AAV9 treated male and female C57BL/6 mice used to plot Fig. 6. **a**, Installation of *Pcsk9* Q155H in mouse liver using v3em PE-AAV9 with epegRNA encoding MMR-evading silent edits used to plot Fig. 6b. Dots represent individual mice and error bars represent mean±SEM for *n* = 4 mice. **b**-**c**, Total plasma cholesterol in C57BL/6 male mice (**b**) and female mice (**c**) normalized to untreated used to plot Fig. 6c. Data are shown as mean±SEM for *n* = 4 mice. **d**-**e**, Plasma LDL cholesterol levels from C57BL/6 male mice **d** and female mice **e** normalized to untreated used to plot Fig. 6d. Data are shown as mean±SEM for *n* = 4 mice.

In a separate cohort of mice, we assessed whether v3em PE3-AAV-mediated *Pcsk9* prime editing with this MMR-evading strategy in liver reduces circulating lipid levels. We delivered v3em PE3-AAV9 into 6–8-week-old mice via systemic RO injection at a total dose of 1 × 10^12^ vg per mouse and achieved 39% editing 8 weeks after injection (Fig. 6b and Extended Data Fig. 9a). Total plasma cholesterol in *Pcsk9* Q155H-edited mice decreased by 20% on average compared to age-matched untreated mice 2 weeks after injection, and this effect persisted until the end of the study at 8 weeks (*P* = 0.022 by two-way ANOVA at 8 weeks) (Fig. 6c). We observed 27% reduction in plasma LDL cholesterol at 2 weeks after treatment compared to age-matched untreated controls, and this effect also persisted until the end of the study at 8 weeks (Fig. 6d) (*P* = 0.023 by two-way ANOVA). Male mice showed a larger reduction in both total plasma cholesterol and LDL cholesterol than age- and condition-matched female mice (Supplementary Note 6, Extended Data Fig. 9 and Supplementary Fig. 3). Although the effect of *PCSK9* Q152H transgene overexpression has been reported^76,77^, the installation of this mutation into the genome in vivo and the examination of the resulting physiological effect has not, to our knowledge, been previously described. Collectively, these results demonstrate that the optimized v3em PE3-AAV architecture can achieve robust and therapeutically relevant prime editing in vivo to install genomic mutations that cannot readily be installed in relevant tissues by other methods.

### Characterization of in vivo off-target editing and toxicity

To assess in vivo off-target prime editing in liver tissue from v3em PE3-AAV treatment, we performed circularization for in vitro reporting of cleavage effects by sequencing (CIRCLE-seq)^78^ to nominate potential off-target loci that might be engaged by either the *Pcsk9* Q155H pegRNA or nicking sgRNA. From the nominated loci, we then selected 10 candidate off-target sites based on the highest read counts from CIRCLE-seq to examine by HTS (Supplementary Tables 5 and 6). No detected off-target editing above background levels was present at any of these loci in mice treated with v3em PE3-AAV9, indicating that prime editing at this site maintains a high degree of sequence specificity, consistent with several other reports^2,79–88^ (Fig. 6e,f).

**Extended Data Fig. 10:**
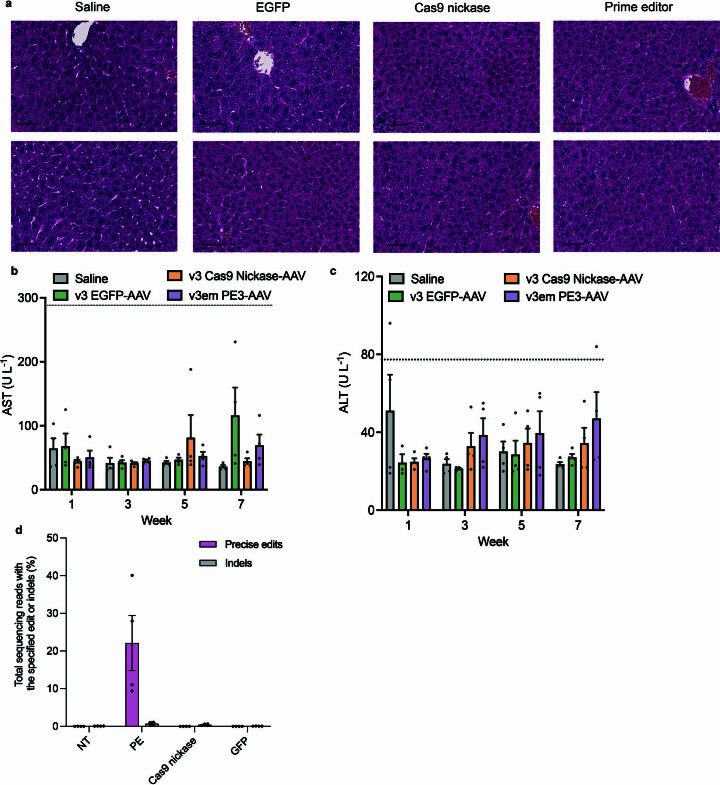
Assessment of liver toxicity following systemic v3em PE-AAV8 injection. Adult mice were systemically injected v3em PE-AAV8 along with dose- and serotype-matched AAV8 encoding EGFP to control for toxicity induced by the AAV vector, dose- and serotype-matched Cas9 nickase to control for toxicity caused by Cas9 nickase and sgRNA portion of the prime editor, and a fourth cohort injected with saline alone. **a**, Histopathological assessment by hematoxylin and eosin staining of livers at eight weeks post-injection of saline or 1x10^12^ vg total AAV8 encoding either EGFP, Cas9 nuclease, or prime editor. Scale bars, 50 μm, representative images from two separate mice shown per condition. **b-c**, Plasma aspartate transaminase (AST) and alanine transaminase (ALT) levels. Data are shown as mean±SEM for *n* = 4 mice. The dotted lines mark the upper limit of normal physiological range for AST and ALT in adult C57BL/6 mice. **d**, Installation of *Pcsk9* Q155H *in vivo* for liver toxicity study. v3em PE3-AAV8 was injected into 6- to 8-week old adult C57BL/6 mice at a dose of 1x10^12^ vg total by retro-orbital injection and liver was harvested eight weeks post injection to assess bulk editing. Dots represent individual mice and error bars represent mean±SEM for *n* = 4 mice.

Finally, we investigated toxicity from v3em PE3-AAV delivery by measuring serum alanine aminotransferase (ALT) and aspartate transaminase (AST) levels, biomarkers of hepatocellular and kidney injury^89^. We used v3em PE3-AAV8, a serotype that efficiently and specifically transduces murine hepatocytes^90^, and delivered into a separate cohort of mice dose- and serotype-matched AAV8 encoding EGFP or Cas9 nickase and a fourth cohort with saline alone. Although we observed a slight elevation of ALT and AST in AAV-injected mice at 5 weeks and 7 weeks after injection compared to saline controls, the levels were within the normal physiological range for all conditions^91,92^, and there were no statistically significant differences between saline-treated and PE-AAV-treated mice (*P* = 0.28 by two-way ANOVA) (Extended Data Fig. 10b,c). Additionally, we performed liver histology 8 weeks after injection and found no evident histological or morphological changes compared to saline-treated mice (Extended Data Fig. 10a). Together, these results demonstrate that our optimized v3em PE3-AAV can mediate efficient prime editing in the mouse liver with no detected off-target editing and without evident toxicity.

## Discussion

By systematically identifying and addressing the factors limiting in vivo prime editing via AAV, we developed a platform for the delivery of PEs to mouse CNS, liver, and heart. Our findings highlight promoter choice and PE expression as key bottlenecks of PE performance in vivo that can be overcome through vector engineering. We also show that recent improvements in prime editing, such as epegRNAs, PEmax and the use of MMR-evading bystander edits, can each improve prime editing in vivo. In the brain and liver, v3em PE-AAVs can achieve ≥40% precise prime editing, substantially outperforming previously reported platforms for in vivo PE delivery in these systems with a clinically validated vector and establishing prime editing in the mammalian brain. PE-AAVs can facilitate the installation of various therapeutic edits, including protective alleles associated with AD and coronary artery disease. We anticipate that properties of optimized v1em and v3em PE-AAV systems will advance the study and potential treatment of a wide variety of diseases with a genetic component.

We limited the doses of systemically delivered AAV in this study to a maximum of 1 × 10^12^ vg per animal (4–5 × 10^13^ vg kg^−1^ for a 20–25-g mouse), a dose considered to be tolerated in clinical trials involving AAV^22,24,93,94^. Prime editing efficiency in extra-hepatic tissues may be further increased by improvements in PE expression using tissue-specific optimization, such as delivery routes that increase AAV transduction in relevant tissues^51^ and the use of capsids with enhanced tropism for a given tissue.

In vivo AAV-mediated prime editing currently requires the use of two or more AAVs. For prime editing applications that require additional proteins, such as installing site-specific recombinase landing sites for gene-sized genomic insertions^2,95,96^, further size reduction of the components could facilitate a dual-AAV strategy. Substantial size reduction of components may enable single-AAV prime editing, which would further simplify use and enhance therapeutic relevance, similar to the improvements that we recently observed from single-AAV base editor delivery^39^.

In vivo prime editing resulted in no detected off-target editing and no significant increase in markers of liver damage compared to control treatments, suggesting that expression of the PE in vivo does not induce apparent hepatotoxicity. Measures to minimize inflammation and immune response to PE components^16^ should be investigated for therapeutic applications. Additional improvements that offer transient delivery, such as lipid nanoparticles (LNPs) or virus-like particles (VLPs), temporally controlled expression of PEs, and further enhanced potency may improve the safety profile of prime editing in vivo.

## Methods

### Molecular biology

Editor plasmids used for mammalian cell transfection were generated using Gibson assembly or USER assembly using pCMV-PE2 (Addgene, 132775) and pCMV-PEmax (Addgene, 174820) plasmids. pegRNA and epegRNA constructs were cloned by Golden Gate assembly using custom pU6-pegRNA-GG-acceptor (Addgene,132777) and pU6-tevopreQ1-GG-acceptor (Addgene, 174038) plasmids. All nicking sgRNA plasmids were generated by KLD assembly or Golden Gate assembly using pFYF1320 (Addgene, 47511) as a template plasmid. rAAV vector plasmids were cloned by restriction digestion of v5 AAV CBE (Addgene, 137176) or v5 AAV ABE (Addgene, 137177) followed by Gibson assembly with eBlocks or polymerase chain reaction (PCR) amplicons. All plasmids used for mammalian tissue culture were purified from MACH1, DH5alpha or NEBstable *Escherichia coli* using Plasmid Plus Maxiprep or Midiprep kits (Qiagen), ZymoPURE II Midiprep Kit (Zymo Research) or PureYield Plasmid Miniprep kit (Promega).

### Cell culture

HEK293T cells (American Type Culture Collection (ATCC), CRL-3216) and Neuro-2A cells (ATCC, CCL-131) were grown in DMEM plus GlutaMAX (Thermo Fisher Scientific) supplemented with 10% (v/v) FBS at 37 °C with 5% CO_2_. Immortalized mouse astrocytes containing the *APOE4* isoform of the human *APOE* gene (Taconic Biosciences) were grown in DMEM plus GlutaMAX (Thermo Fisher Scientific) supplemented with 10% (v/v) FBS and 200 μg ml^−1^ Geneticin (Thermo Fisher Scientific). Cell lines were authenticated by their suppliers and were verified to be mycoplasma negative during the study.

### Transfections of HEK293T and Neuro-2A cells

Sixteen to twenty-four hours before transfection, HEK293T and Neuro-2A cells at more than 90% viability were seeded on 48-well poly-d-lysine-coated plates (BioCoat plates, Corning) at a density of 30,000–40,000 cells per well. Cells were transfected with 1 μl of Lipofectamine 2000 (Thermo Fisher Scientific) and 750 ng of PE plasmid, 250 ng of pegRNA plasmid and 83 ng of sgRNA plasmid. For conditions delivering MLH1dn, 100 ng of additional plasmid was transfected. For 96-well plate (Corning) transfections, 15,000–20,000 cells were plated 16–24 h before transfection, and 200 ng of PE, 40 ng of pegRNA and 13.3 ng of nicking guide with 0.5 μl of Lipofectamine 2000 (Thermo Fisher Scientific) were used. For intein-split PE plasmid transfections, total PE plasmid was reduced to 50 ng. Seventy-two hours after transfection, genomic DNA (gDNA) was isolated with 75–150 μl of lysis buffer (10 mM Tris-HCl pH 8.0, 9.05% SDS, 25 μg ml^−1^ proteinase K (Thermo Fisher Scientific)) at 37 °C for 1 h, followed by 80 °C for 30 min.

### In vitro transcription of PE2 and MLH1dn mRNA

Plasmid templates for in vitro transcription carry an inactivated T7 promoter, 5′ UTR, Kozak sequence, coding sequences and 3′ UTR. Transcription templates were PCR amplified from these plasmids using Phusion U Green Multiplex Master Mix (Thermo Fisher Scientific) with primers that correct the T7 promoter and add a 119-nt poly(A) tail to the 3′ UTR. After purification of the product with QIAquick PCR Purification Kit (Thermo Fisher Scientific), PE2 and hMLH1dn mRNAs were transcribed from these templates using HiScribe T7 High Yield RNA Synthesis Kit (New England Biolabs) with full replacement of UTP with N1-methylpseudouridine-5′-triphosphate (TriLink Biotechnologies) and co-transcriptional capping by CleanCap Reagant AG (TriLink Biotechnologies). mRNA products were precipitated in 2.5 M lithium chloride, washed twice with 70% ethanol, dissolved in nuclease-free water and stored at −80 °C.

### Nucleofection of *APOE4* murine astrocytes

Astrocytes containing *APOE4* isoform of the *APOE* gene (originated from Taconic Biosciences) were nucleofected using program EN-150 with SF Cell Line 4D-Nucleofector X Kit (Lonza). In brief, 200,000 astrocytes were resuspended in 20 μl of buffer with 1 μg of PE2 mRNA, 90 pmol synthetic pegRNA (Integrated DNA Technologies) and 60 pmol synthetic nicking sgRNA (Synthego). For PE5 experiments, 2 μg of hMLH1dn mRNA was also included in the nucleofection. After nucleofection, the cells were diluted to 100 μl of pre-warmed media and recovered for 10 min at 37 °C, followed by plating in 12-well plates. Seventy-two hours after nucleofection, gDNA was harvested.

### HTS and data analysis

Genomic loci of interest were amplified from isolated gDNA via two rounds of PCR with PhusionU or PhusionHS polymerase (Thermo Fisher Scientific). The initial PCR step (PCR1) was done using primers with Illumina adapter overhangs (Supplementary Table 1) with the following conditions: 95 °C for 3 min; 27–30 cycles of 95 °C for 15 s, 61–70 °C (corresponding to the experimentally optimized T_m_) for 20 s and 72 °C for 30 s, followed by 72 °C for 1 min. Unique Illumina sequencing barcodes were added in subsequent PCR2 step, using 1–2 μl of PCR1 as a template with the following conditions: 95 °C for 3 min; nine cycles of 95 °C for 15 s, 61 °C for 20 s and 72 °C for 30 s, followed by 72 °C for 1 min. After PCR2, samples were pooled according to amplicon size and gel purified in a 1% agarose gel using a Qiaquick Gel Extraction Kit (Qiagen). Pooled library concentration was quantified (Qubit dsDNA HS assay kit, Thermo Fisher Scientific) and run on an Illumina MiSeq 300 v2 Kit with 280–300 cycles.

Sequencing reads were demultiplexed using MiSeq Reporter (Illumina). For single base changes, alignment of amplicon sequences to reference sequence was performed using CRISPResso2 (ref. ^97^) in batch mode with ‘-q30’, ‘discard indel reads TRUE’ and ‘qwc’ coordinates spanning the sequence between pegRNA-directed and nicking sgRNA-directed Cas9 cut sites. Indels were calculated as percentage of (discarded reads) / (total aligned reads). Prime editing at a given position was calculated explicitly as: (frequency of specified point mutation in non-discarded reads) × 100 × (100 – (indel reads)) / 100)). For insertions, CRISPResso2 was executed in HDR mode using identical parameters as described above but with an additional parameter ‘e’ specifying sequence of the edited ‘desired’ amplicon. Indels were calculated as percentage of (discarded reads from the reference-aligned sequences and HDR-aligned sequences) / (total aligned reads). In HDR mode, prime editing efficiency was quantified as (HDR-aligned reads without indels) / (number of total reads aligned to the reference amplicon) × 100 × (100 – (indel reads)) / 100)).

### AAV production

HEK293T clone 17 cells (ATCC, CRL-11268) were maintained in DMEM plus GlutaMAX (Thermo Fisher Scientific) with 10% (v/v) heat-inactivated FBS without antibiotic in 150-mm^2^ dishes (Thermo Fisher Scientific) at 37 °C with 5% CO_2_ and passaged every 2–3 days. Cells were split 1:3 18–22 h before polyethyleneimine transfection (PEI MAX, Polysciences) with 5.7 μg of AAV genome plasmid, 11.4 μg of pHelper (Clontech) and 22.8 μg of rep-cap plasmid per plate. Four days after transfection, cells were harvested using a cell scraper (Corning), pelleted by centrifugation at 2,000*g* for 10 min, resuspended in 500 µl of hypertonic lysis buffer per plate (40 mM Tris base, 500 mM NaCl, 2 mM MgCl_2_ and 100 U ml^−1^ salt active nuclease (ArcticZymes)) and incubated at 37 °C for 1 h. Media were then decanted to a new bottle and a 5× solution of poly(ethylene glycol) (PEG) 8000 (Sigma-Aldrich) and NaCl was added to a final concentration of 8% PEG and 500 mM NaCl, incubated on ice for 2 h or overnight and then centrifuged at 3,200*g* for 30 min. The pellet was resuspended in 500 μl of hypertonic lysis buffer per plate and added to the cell lysate. Cell lysates were either stored at 4 °C overnight or taken immediately to ultracentrifugation.

Cell lysates were centrifuged at 3,400*g* for 10 min and added to Beckman Coulter Quick-Seal tubes via 16-gauge, 5-inch needles (Air-Tite N165) in a discontinuous gradient of iodixanol in sequentially floating layers: 9 ml of 15% iodixanol in 500 mM NaCl and 1× PBS-MK (1× PBS plus 1 mM MgCl_2_ and 2.5 mM KCl), 6 ml of 25% iodixanol in 1× PBS-MK and 5 ml each of 40% and 60% iodixanol in 1× PBS-MK with phenol red at a concentration of 1 μg ml^−1^ in the 15%, 25% and 60% layers to visualize layers. Ultracentrifugation was performed using a fixed-angle Ti 70 rotor in a Optima XPN-100 Ultracentrifuge (Beckman Coulter) at 68,000 r.p.m. (340,000*g* for an r_av_ of 65.7 mm) for 1 h or 58,600 r.p.m. (253,000*g* for an r_av_ of 65.7 mm) for 2 h and 15 min at 18 °C. Immediately after centrifugation, 3 ml of solution was removed from the 40–60% iodixanol interface via an 18-gauge needle. Buffer was exchanged for cold PBS with 0.001% F-68 using PES 100 kD MWCO columns (Thermo Fisher Scientific) and concentrated. The AAV solution was sterile filtered using a 0.22-μm filter and then quantified by quantitative PCR (qPCR) (AAVpro Titration Kit version 2, Clontech) and stored at 4 °C until use.

### Animal care

All experiments involving live animals were approved by the Broad Institute Institutional Animal Care and Use Committee (D16-00903; 0048-04-15-2) and were consistent with local, state and federal regulations as applicable, including the National Institutes of Health Guide for the Care and Use of Laboratory Animals. C57BL/6 mice were purchased as adults from The Jackson Laboratory (000664) for all adult RO injections. For neonatal injections, pregnant C57BL/6 females were purchased from Charles River Laboratories (027), and their pups were injected; or, for *APOE4* experiments, mice were originally purchased from Taconic Biosciences (1549) and were bred and maintained in the Broad Institute vivarium. All mice were housed on a 12-h light/dark cycle between 68 °F and 79 °F and 30–70% humidity. Mice had ad libitum access to standard rodent diet and water except for 6-h fasts just before submandibular bleeding for plasma analysis when access to water was still maintained.

### RO injections

AAV was diluted into 100 μl of sterile 0.9% NaCl USP (Fresenius Kabi) before injection. Anesthesia was induced with 3–4% isoflurane. Under anesthesia, the right eye was protruded; the needle of the loaded insulin syringe was inserted into the RO sinus; bevel faced away from the eye; and the syringe was slowly advanced. Directly after injection, a drop of proparacaine hydrochloride ophthalmic solution (Patterson Veterinary) was applied to the eye as an analgesic.

### Neonatal ventricle injections

For neonatal ICV injections, Drummond PCR pipettes were pulled at the ramp test value of a Sutter P1000 micropipette puller for a tip diameter size of approximately 100 μm. The injection solution of 4 μl including a small amount of Fast Green dye was front-loaded. Pups were anesthetized by cryoanesthesia. Then, 2 μl was then freehand injected into each ventricle. Transillumination of the head was then used to verify successful ventricle targeting.

### Mice tissue collection

Mice in this study were sacrificed by CO_2_ asphyxiation, and unperfused tissues were immediately dissected. Bulk tissue was harvested unless otherwise noted. For harvest of brain tissue, hemispheres were split sagittally by razor blade, and then neocortex and hippocampus were isolated with a micro spatula. Tissues were harvested in DNAdvance lysis buffer (Beckman Coulter), and the gDNA was purified.

### Nuclear isolation and sorting

Dissected brain tissue was homogenized in 2 ml of ice-cold EZ-PREP buffer (Sigma-Aldrich) using a glass Dounce homogenizer (Sigma-Aldrich) with 20 strokes of pestle A followed by 20 strokes of pestle B. Homogenized samples were then decanted into a new tube containing an additional 2 ml of EZ-PREP buffer on ice. After 5-min incubation, samples were centrifuged for 5 min at 500*g* at 4 °C. The nuclei pellet was resuspended in 4 ml of ice-cold nuclei suspension buffer (NSB) consisting of 100 µg ml^−1^ BSA (New England Biolabs) and 3.33 µM Vybrant DyeCycle Ruby (Thermo Fisher Scientific) in cold PBS. Nuclei were then centrifuged at 500*g* for 5 min at 4 °C. The nuclei were resuspended in 1–2 ml of NSB, passed through a 35-µm cell strainer and then sorted using a MA900 Cell Sorter (Sony Biotechnology) at the Broad Institute Flow Cytometry Core. See Supplementary Fig. 17 for FACS gating strategy. Nuclei were sorted into DNAdvance lysis buffer (Beckman Coulter), and gDNA was purified.

### Off-target analysis

CIRCLE-seq was performed and analyzed as described previously^78^ save for the following modifications. For the Cas9 cleavage step, guide denaturation, incubation and proteinase K treatment were conducted using the more efficient method described in the CHANGE-seq protocol^98^. Specifically, the sgRNAs with the spacer sequences ‘GCAUGGCUGUCUGGUUCUGU’ (the PCKS9 nicking sgRNA) and ‘GCCAGGUUCCAUGGGAUGCUC’ (used in the PCKS9 pegRNA) were ordered from Synthego with their standard chemical modifications—2′-*O*-methyl for the first three and last three bases and phosphonothioate bonds between the first three and last two bases. A 5′ ‘G’ nucleotide was included with the 20-nt pegRNA spacer sequence to recapitulate the sequence expressed from AAVs. The sgRNAs were diluted to 9 µM in nuclease-free water and re-folded by incubation at 90 °C for 5 min, followed by a slow annealing down to 25 °C at a ramp rate of 0.1 °C per second. sgRNA was complexed with Cas9 nuclease (New England Biolabs) via a 10-min room temperature incubation after mixing 5 µl of 10× Cas9 Nuclease Reaction Buffer provided with the nuclease, 4.5 µl of 1 µM Cas9 nuclease (diluted from the 20 µM stock in 1× Cas9 Nuclease Reaction Buffer) and 1.5 µl of 9 µM annealed sgRNA. Circular DNA from mouse N2a cells was added to a total mass of 125 ng and diluted to a final volume of 50 µl. After 1 h of incubation at 37 °C, proteinase K (New England Biolabs) was diluted four-fold in water, and 5 µl of the diluted mixture was added to the cleavage reaction. After a 15-min proteinase K treatment at 37 °C, DNA was A-tailed, adapter ligated, USER treated, and PCR amplified as described in the CIRCLE-seq protocol^78^. After PCR, samples were loaded on a preparative 1% agarose gel, and DNA was extracted between the 300-bp and 1-kb range to eliminate primer dimers before sequencing on an Illumina MiSeq. Data were processed using the CIRCLE-seq analysis pipeline and aligned to the mouse genome ‘mm10’ with parameters: ‘read_threshold: 4; window_size: 3; mapq_threshold: 50; start_threshold: 1; gap_threshold: 3; mismatch_threshold: 6; search_radius: 30; PAM: NGG; merged_analysis: True’.

### Analysis of PE activity at Cas9 off-target sites

For detailed off-target analysis, the top 10 sites each for pegRNA and sgRNA spacer (20 total sites) with highest read counts were deep sequenced from liver tissues of untreated or v3em PE3-AAV-treated mice. The sequencing reads were then aligned to reference off-target amplicons using CRISPResso2 (ref. ^97^) in batch mode with ‘-q30’, ‘discard indel reads TRUE’, ‘plot_window_size 80’, ‘-w25’, ‘min_alleles_around_cut_to_plot 0.1’ and ‘max_rows_alleles_around_cut_to_plot 600’. Off-target reads were called as leniently as possible to capture all potential reverse transcription product including point mutations, insertions or deletions at the Cas9 nick site. For off-target sites nominated by pegRNA spacer, the 6-nt sequences 3′ of Cas9 nick site (prime-editable target) were compared to the 3′ DNA flap sequence encoded by pegRNA reverse transcription. Any aligned reads with nucleotide sequence within prime editing target window that matches to the nucleotide encoded by pegRNA reverse transcription were noted as off-target reads. Off-target editing efficiencies were, thus, quantified as a percentage of (number of off-target reads) / (number of reference-aligned reads). For off-target sites nominated by nicking sgRNA spacer, insertions or deletions at the Cas9 nick site were quantified as a percentage of (discarded reads) / (total aligned reads).

### ALT and AST assay

Blood was collected via submandibular bleeding in a serum separator tube. The serum was then separated by centrifugation at 2,000*g* for 15 min and stored at −80 °C until the end of the experiment. The samples were sent to IDEXX BioAnalytics for analysis.

### Plasma measurements for total cholesterol and LDL cholesterol

To track total cholesterol and LDL cholesterol levels in plasma, blood was collected using a submandibular bleed in heparin-coated tubes. Plasma was recovered by centrifugation at 2,000*g* for 15 min and stored at −80 °C in one-time-use aliquots until the end of the experiment. Total cholesterol levels were determined using the Total Cholesterol Reagent (Thermo Fisher Scientific) with cholesterol standards from Pointe Scientific. Plasma LDL cholesterol levels were measured using the LDL Cholesterol Kit (WakoChemical).

### Western blot of liver tissues

For western blot analysis, liver samples were lysed in a homogenization buffer (Cell Signaling Technology) containing protease inhibitor cocktail (Sigma-Aldrich). Samples were run on Mini-Protean TGX Gel, 4–15% gradient gels (Bio-Rad), followed by transfer of proteins to nitrocellulose. Antibodies against LDLR (1:1,000) (Proteintech, 10785-1-AP) and β-actin (1:3,000) (Cell Signaling Technology, 8H10D10) were used with LI-COR 926-68070 and LI-COR 926-32211 secondaries. Precision Plus Protein All Blue Prestained Protein Standards (Bio-Rad, 1610373) ladder was used. Secondaries used were LI-COR 926-68070 and LI-COR 926-32211, both at 1:20,000. The western blot data were quantified through densitometry of the bands using LI-COR Odyssey analyzer software.

### Tissue fixation and histology

Mice were sacrificed by CO_2_ asphyxiation, and tissues were immediately harvested. For liver histology, the left medial lobe was dissected and fixed in freshly prepared 4% paraformaldehyde in 1× PBS. Tissues were rocked at 4 °C for 24 h, washed with PBS with 10 mM glycine and then rocked again at 4 °C in PBS with 10 mM glycine for another 24 h. Fixed tissues were then stored at 4 °C until further analysis.

Liver histopathology was performed by the Rodent Histopathology Core of Harvard Medical School. Fixed liver tissue was embedded in paraffin, cut into 5-μm sections and stained with hematoxylin and eosin. Samples were analyzed by a blinded mouse histopathologist.

### Digital droplet PCR

Total DNA was isolated from liver tissue (DNeasy Blood and Tissue Kit, Qiagen). For quantification of viral genomes, digital droplet PCR (ddPCR) was carried out using ddPCR Supermix for Probes (Bio-Rad) with 10 ng of isolated DNA used as template and 6.25 U of HindIII-HF (New England Biolabs) per 25-µl reaction. Droplets were autogenerated, and PCR was performed with an annealing temperature of 57 °C for 2 min for a total of 40 cycles. Droplets were analyzed on a QX200 droplet analyzer, and fluorescence was quantified using QuantaSoft (Bio-Rad). Total RNA was isolated from flash-frozen liver tissue (RNeasy Plus Mini Kit, Qiagen). Isolated RNA was reverse transcribed into cDNA with SuperScript III first-strand synthesis mix with oligo dT primer (Invitrogen). RNA expression analysis was carried out in the same manner but with Gapdh expression reference assay kit (Bio-Rad). Numbers reported are normalized to reference probes in the same reaction. Primer and probe sequences are available in Supplementary Table 4. To ensure minimal DNA contamination in RNA expression analysis, RT negative controls were verified to yield only background positive droplets.

### Statistical analysis

Data are presented as mean and s.e.m. Sample size and the statistical tests used for each experiment are described in the figure legends. No statistical methods were used to pre-determine sample size. Statistical analysis was performed using GraphPad Prism software.

### Reporting summary

Further information on research design is available in the Nature Portfolio Reporting Summary linked to this article.

## Online content

Any methods, additional references, Nature Portfolio reporting summaries, source data, extended data, supplementary information, acknowledgements, peer review information; details of author contributions and competing interests; and statements of data and code availability are available at 10.1038/s41587-023-01758-z.

### Supplementary information

Supplementary Information Supplementary Figs. 1–6, Supplementary Notes 1–6, Supplementary Sequences and Table of Contents also describing supplementary tables (which are provided in a separate Excel file).

Reporting Summary

Supplementary Tables 1–8.

## Data Availability

All pegRNA, nicking sgRNA and HTS primer sequences used for this study are provided in supplementary tables. High-throughput DNA sequencing data files are available from the National Center for Biotechnology Informationʼs Sequence Read Archive under accession code PRJNA898625 (ref. ^99^). DNA sequences of AAV genomes are provided in the Supplementary Information. Key plasmids from this work are available from Addgene, and other plasmids and raw data are available from the corresponding author upon reasonable request.

## Code availability

The code used for analysis of HTS data is available at https://github.com/pinellolab/CRISPResso2.
