# Supplementary material for: Efficient prime editing in mouse brain, liver and heart with dual AAVs
Source: Nat Biotechnol. 2023 May 4;42(2):253–64. doi: 10.1038/s41587-023-01758-z (PMC10869272; doi:10.1038/s41587-023-01758-z)
Supplement: Supplementary file 1 — Supplementary Figs. 1–6, Supplementary Notes 1–6, Supplementary Sequences and Table of Contents also describing supplementary tables (which are provided in a separate Excel file). [file 41587_2023_1758_MOESM1_ESM.pdf]

nature biotechnology

Article https://doi.org/10.1038/s41587-023-01758-z

Efficient prime editing in mouse brain, liver and heart with dual AAVs

In the format provided by the authors and unedited

Supplementary Information

Supplementary Figure 1. PE-mediated installation of protective *APOE3* R136S Christchurch allele.

Supplementary Figure 2. PE-mediated installation of *Pcsk9* Q155H.

Supplementary Figure 3. Raw (unnormalized) levels of plasma analytes of male and female mice treated with either v3em PE3-AAV9 installing *Pcsk9* Q152H mutation or untreated.

Supplementary Figure 4. Assessment of LDL receptor expression.

Supplementary Figure 5. Raw western blots.

Supplementary Figure 6. FACS gating strategy for brain nuclei.

Supplementary Note 1. Design of intein-split prime editors.

Supplementary Note 2. Efficiency and MMR recognition of a variety of prime edits in cultured cells.

Supplementary Note 3. Reduction of prime editor protein size for packaging in AAV.

Supplementary Note 4. v3em PE-AAV architecture increases in vivo PE expression.

Supplementary Note 5. Inclusion of a nicking sgRNA increases prime editing efficiency in vivo.

Supplementary Note 6. Impact of mouse sex on introduction of *Pcsk9* Q155H with v3em PE3-AAV9 and circulating cholesterol.

Supplementary Sequences. Sequences of AAVs used in this study

Supplementary Tables (provided as separate file):

> **Supplementary Table 1.** Primers used for genomic DNA amplification and their corresponding amplicons.
>
> **Supplementary Table 2.** All pegRNAs, prime editor and AAV architectures used for this study.
>
> **Supplementary Table 3.** All nicking sgRNAs used for this study.
>
> **Supplementary Table 4.** Probes and primers used to quantify viral genomes in liver tissues.
>
> **Supplementary Table 5.** Sequences of *Pcsk9* pegRNA off-target sites identified by CIRCLE-seq and validated by amplicon sequencing in this study.

Supplementary Table 6. Sequences of *Pcsk9* nicking sgRNA off-target sites identified by CIRCLE-seq and validated by amplicon sequencing in this study.

Supplementary Table 7. Circle-seq output for *Pcsk9* Q155H pegRNA.

Supplementary Table 8. Circle-seq output for *Pcsk9* Q155H nicking sgRNA**.**

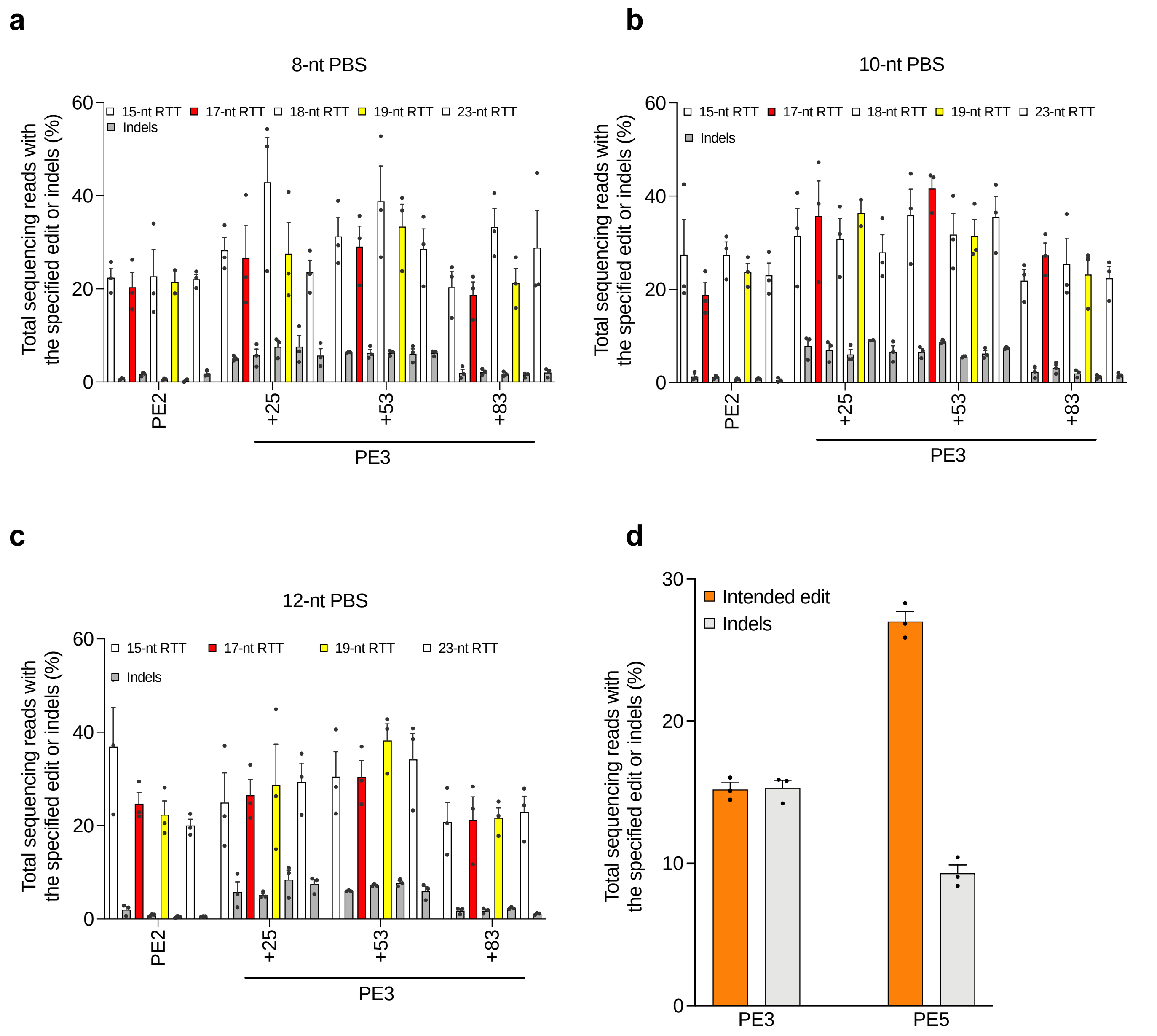

Supplementary Figure 1. PE-mediated installation of protective *APOE3* R136S Christchurch allele. **a-c**, Screening of pegRNAs with various PBS length (8-nt, 10-nt and 12-nt) and RTT (15-nt, 17-nt, 18-nt, 19-nt and 23-nt) along with three nicking guides (+25, +53 and +83) in HEK293T cells. Data are shown as mean±SEM for n=3 biological replicates. **d**, PE3 and PE5 (PE3 + MLH1dn) installation of protective *APOE3* R136S Christchurch allele in immortalized mouse astrocytes. Data are shown as mean±SEM for n=3 biological replicates.

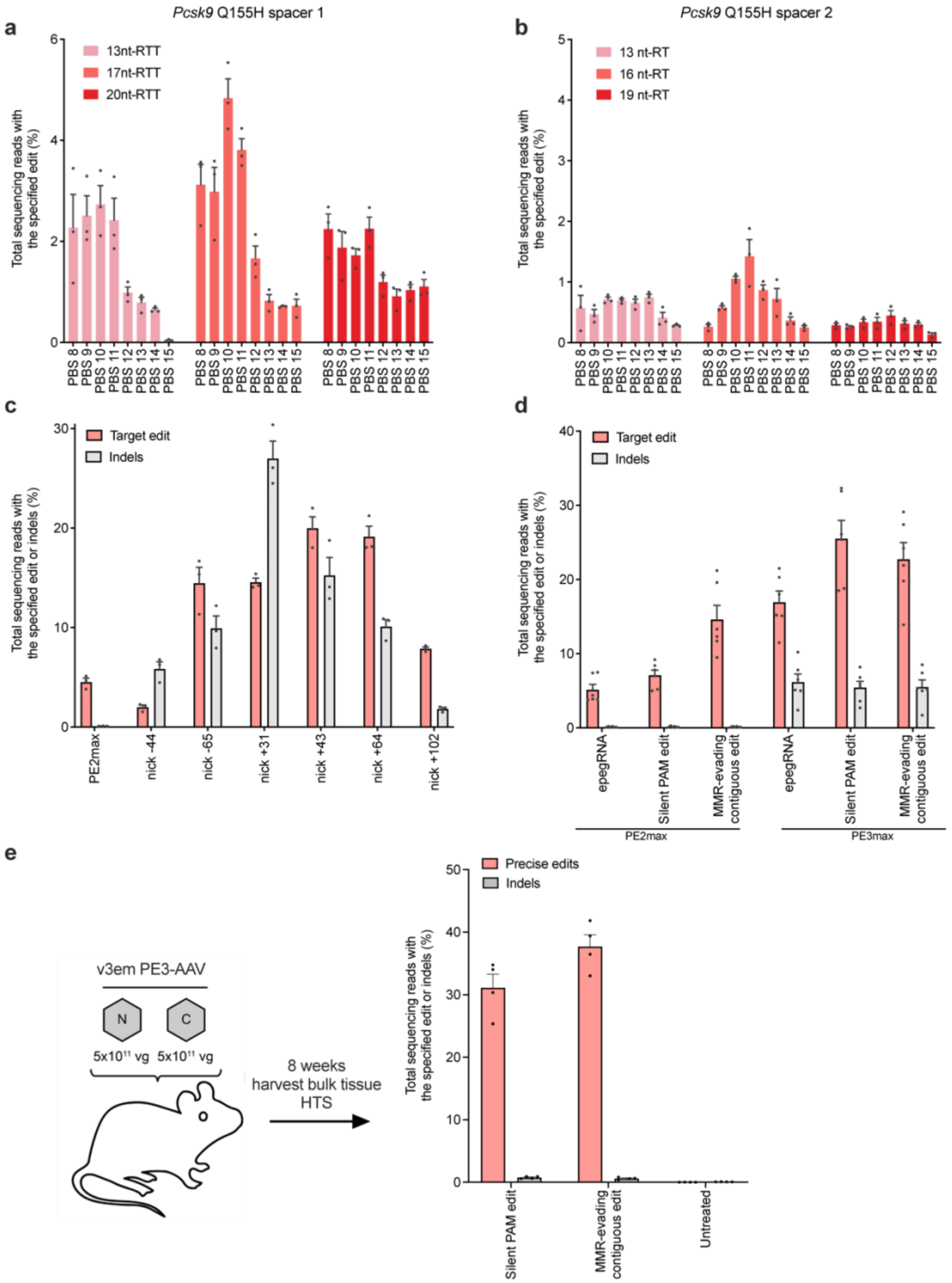

Supplementary Figure 2. PE-mediated installation of *Pcsk9* Q155H. a-**b**, Screening of pegRNAs with various two protospacers, multiple PBS lengths (8-nt, 9-nt, 10-nt, 11-nt, 12-nt, 13-nt, 14-nt and 15-nt) and RTT lengths (13-nt, 17-nt and 20-nt) in Neuro-2a cells. **c**, Screening of nicking guides (-45, -66, +30, +42, +63 and +101) in in Neuro-2a cells. **d**, Further improvements in prime editing efficiencies from using engineered pegRNAs, making a silent PAM edit, and installing silent MMR-evading edits with +64 nicking sgRNA. Data are shown as mean±SEM for n=3 biological replicates. **e**, v3em PE3-AAV9 with epegRNAs encoding PAM-disrupting silent edit or MMR-evading silent edits were injected into 6- to 8-week-old C57BL/6 mice and liver was harvested eight weeks post injection to assess bulk editing. Dots represent individual mice and error bars represent mean±SEM for n=4 mice.

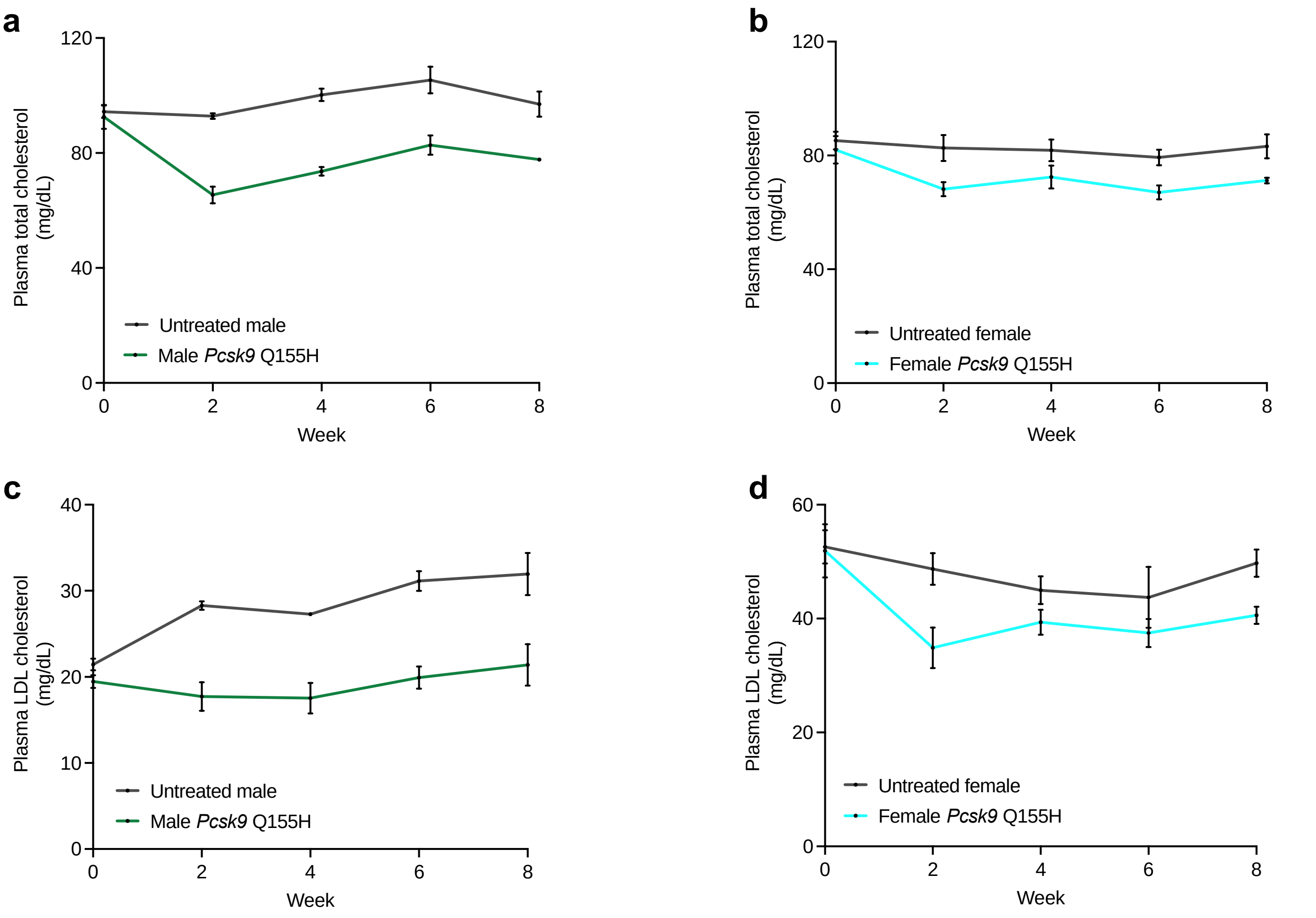

Supplementary Figure 3. Raw (unnormalized) levels of plasma analytes of male and female mice treated with either v3em PE3-AAV9 installing *Pcsk9* Q152H mutation or untreated. a-**b**, Total plasma cholesterol in C57BL/6 male mice **a** and female mice **b**. **c**-**d**, Plasma LDL cholesterol levels from C57BL/6 male mice **c** and female mice **d**. Data are shown as mean±SEM for n=4 mice.

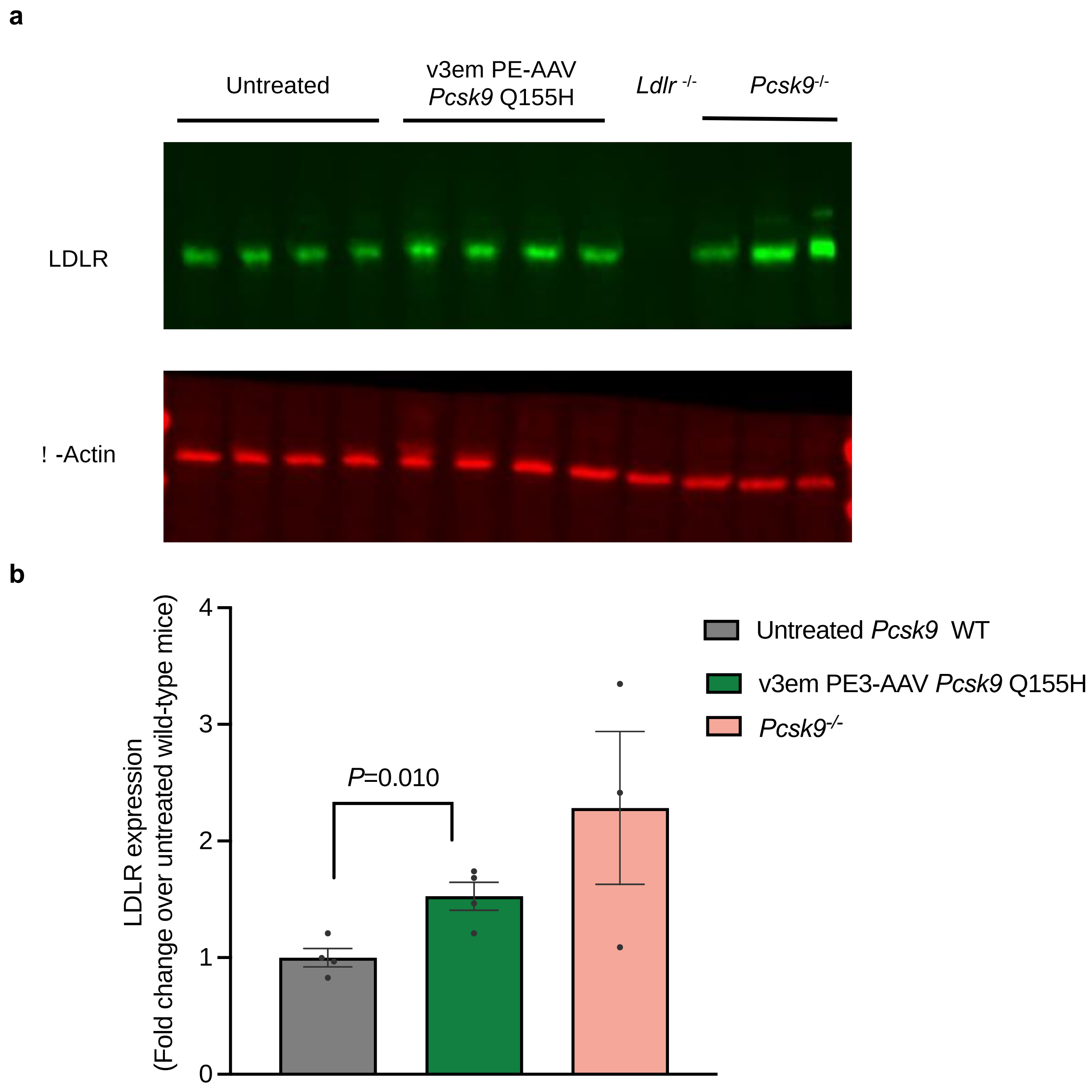

Supplementary Figure 4. Assessment of LDL receptor expression. a, Western blot evaluating LDL receptor expression on liver extracts of untreated, v3em PE-AAV9 *Pcsk9* Q155H treated, *Ldlr*$^{-/-}$ and *Pcsk9*$^{-/-}$ mice. *Ldlr*$^{-/-}$ and *Pcsk9*$^{-/-}$ mice samples were used as negative and positive control, respectively. Raw, uncropped membrane images shown in Supplementary Fig 5. **b**, Densitometry-based quantification of LDL receptor expression from western blots. Data are normalized to untreated and shown as individual data points and mean±SEM for n=3-4 mice. Significance was calculated by two-tailed unpaired t-test.

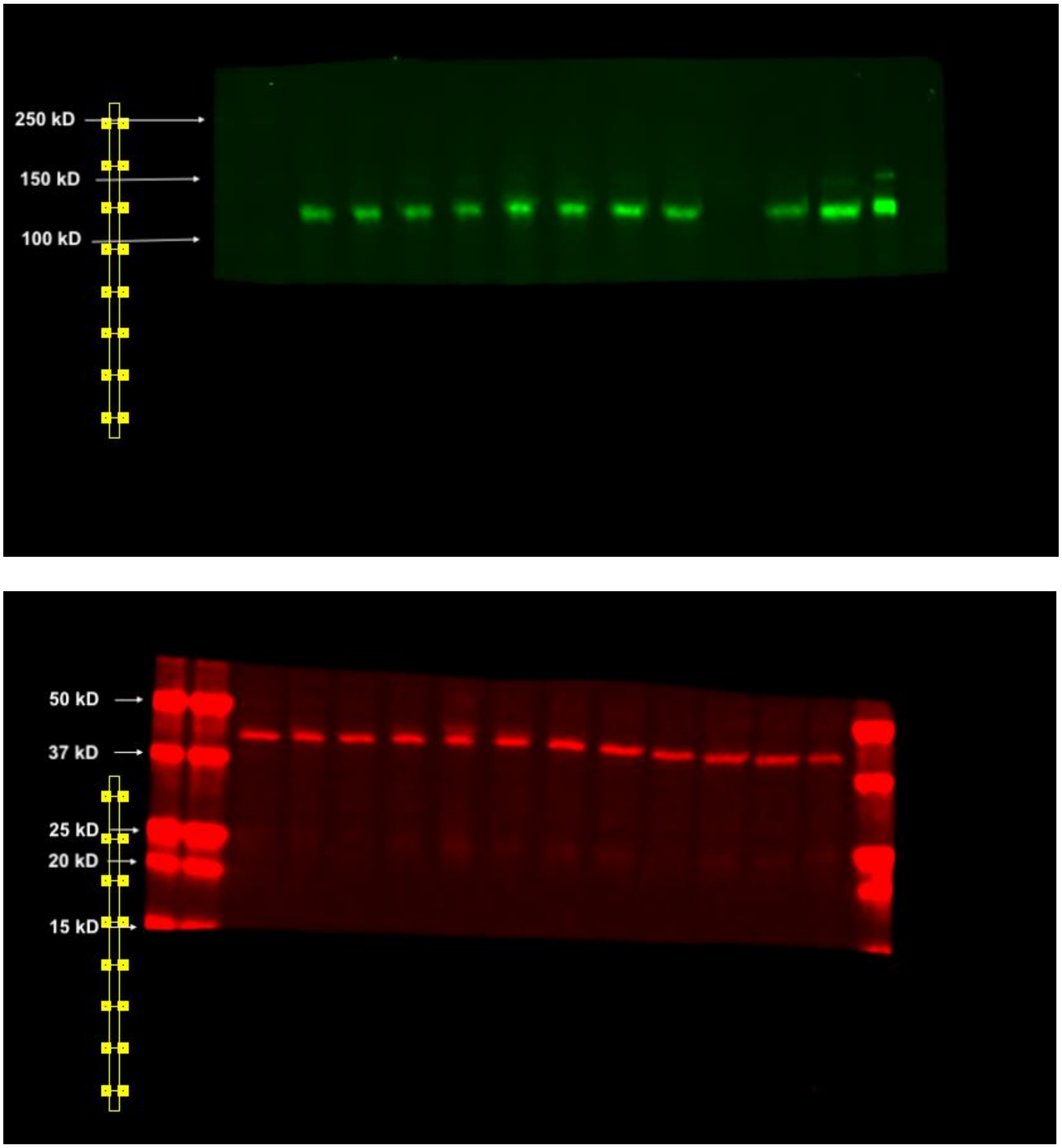

Supplementary Figure 5. Raw western blot images. Uncropped images for LDL receptor (top) and β-Actin (bottom) from liver extracts of v3em PE-AAV9 *Pcsk9* Q155H treated, *Ldlr*$^{-/-}$ and *Pcsk9*$^{-/-}$ mice. *Ldlr*$^{-/-}$ and *Pcsk9*$^{-/-}$ mice samples were used as negative and positive control, respectively. A single gel was transferred to nitrocellulose and was cut horizontally for staining to avoid a bright nonspecific band that could interfere with β-Actin staining then the separated membranes were processed in parallel. Note that the molecular weight ladder and is only faintly visible in the 800 nm channel. Relative expression was normalized to β-Actin loading control on the separated membrane.

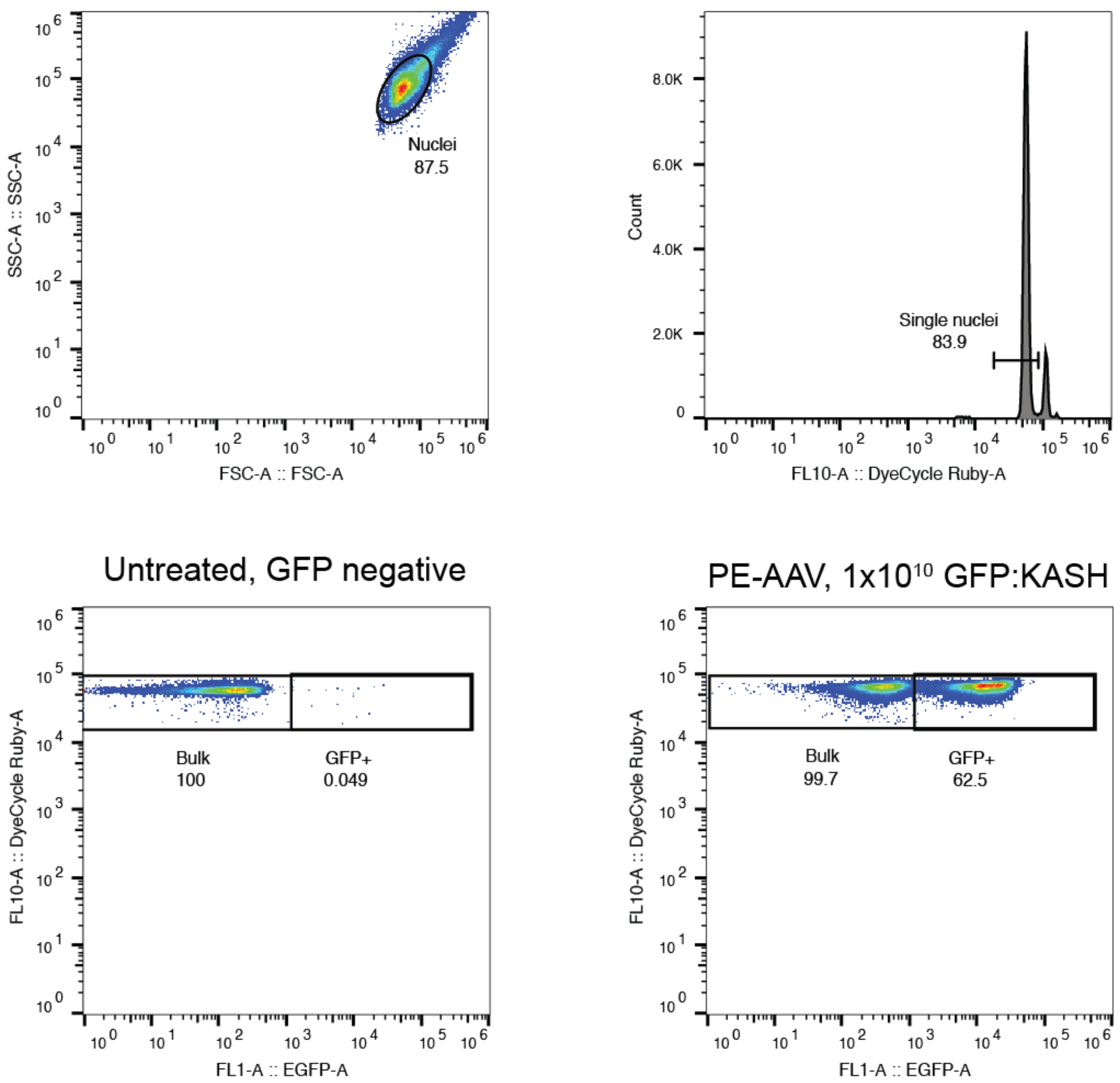

Supplementary Figure 6. FACS gating strategy for brain nuclei. Nuclei were isolated from fresh or previously frozen brain tissue stained with DyeCycle Ruby. Gates were drawn around nuclei based on forward and side scatter area, then gated on singlets based on DyeCycle Ruby intensity, then sorted based on GFP fluorescence into a “bulk” population containing all nuclei regardless of GFP positivity, and a GFP+ population

Supplementary Note 1. Design of intein-split prime editors.

We identified positions within loop regions of SpCas9 that we hypothesized might accommodate fusion to the intein halves with minimal disruption to function based on our analysis of crystal structures containing SpCas9[1], on reports of SpCas9 locations that support circular permutation[2,3], and on the presence of nucleophilic residues that might support the intein splicing mechanism[4]. Prime editing activity of both the 1024 and 844 splits were dependent on splicing competency of the intein (Extended Data Fig. 1), with the catalytically inactive intein 1024-CFN split being on average 39% less active than the catalytically active 1024-CFN intein split, and the catalytically inactive 844-CFN split on average 95% less active than the catalytically active 844-CFN intein split. These data indicate that the inteins function partially as a dimerization domain but that splicing activity enhances editing, in contrast to intein-split base editors that do not require catalytically competent inteins[5]. Because the association of the two halves of Cas9 can be driven by scaffolding of the sgRNA[6], we also assessed the 1024-CFN split without any inteins and found that editing activity was almost completely lost (PE2 1024-CFN Δintein resulted in only 2.6% of the activity of the catalytically competent PE2 1024-CFN intein split) (Extended Data Fig. 1), thus establishing the necessity of the inteins to enable split prime editor association.

Supplementary Note 2. Efficiency and MMR recognition of a variety of prime edits in cultured cells.

To characterize a diversity of prime edits in the same target gene in cultured cells, we screened pegRNAs encoding six new edits (+1 C-to-G; + 1 C-to-G and +5 G-to-T; +2 G-to-C; +1 CTT insertion; +1 CCC insertion; and +1 GCA insertion) at the *Dnmt1* locus in mouse Neuro 2a cells (N2a) cells using PE3 (Extended Data Fig. 2a). Compared to the previously validated +5 G-to-T edit that yielded 7.6% average editing in N2a cells *in vitro*, the six new edits all exhibited higher average editing efficiencies ranging from 14% to 45% (Extended Data Fig. 2a).

To assess whether the observed differences in prime editing efficiency were due to the reversion of prime editing intermediates by MMR, we transfected N2a cells with either PE2 or PE4 and pegRNAs encoding the +5 G-to-T, +1 C-to-G, +1 CCC insertion, or +2 G-to-C edits. The efficiency of +5 G-to-T and +1 C-to-G edits were improved 5.8-fold and 3.2-fold, respectively, with the addition of MLH1dn (PE4), indicating that these edits are impeded by MMR[7] (Fig. 2a, Extended Data Fig. 2b). In contrast, the +2 G-to-C and +1 CCC insertion edits both exhibited comparatively higher editing efficiencies of 20% and 52%, respectively, with PE2,

and the efficiency of these edits was not improved by addition of MLH1dn (Fig. 2a, Extended Data Fig. 2b). These data are consistent with previous observations that prime editing intermediates containing C•C mismatches and multiple contiguous insertions are poor substrates for MMR, allowing the +2 G-to-C and +1 CCC insertion prime editing intermediates to natively evade MMR resulting in higher prime editing efficiencies [8] [7,9].

Supplementary Note 3. Reduction of prime editor protein size for packaging in AAV.

Although the use of prime editors containing smaller Cas variants such as SaCas9 is one potential solution to accommodating PEs with larger promoters in a dual AAV system, we and others have found the activity of SaCas9-derived PEs to be lower overall than SpCas9-based PEs (Extended Data Fig. 5a)[10,11]. We therefore chose to develop a dual-AAV platform for delivery of SpCas9-based PEs, which are more thoroughly characterized and offer increased targeting flexibility and higher editing efficiencies compared with SaCas9 PEs.

Truncations of MMLV RT in the connection domain that separates the largely functionally distinct polymerase and RNaseH subunits have been shown to retain reverse transcriptase activity in PEs[11-14]. We transfected HEK293T cells with RNaseH truncated PE (PE ΔRNaseH). PE ΔRNaseH performed similarly to full-length PE across a variety of edits in HEK293T cells with and without nicking sgRNA (Extended Data Fig. 5b), except for the *HEK3* +1 LoxP insertion edit, where PE2 ΔRNaseH and PE3 ΔRNaseH yielded half the efficiency of full-length PE2 and PE3 editing efficiency, respectively (P=0.010 for PE3 vs PE3 ΔRNaseH; Extended Data Fig. 5b). This result indicates that the RNaseH domain of MMLV RT is not an essential prime editor component in immortalized cells for most tested edits and can be removed to reduce PE size, consistent with other reports using ΔRNaseH PEs *in vitro*[10,11,13].

Supplementary Note 4. v3em PE-AAV architecture increases in vivo PE expression.

To verify that the v3em PE-AAV architecture increases prime editor expression, we directly compared *in vivo* prime editor expression from v1em and v3em PE3-AAV9 architectures. We quantified viral genomes from the N- and C-terminal AAVs using ddPCR with probes specific for SpCas9 amplicons on each half of PEmax and found that the average number of transduced viral genomes did not significantly differ between N- and C-terminal AAV halves or between v1em PE3-AAV and v3em PE3-AAV architectures at either dose by unpaired t-tests with correction for multiple comparisons (Extended Data Fig. 6a). We analyzed

expression of both PE-AAV halves of v1em PE3-AAV and v3em PE3-AAV in bulk liver mRNA by generating cDNA and performing ddPCR quantification of both N- and C-terminal halves, normalized to *Gapdh* expression levels (Extended Data Fig. 6b). When analyzing RNA expression differences, we observed higher expression of both N- and C-terminal PE with v3em PE3-AAV (3.5-fold to 4.8-fold higher than that of v1em), consistent with the editing differences observed between the two architectures (Fig. 4e). These data collectively suggest that prime editor expression is a bottleneck of editing efficiency in the liver that is largely overcome by the v3em PE3-AAV architecture.

Notably, we observed that expression of the N-terminal transcript was consistently lower than that of the C-terminal transcript across architectures and doses. For example, the N-terminal prime editor transcript was 13-fold less abundant than the C-terminal prime editor transcript with the v3em architecture at the 1×10^{12} vg dose (P=0.0033). The consistently lower expression of the N-terminal half indicates that the N-terminal half of the prime editor may be more limiting than the C-terminal transcript in these contexts. To assess whether increasing the ratio of N-terminal PE half would result in additional *in vivo* editing efficiency gains, we delivered v3em PE3-AAV9, encoding the *Dnmt* +2 G to C edit at the same total dose of 1×10^{12} vg but at a ratio of 2.5:1 (7.1×10^{11} vg N-terminal half and 2.9×10^{11} vg C-terminal half). We observed that increasing the ratio of N-terminal PE half to C-terminal PE half did not significantly alter prime editing in the liver, with the 1:1 ratio yielding 46% prime editing and the 2.5:1 ratio yielding 38% (P=0.35), but decreased prime editing efficiency in heart, with the 1:1 ratio yielding 11% prime editing and the 2.5:1 ratio yielding 5.6% (P=0.037). Prime editing in muscle did not change significantly, with the 1:1 ratio yielding 1.1% and the 2.5:1 ratio yielding 0.7% (P=0.61) (Extended Data Fig. 6c). These results indicate that either the N-terminal half is not limiting and expression may be limited at translation rather than transcription, or that reducing the amount of C-terminal transcript outweighs the benefit of increasing the amount of N-terminal transcript. We recommend using a 1:1 ratio of v3em-PE AAVs but note that additional experiments characterizing the expression of both prime editor protein halves could yield insights to further improve *in vivo* prime editing efficiencies.

Supplementary Note 5. Inclusion of a nicking sgRNA increases prime editing efficiency in vivo.

In the context of the optimized v1em PE-AAV architecture, we assessed the importance of including a nicking sgRNA *in vivo* (the PE3 strategy). Inclusion of a nicking sgRNA in the PE3 system can greatly increase prime editing efficiencies in cultured cells by biasing cellular repair

machinery to repair the non-edited strand[7,15,16]. We injected v1em PE2-AAV9 (lacking the nick-inducing sgRNA) or v1em PE3-AAV9 (with the nicking sgRNA) at a total dose of 1×10^{11} vg (5×10^{10} vg per half) with 1×10^{10} vg promoter-matched AAV9 EGFP:KASH by P0 ICV. Three weeks later, we isolated nuclei from neocortex, sorted nuclei by FACS, and analyzed genomic DNA by HTS. The addition of the nicking sgRNA greatly improved prime editing efficiency for both edits (Extended Data Fig. 8), with the *Dnmt1* +1 C-to-G edits increasing in efficiency by 9.8-fold in bulk cortex and 12-fold in the GFP+ population for PE3 compared to PE2. Similarly, inclusion of a nicking sgRNA improved prime editing with the *Dnmt1* +2 G-to-C edit by 2.8-fold in bulk cortex and 2.5-fold in the GFP-positive population. These results demonstrate the importance of including a nicking sgRNA when performing prime editing *in vivo.*

Supplementary Note 6. Impact of mouse sex on circulating cholesterol after introduction of *Pcsk9* Q155H with v3em PE3-AAV9.

We observed sex-dependent differences in editing and circulating cholesterol in response to installation of Pcsk9 Q155H via v3em PE3-AAV9. Editing efficiency in male mice (n=4) was 43.2% and in female mice (n=4) was 35.6% (P=0.037 by unpaired two-tailed t test) (Extended Data Fig. 9a). Sex-dependent differences in AAV transgene expression in liver hepatocytes have been established[17,18] and may explain the observed difference in editing efficiency between male and female mice. In PE-treated male mice, the total plasma cholesterol was lowered by 30% compared to untreated control male mice at two weeks post-injection (P=0.0004 by two-way ANOVA), nearing the degree of total cholesterol-lowering observed in *Pcsk9* knockout mice[19] or in highly efficient base-editing mediated knockdown[20-22] (Extended Data Fig. 9b, Supplementary Fig. 3a). In contrast, the reduction in total cholesterol in female mice was 15% compared to age-matched untreated female mice at two weeks post-injection (Extended Data Fig. 9c, Supplementary Fig. 3b).

The effect on LDL cholesterol was also more prominent in male mice with 38% reduction at two weeks post-injection, persisting over time (Extended Data Fig. 9d,e, Supplementary Fig. 3c,d) (P=0.00035 by two-way ANOVA at eight weeks). Loss-of-function *PCSK9* mutations decrease the rate of LDL receptor (LDLR) degradation in the liver, thereby reducing the level of circulating LDL cholesterol. Reduction of LDL cholesterol levels in male mice were supported by western blot analysis showing increases in LDLR expression in liver tissue of PE treated mice compared to untreated mice (Supplementary Fig. 4, Supplementary Fig. 5) (P=0.010, unpaired t-test). While a slight difference in editing efficiency may partially explain the differences in

circulating cholesterol between male and female mice, similar degrees of sex-dependent differences in lipid response in mice have been previously reported[23-25].

Supplementary Sequences. Sequences of AAVs used in this study.

Sequence of N-term v1em PE3-AAV (5' to 3'), 4915 bp

ITR-EFS promoter-N-term PEmax (start codon-SV40NLS-SpCas9)-NpuN-SV40NLS-W3-bGH polyA-sgRNA (protospacer in bold)-human U6-ITR
(Sequences in grey contain restriction sites for cloning)

CTGCGCGCTCGCTCGCTCACTGAGGCCGCCCGGGCAAAGCCCGGGCGTCGGGCGACCTT
TGGTCGCCCGGCCTCAGTGAGCGAGCGAGCGCGCAGAGAGGGAGTGGCCAACTCCATCA
CTAGGGGTTCCTGCGGCCTCTAGAATTCGCTAGCTAGGTCTTGAAAGGAGTGGGAATTGG
CTCCGGTGCCCGTCAGTGGGCAGAGCGCACATCGCCCACAGTCCCCGAGAAGTTGGGG
GAGGGGTCGGCAATTGATCCGGTGCCTAGAGAAGGTGGCGCGGGGTAAACTGGGAAAGT
GATGTCGTGTACTGGCTCCGCCTTTTTCCCGAGGGTGGGGGAGAACCGTATATAAGTGCA
GTAGTCGCCGTGAACGTTCTTTTTCGCAACGGGTTTGCCGCCAGAACACAGGACCGGTGC
CACCATGAAACGGACAGCCGACGGAAGCGAGTTCGAGTCACCAAAGAAGAAGCGGAAAGT
CGACAAGAAGTACAGCATCGGCCTGGACATCGGCACCAACTCTGTGGGCTGGGCCGTGAT
CACCGACGAGTACAAGGTGCCCAGCAAGAAATTCAAGGTGCTGGGCAACACCGACCGGCA
CAGCATCAAGAAGAACCTGATCGGAGCCCTGCTGTTCGACAGCGGCGAAACAGCCGAGG
CCACCCGGCTGAAGAGAACCGCCAGAAGAAGATACACCAGACGGAAGAACCGGATCTGCT
ATCTGCAAGAGATCTTCAGCAACGAGATGGCCAAGGTGGACGACAGCTTCTTCCACAGACT
GGAAGAGTCCTTCCTGGTGGAAGAGGATAAGAAGCACGAGCGGCACCCCATCTTCGGCAA
CATCGTGGACGAGGTGGCCTACCACGAGAAGTACCCCACCATCTACCACCTGAGAAAGAA
ACTGGTGGACAGCACCGACAAGGCCGACCTGCGGCTGATCTATCTGGCCCTGGCCCACAT
GATCAAGTTCCGGGGCCACTTCCTGATCGAGGGCGACCTGAACCCCGACAACAGCGACGT
GGACAAGCTGTTCATCCAGCTGGTGCAGACCTACAACCAGCTGTTCGAGGAAAACCCCAT
CAACGCCAGCGGCGTGGACGCCAAGGCCATCCTGTCTGCCAGACTGAGCAAGAGCAGAA
AGCTGGAAAATCTGATCGCCCAGCTGCCCGGCGAGAAGAAGAATGGCCTGTTCGGAAACC
TGATTGCCCTGAGCCTGGGCCTGACCCCCAACTTCAAGAGCAACTTCGACCTGGCCGAGG
ATGCCAAACTGCAGCTGAGCAAGGACACCTACGACGACGACCTGGACAACCTGCTGGCCC
AGATCGGCGACCAGTACGCCGACCTGTTTCTGGCCGCCAAGAACCTGTCCGACGCCATCC
TGCTGAGCGACATCCTGAGAGTGAACACCGAGATCACCAAGGCCCCCCTGAGCGCCTCTA
TGATCAAGAGATACGACGAGCACCACCAGGACCTGACCCTGCTGAAAGCTCTCGTGCGGC
AGCAGCTGCCTGAGAAGTACAAAGAGATTTTCTTCGACCAGAGCAAGAACGGCTACGCCG
GCTACATTGACGGCGGAGCCAGCCAGGAAGAGTTCTACAAGTTCATCAAGCCCATCCTGG
AAAAGATGGACGGCACCGAGGAACTGCTCGTGAAGCTGAAGAGAGAGGACCTGCTGCGG
AAGCAGCGGACCTTCGACAACGGCAGCATCCCCCACCAGATCCACCTGGGAGAGCTGCA
CGCCATTCTGCGGCGGCAGGAAGATTTTTACCCATTCCTGAAGGACAACCGGGAAAAGAT
CGAGAAGATCCTGACCTTCCGCATCCCCTACTACGTGGGCCCTCTGGCCAGGGGAAACAG
CAGATTCGCCTGGATGACCAGAAAGAGCGAGGAAACCATCACCCCCTGGAACTTCGAGGA
AGTGGTGGACAAGGGCGCTTCCGCCCAGAGCTTCATCGAGCGGATGACCAACTTCGATAA
GAACCTGCCCAACGAGAAGGTGCTGCCCAAGCACAGCCTGCTGTACGAGTACTTCACCGT
GTATAACGAGCTGACCAAAGTGAAATACGTGACCGAGGGAATGAGAAAGCCCGCCTTCCT
GAGCGGCGAGCAGAAAAAGGCCATCGTGGACCTGCTGTTCAAGACCAACCGGAAAGTGAC
CGTGAAGCAGCTGAAAGAGGACTACTTCAAGAAAATCGAGTGCTTCGACTCCGTGGAAATC
TCCGGCGTGGAAGATCGGTTCAACGCCTCCCTGGGCACATACCACGATCTGCTGAAAATT
ATCAAGGACAAGGACTTCCTGGACAATGAGGAAAACGAGGACATTCTGGAAGATATCGTGC
TGACCCTGACACTGTTTGAGGACAGAGAGATGATCGAGGAACGGCTGAAAACCTATGCCC
ACCTGTTCGACGACAAAGTGATGAAGCAGCTGAAGCGGCGGAGATACACCGGCTGGGGC
AGGCTGAGCCGGAAGCTGATCAACGGCATCCGGGACAAGCAGTCCGGCAAGACAATCCT

GGATTTCCTGAAGTCCGACGGCTTCGCCAACAGAAACTTCATGCAGCTGATCCACGACGA
CAGCCTGACCTTTAAAGAGGACATCCAGAAAGCCCAGGTGTCCGGCCAGGGCGATAGCCT
GCACGAGCACATTGCCAATCTGGCCGGCAGCCCCGCCATTAAGAAGGGCATCCTGCAGAC
AGTGAAGGTGGTGGACGAGCTCGTGAAAGTGATGGGCCGGCACAAGCCCGAGAACATCG
TGATCGAAATGGCCAGAGAGAACCAGACCACCCAGAAGGGACAGAAGAACAGCCGCGAG
AGAATGAAGCGGATCGAAGAGGGCATCAAAGAGCTGGGCAGCCAGATCCTGAAAGAACAC
CCCGTGGAAAACACCCAGCTGCAGAACGAGAAGCTGTACCTGTACTACCTGCAGAATGGG
CGGGATATGTACGTGGACCAGGAACTGGACATCAACCGGCTGTCCGACTACGATGTGGAC
GCTATCGTGCCTCAGAGCTTTCTGAAGGACGACTCCATCGACAACAAGGTGCTGACCAGA
AGCGACAAGAACCGGGGCAAGAGCGACAACGTGCCCTCCGAAGAGGTCGTGAAGAAGAT
GAAGAACTACTGGCGGCAGCTGCTGAACGCCAAGCTGATTACCCAGAGAAAGTTCGACAA
TCTGACCAAGGCCGAGAGAGGCGGCCTGAGCGAACTGGATAAGGCCGGCTTCATCAAGA
GACAGCTGGTGGAAACCCGGCAGATCACAAAGCACGTGGCACAGATCCTGGACTCCCGG
ATGAACACTAAGTACGACGAGAATGACAAGCTGATCCGGGAAGTGAAAGTGATCACCCTGA
AGTCCAAGCTGGTGTCCGATTTCCGGAAGGATTTCCAGTTTTACAAAGTGCGCGAGATCAA
CAACTACCACCACGCCCACGACGCCTACCTGAACGCCGTCGTGGGAACCGCCCTGATCAA
AAAGTACCCTAAGCTGGAAAGCGAGTTCGTGTACGGCGACTACAAGGTGTACGACGTGCG
GAAGATGATCGCCAAGTGCCTGTCCTACGAGACAGAGATCCTGACAGTGGAGTATGGCCT
GCTGCCAATCGGCAAGATCGTGGAGAAGAGGATCGAGTGTACCGTGTACTCTGTGGATAA
CAATGGCAACATCTATACACAGCCCGTGGCACAGTGGCACGATAGGGGAGAGCAGGAGGT
GTTCGAGTATTGCCTGGAGGACGGCAGCCTGATCAGGGCAACCAAGGACCACAAGTTCAT
GACAGTGGATGGCCAGATGCTGCCCATCGACGAGATTTTCGAGCGGGAGCTGGACCTGAT
GAGAGTGGATAACCTGCCTAATTCTGGCGGCTCAAAAAGAACCGCCGACGGCAGCGAATT
CGAGAGTCCCAAGAAGAAGAGGAAAGTCTAAGATCTGATAATCAACCTCTGGATTACAAAA
TTTGTGAAAGATTGACTGGTATTCTTAACTATGTTGCTCCTTTTACGCTATGTGGATACGCT
GCTTTAATGCCTTTGTATCATGCTATTGCTTCCCGTATGGCTTTCATTTTCTCCTCCTTGTAT
AAATCCTGGTTAGTTCTTGCCACGGCGGAACTCATCGCCGCCTGCCTTGCCCGCTGCTGG
ACAGGGGCTCGGCTGTTGGGCACTGACAATTCCGTGGTGCGACTGTGCCTTCTAGTTGCC
AGCCATCTGTTGTTTGCCCCTCCCCCGTGCCTTCCTTGACCCTGGAAGGTGCCACTCCCAC
TGTCCTTTCCTAATAAAATGAGGAAATTGCATCGCATTGTCTGAGTAGGTGTCATTCTATTC
TGGGGGGTGGGGTGGGGCAGGACAGCAAGGGGGAGGATTGGGAAGACAATAGCAGGCA
TGCTGGGGATGCGGTGGGCTCTATGGCTCGAGAAAAAAAGCACCGACTCGGTGCCACTTT
TTCAAGTTGATAACGGACTAGCCTTATTTTAACTTGCTATTTCTAGCTCTAAAAC**CGGCTTTT**
TCGCGCGCGCGGCGGTGTTTCGTCCTTTCCACAAGATATATAAAGCCAAGAAATCGAAATA
CTTTCAAGTTACGGTAAGCATATGATAGTCCATTTTAAAACATAATTTTAAAACTGCAAACTA
CCCAAGAAATTATTACTTTCTACGTCACGTATTTTGTACTAATATCTTTGTGTTTACAGTCAA
ATTAATTCTAATTATCTCTCTAACAGCCTTGTATCGTATATGCAAATATGAAGGAATCATGGG
AAATAGGCCCTCTTCCTGCCCGACCTTGCGGCCGCAGGAACCCCTAGTGATGGAGTTGGC
CACTCCCTCTCTGCGCGCTCGCTCGCTCACTGAGGCCGGGCGACCAAAGGTCGCCCGAC
GCCCGGGCTTTGCCCGGGCGGCCTCAGTGAGCGAGCGAGCGCGCAG

Sequence of C-term v1em PE3-AAV (5' to 3'), 4,880 bp

ITR-EFS promoter- SV40NLS- NpuC-C-term PEmax (SpCas9-RT-SV40NLS)-W3-bGH polyA-epegRNA (protospacer in bold)-human U6-ITR
(Sequences in grey contain restriction sites for cloning)

CTGCGCGCTCGCTCGCTCACTGAGGCCGCCCGGGCAAAGCCCGGGCGTCGGGCGACCTT
TGGTCGCCCGGCCTCAGTGAGCGAGCGAGCGCGCAGAGAGGGAGTGGCCAACTCCATCA
CTAGGGGTTCCTGCGGCCTCTAGAATTCGCTAGCTAGGTCTTGAAAGGAGTGGGAATTGG
CTCCGGTGCCCGTCAGTGGGCAGAGCGCACATCGCCCACAGTCCCCGAGAAGTTGGGGG
GAGGGGTCGGCAATTGATCCGGTGCCTAGAGAAGGTGGCGCGGGGTAAACTGGGAAAGT
GATGTCGTGTACTGGCTCCGCCTTTTTCCCGAGGGTGGGGGAGAACCGTATATAAGTGCA
GTAGTCGCCGTGAACGTTCTTTTTCGCAACGGGTTTGCCGCCAGAACACAGGACCGGTGC
CACCATGAAACGGACAGCCGACGGAAGCGAGTTCGAGTCACCAAAGAAGAAGCGGAAAGT
CATCAAGATTGCTACACGGAAATACCTGGGAAAGCAGAACGTGTACGACATCGGCGTGGA
GCGGGATCACAACTTCGCCCTGAAGAATGGCTTTATCGCCAGCAATTGTTTCAACGAAATC
GGCAAGGCTACCGCCAAGTACTTCTTCTACAGCAACATCATGAACTTTTTCAAGACCGAGA
TTACCCTGGCCAACGGCGAGATCCGGAAGCGGCCTCTGATCGAGACAAACGGCGAAACC
GGGGAGATCGTGTGGGATAAGGGCCGGGATTTTGCCACCGTGCGGAAAGTGCTGAGCAT
GCCCCAAGTGAATATCGTGAAAAAGACCGAGGTGCAGACAGGCGGCTTCAGCAAAGAGTC
TATCCTGCCCAAGAGGAACAGCGATAAGCTGATCGCCAGAAAGAAGGACTGGGACCCTAA
GAAGTACGGCGGCTTCGACAGCCCCACCGTGGCCTATTCTGTGCTGGTGGTGGCCAAAGT
GGAAAAGGGCAAGTCCAAGAAACTGAAGAGTGTGAAAGAGCTGCTGGGGATCACCATCAT
GGAAAGAAGCAGCTTCGAGAAGAATCCCATCGACTTTCTGGAAGCCAAGGGCTACAAAGA
AGTGAAAAAGGACCTGATCATCAAGCTGCCTAAGTACTCCCTGTTCGAGCTGGAAAACGGC
CGGAAGAGAATGCTGGCCTCTGCCGGCGAACTGCAGAAGGGAAACGAACTGGCCCTGCC
CTCCAAATATGTGAACTTCCTGTACCTGGCCAGCCACTATGAGAAGCTGAAGGGCTCCCCC
GAGGATAATGAGCAGAAACAGCTGTTTGTGGAACAGCACAAGCACTACCTGGACGAGATC
ATCGAGCAGATCAGCGAGTTCTCCAAGAGAGTGATCCTGGCCGACGCTAATCTGGACAAA
GTGCTGTCCGCCTACAACAAGCACCGGGATAAGCCCATCAGAGAGCAGGCCGAGAATATC
ATCCACCTGTTTACCCTGACCAATCTGGGAGCCCCTGCCGCCTTCAAGTACTTTGACACCA
CCATCGACCGGAAGAGGTACACCAGCACCAAAGAGGTGCTGGACGCCACCCTGATCCACC
AGAGCATCACCGGCCTGTACGAGACACGGATCGACCTGTCTCAGCTGGGAGGTGACTCCG
GCGGAAGCTCTGGTGGCAGCAAGCGGACCGCCGACGGCTCTGAATTCGAGAGCCCTAAG
AAGAAAAGAAAGGTGAGCGGAGGCTCTAGCGGCGGAAGCACCCTGAACATTGAAGACGA
GTATAGACTGCATGAAACAAGCAAGGAACCCGACGTGTCCCTGGGCTCCACCTGGCTGTC
CGACTTTCCCCAGGCCTGGGCCGAGACAGGAGGAATGGGCCTGGCCGTGCGGCAGGCAC
CCCTGATCATCCCTCTGAAGGCCACCTCTACACCCGTGAGCATCAAGCAGTACCCTATGTC
TCAGGAGGCCAGACTGGGCATCAAGCCTCACATCCAGAGGCTGCTGGACCAGGGCATCCT
GGTGCCATGCCAGAGCCCCTGGAACACACCACTGCTGCCCGTGAAGAAGCCAGGCACCA
ATGACTATAGACCCGTGCAGGATCTGAGAGAGGTGAACAAGAGGGTGGAGGATATCCACC
CCACCGTGCCCAACCCTTACAATCTGCTGTCCGGCCTGCCCCCTTCTCACCAGTGGTATAC
AGTGCTGGACCTGAAGGATGCCTTCTTTTGTCTGAGACTGCACCCTACCAGCCAGCCACTG
TTCGCCTTTGAGTGGAGGGACCCTGAGATGGGCATCTCTGGCCAGCTGACCTGGACACGC
CTGCCTCAGGGCTTCAAGAATAGCCCAACACTGTTTAACGAGGCCCTGCACCGCGACCTG
GCAGATTTCCGGATCCAGCACCCAGATCTGATCCTGCTGCAGTACGTGGACGATCTGCTG
CTGGCCGCCACCAGCGAGCTGGATTGCCAGCAGGGAACACGCGCCCTGCTGCAGACCCT
GGGAAACCTGGGATATAGGGCATCCGCCAAGAAGGCCCAGATCTGTCAGAAGCAGGTGAA
GTACCTGGGCTATCTGCTGAAGGAGGGCCAGAGATGGCTGACAGAGGCCAGGAAGGAGA
CAGTGATGGGCCAGCCAACACCCAAGACCCCAAGACAGCTGAGGGAGTTCCTGGGCAAA

GCAGGATTTTGCAGGCTGTTCATCCCAGGATTCGCAGAGATGGCAGCACCTCTGTACCCA
CTGACCAAGCCGGGCACCCTGTTTAATTGGGGCCCTGACCAGCAGAAGGCCTATCAGGAG
ATCAAGCAGGCCCTGCTGACAGCACCAGCCCTGGGCCTGCCAGACCTGACCAAGCCTTTC
GAGCTGTTTGTGGATGAGAAGCAGGGCTACGCCAAGGGCGTGCTGACCCAGAAGCTGGG
ACCATGGAGACGGCCCGTGGCCTATCTGTCCAAGAAGCTGGACCCAGTGGCAGCAGGAT
GGCCACCATGCCTGAGGATGGTGGCAGCAATCGCCGTGCTGACAAAGGATGCCGGCAAG
CTGACCATGGGACAGCCACTGGTCATCCTGGCACCACACGCAGTGGAGGCCCTGGTGAA
GCAGCCTCCAGATCGCTGGCTGTCTAACGCCCGGATGACACACTACCAGGCCCTGCTGCT
GGACACCGATCGCGTGCAGTTTGGCCCTGTGGTGGCCCTGAATCCAGCCACCCTGCTGCC
TCTGCCAGAGGAGGGCCTGCAGCACAACTGTCTGGACATCCTGGCAGAGGCACACGGAA
CAAGGCCAGACCTGACCGATCAGCCCCTGCCTGACGCCGATCACACATGGTATACCGATG
GAAGCTCCCTGCTGCAGGAGGGCCAGAGGAAGGCAGGAGCAGCAGTGACCACAGAGACA
GAAGTGATCTGGGCCAAGGCCCTGCCAGCAGGCACATCCGCCCAGCGGGCCGAGCTGAT
CGCCCTGACCCAGGCCCTGAAGATGGCCGAGGGCAAGAAGCTGAACGTGTACACAGACT
CCAGATATGCCTTCGCCACCGCACACATCCACGGAGAGATCTACAGGCGCCGGGGCTGG
CTGACCTCTGAGGGCAAGGAGATCAAGAACAAGGATGAGATCCTGGCCCTGCTGAAGGCC
CTGTTTCTGCCCAAGCGGCTGAGCATCATCCACTGTCCTGGACACCAGAAGGGACACTCC
GCCGAGGCAAGGGGCAATCGGATGGCCGACCAGGCCGCCAGAAAGGCTGCTATTACTGA
AACTCCCGACACTTCCACTCTGCTGATTGAAAACTCCTCCCCTTCTGGCGGCTCAAAAAGA
ACCGCCGACGGCAGCGAATTCGAGTCTCCCAAGAAGAAGAGGAAAGTCTAAGATCTGATA
ATCAACCTCTGGATTACAAAATTTGTGAAAGATTGACTGGTATTCTTAACTATGTTGCTCCTT
TTACGCTATGTGGATACGCTGCTTTAATGCCTTTGTATCATGCTATTGCTTCCCGTATGGCT
TTCATTTTCTCCTCCTTGTATAAATCCTGGTTAGTTCTTGCCACGGCGGAACTCATCGCCGC
CTGCCTTGCCCGCTGCTGGACAGGGGCTCGGCTGTTGGGCACTGACAATTCCGTGGTGC
GACTGTGCCTTCTAGTTGCCAGCCATCTGTTGTTTGCCCCTCCCCCGTGCCTTCCTTGACC
CTGGAAGGTGCCACTCCCACTGTCCTTTCCTAATAAAATGAGGAAATTGCATCGCATTGTCT
GAGTAGGTGTCATTCTATTCTGGGGGGTGGGGTGGGGCAGGACAGCAAGGGGGAGGATT
GGGAAGACAATAGCAGGCATGCTGGGGATGCGGTGGGCTCTATGGCTCGAGAAAAAAATT
CTAGTTGGTTTAACGCGTAACTAGATAGAACCGCGGTGTGTTTCTGGAGCTGTTCGGGCTG
GCATCTTGCACCGACTCGGTGCCACTTTTTCAAGTTGATAACGGACTAGCCTTATTTTAACT
TGCTATTTCTAGCTCTAAAAC**GCGCGAACAGCTCCAGCCCGC**GGTGTTTCGTCCTTTCCAC
AAGATATATAAAGCCAAGAAATCGAAATACTTTCAAGTTACGGTAAGCATATGATAGTCCAT
TTTAAAACATAATTTTAAAACTGCAAACTACCCAAGAAATTATTACTTTCTACGTCACGTATTT
TGTACTAATATCTTTGTGTTTACAGTCAAATTAATTCTAATTATCTCTCTAACAGCCTTGTATC
GTATATGCAAATATGAAGGAATCATGGGAAATAGGCCCTCTTCCTGCCCGACCTTGCGGCC
GCAGGAACCCCTAGTGATGGAGTTGGCCACTCCCTCTCTGCGCGCTCGCTCGCTCACTGA
GGCCGGGCGACCAAAGGTCGCCCGACGCCCGGGCTTTGCCCGGGCGGCCTCAGTGAGC
GAGCGAGCGCGCAG

Sequence of N-term v2em PE3-AAV (5' to 3'), 5083 bp

ITR-Cbh promoter-N-term PEmax (start codon-SV40NLS-SpCas9)-NpuN-SV40NLS-W3-bGH polyA-ITR
(Sequences in grey contain restriction sites for cloning)

CTGCGCGCTCGCTCGCTCACTGAGGCCGCCCGGGCAAAGCCCGGGCGTCGGGCGACCTT
TGGTCGCCCGGCCTCAGTGAGCGAGCGAGCGCGCAGAGAGGGAGTGGCCAACTCCATCA
CTAGGGGTTCCTGCGGCCTCTAGATCAGGGTACCCGTTACATAACTTACGGTAAATGGCCC
GCCTGGCTGACCGCCCAACGACCCCCGCCCATTGACGTCAATAGTAACGCCAATAGGGAC
TTTCCATTGACGTCAATGGGTGGAGTATTTACGGTAAACTGCCCACTTGGCAGTACATCAA
GTGTATCATATGCCAAGTACGCCCCCTATTGACGTCAATGACGGTAAATGGCCCGCCTGGC
ATTGTGCCCAGTACATGACCTTATGGGACTTTCCTACTTGGCAGTACATCTACGTATTAGTC
ATCGCTATTACCATGGTCGAGGTGAGCCCCACGTTCTGCTTCACTCTCCCCATCTCCCCCC
CCTCCCCACCCCCAATTTTGTATTTATTTATTTTTTAATTATTTTGTGCAGCGATGGGGGCG
GGGGGGGGGGGGGGCGCGCGCCAGGCGGGGCGGGGCGGGGCGAGGGGCGGGGCG
GGGCGAGGCGGAGAGGTGCGGCGGCAGCCAATCAGAGCGGCGCGCTCCGAAAGTTTCC
TTTTATGGCGAGGCGGCGGCGGCGGCGGCCCTATAAAAAGCGAAGCGCGCGGCGGGCG
GGAGTCGCTGCGCGCTGCCTTCGCCCCGTGCCCCGCTCCGCCGCCGCCTCGCGCCGCC
CGCCCCGGCTCTGACTGACCGCGTTACTCCCACAGGTGAGCGGGCGGGACGGCCCTTCT
CCTCCGGGCTGTAATTAGCTGAGCAAGAGGTAAGGGTTTAAGGGATGGTTGGTTGGTGGG
GTATTAATGTTTAATTACCTGGAGCACCTGCCTGAAATCACTTTTTTTCAGGTTGGACCGGT
GCCACCATGAAACGGACAGCCGACGGAAGCGAGTTCGAGTCACCAAAGAAGAAGCGGAA
AGTCGACAAGAAGTACAGCATCGGCCTGGACATCGGCACCAACTCTGTGGGCTGGGCCGT
GATCACCGACGAGTACAAGGTGCCCAGCAAGAAATTCAAGGTGCTGGGCAACACCGACCG
GCACAGCATCAAGAAGAACCTGATCGGAGCCCTGCTGTTCGACAGCGGCGAAACAGCCGA
GGCCACCCGGCTGAAGAGAACCGCCAGAAGAAGATACACCAGACGGAAGAACCGGATCT
GCTATCTGCAAGAGATCTTCAGCAACGAGATGGCCAAGGTGGACGACAGCTTCTTCCACA
GACTGGAAGAGTCCTTCCTGGTGGAAGAGGATAAGAAGCACGAGCGGCACCCCATCTTCG
GCAACATCGTGGACGAGGTGGCCTACCACGAGAAGTACCCCACCATCTACCACCTGAGAA
AGAAACTGGTGGACAGCACCGACAAGGCCGACCTGCGGCTGATCTATCTGGCCCTGGCC
CACATGATCAAGTTCCGGGGCCACTTCCTGATCGAGGGCGACCTGAACCCCGACAACAGC
GACGTGGACAAGCTGTTCATCCAGCTGGTGCAGACCTACAACCAGCTGTTCGAGGAAAAC
CCCATCAACGCCAGCGGCGTGGACGCCAAGGCCATCCTGTCTGCCAGACTGAGCAAGAG
CAGAAAGCTGGAAAATCTGATCGCCCAGCTGCCCGGCGAGAAGAAGAATGGCCTGTTCGG
AAACCTGATTGCCCTGAGCCTGGGCCTGACCCCCAACTTCAAGAGCAACTTCGACCTGGC
CGAGGATGCCAAACTGCAGCTGAGCAAGGACACCTACGACGACGACCTGGACAACCTGCT
GGCCCAGATCGGCGACCAGTACGCCGACCTGTTTCTGGCCGCCAAGAACCTGTCCGACG
CCATCCTGCTGAGCGACATCCTGAGAGTGAACACCGAGATCACCAAGGCCCCCCTGAGCG
CCTCTATGATCAAGAGATACGACGAGCACCACCAGGACCTGACCCTGCTGAAAGCTCTCG
TGCGGCAGCAGCTGCCTGAGAAGTACAAAGAGATTTTCTTCGACCAGAGCAAGAACGGCT
ACGCCGGCTACATTGACGGCGGAGCCAGCCAGGAAGAGTTCTACAAGTTCATCAAGCCCA
TCCTGGAAAAGATGGACGGCACCGAGGAACTGCTCGTGAAGCTGAAGAGAGAGGACCTG
CTGCGGAAGCAGCGGACCTTCGACAACGGCAGCATCCCCCACCAGATCCACCTGGGAGA
GCTGCACGCCATTCTGCGGCGGCAGGAAGATTTTTACCCATTCCTGAAGGACAACCGGGA
AAAGATCGAGAAGATCCTGACCTTCCGCATCCCCTACTACGTGGGCCCTCTGGCCAGGGG
AAACAGCAGATTCGCCTGGATGACCAGAAAGAGCGAGGAAACCATCACCCCCTGGAACTT
CGAGGAAGTGGTGGACAAGGGCGCTTCCGCCCAGAGCTTCATCGAGCGGATGACCAACTT
CGATAAGAACCTGCCCAACGAGAAGGTGCTGCCCAAGCACAGCCTGCTGTACGAGTACTT
CACCGTGTATAACGAGCTGACCAAAGTGAAATACGTGACCGAGGGAATGAGAAAGCCCGC

CTTCCTGAGCGGCGAGCAGAAAAAGGCCATCGTGGACCTGCTGTTCAAGACCAACCGGAA
AGTGACCGTGAAGCAGCTGAAAGAGGACTACTTCAAGAAAATCGAGTGCTTCGACTCCGT
GGAAATCTCCGGCGTGGAAGATCGGTTCAACGCCTCCCTGGGCACATACCACGATCTGCT
GAAAATTATCAAGGACAAGGACTTCCTGGACAATGAGGAAAACGAGGACATTCTGGAAGAT
ATCGTGCTGACCCTGACACTGTTTGAGGACAGAGAGATGATCGAGGAACGGCTGAAAACC
TATGCCCACCTGTTCGACGACAAAGTGATGAAGCAGCTGAAGCGGCGGAGATACACCGGC
TGGGGCAGGCTGAGCCGGAAGCTGATCAACGGCATCCGGGACAAGCAGTCCGGCAAGAC
AATCCTGGATTTCCTGAAGTCCGACGGCTTCGCCAACAGAAACTTCATGCAGCTGATCCAC
GACGACAGCCTGACCTTTAAAGAGGACATCCAGAAAGCCCAGGTGTCCGGCCAGGGCGAT
AGCCTGCACGAGCACATTGCCAATCTGGCCGGCAGCCCCGCCATTAAGAAGGGCATCCTG
CAGACAGTGAAGGTGGTGGACGAGCTCGTGAAAGTGATGGGCCGGCACAAGCCCGAGAA
CATCGTGATCGAAATGGCCAGAGAGAACCAGACCACCCAGAAGGGACAGAAGAACAGCCG
CGAGAGAATGAAGCGGATCGAAGAGGGCATCAAAGAGCTGGGCAGCCAGATCCTGAAAG
AACACCCCGTGGAAAACACCCAGCTGCAGAACGAGAAGCTGTACCTGTACTACCTGCAGA
ATGGGCGGGATATGTACGTGGACCAGGAACTGGACATCAACCGGCTGTCCGACTACGATG
TGGACGCTATCGTGCCTCAGAGCTTTCTGAAGGACGACTCCATCGACAACAAGGTGCTGA
CCAGAAGCGACAAGAACCGGGGCAAGAGCGACAACGTGCCCTCCGAAGAGGTCGTGAAG
AAGATGAAGAACTACTGGCGGCAGCTGCTGAACGCCAAGCTGATTACCCAGAGAAAGTTC
GACAATCTGACCAAGGCCGAGAGAGGCGGCCTGAGCGAACTGGATAAGGCCGGCTTCAT
CAAGAGACAGCTGGTGGAAACCCGGCAGATCACAAAGCACGTGGCACAGATCCTGGACTC
CCGGATGAACACTAAGTACGACGAGAATGACAAGCTGATCCGGGAAGTGAAAGTGATCAC
CCTGAAGTCCAAGCTGGTGTCCGATTTCCGGAAGGATTTCCAGTTTTACAAAGTGCGCGAG
ATCAACAACTACCACCACGCCCACGACGCCTACCTGAACGCCGTCGTGGGAACCGCCCTG
ATCAAAAAGTACCCTAAGCTGGAAAGCGAGTTCGTGTACGGCGACTACAAGGTGTACGAC
GTGCGGAAGATGATCGCCAAGTGCCTGTCCTACGAGACAGAGATCCTGACAGTGGAGTAT
GGCCTGCTGCCAATCGGCAAGATCGTGGAGAAGAGGATCGAGTGTACCGTGTACTCTGTG
GATAACAATGGCAACATCTATACACAGCCCGTGGCACAGTGGCACGATAGGGGAGAGCAG
GAGGTGTTCGAGTATTGCCTGGAGGACGGCAGCCTGATCAGGGCAACCAAGGACCACAA
GTTCATGACAGTGGATGGCCAGATGCTGCCCATCGACGAGATTTTCGAGCGGGAGCTGGA
CCTGATGAGAGTGGATAACCTGCCTAATTCTGGCGGCTCAAAAAGAACCGCCGACGGCAG
CGAATTCGAGAGTCCCAAGAAGAAGAGGAAAGTCTAAGATCTGATAATCAACCTCTGGATT
ACAAAATTTGTGAAAGATTGACTGGTATTCTTAACTATGTTGCTCCTTTTACGCTATGTGGAT
ACGCTGCTTTAATGCCTTTGTATCATGCTATTGCTTCCCGTATGGCTTTCATTTTCTCCTCCT
TGTATAAATCCTGGTTAGTTCTTGCCACGGCGGAACTCATCGCCGCCTGCCTTGCCCGCTG
CTGGACAGGGGCTCGGCTGTTGGGCACTGACAATTCCGTGGTGCGACTGTGCCTTCTAGT
TGCCAGCCATCTGTTGTTTGCCCCTCCCCCGTGCCTTCCTTGACCCTGGAAGGTGCCACTC
CCACTGTCCTTTCCTAATAAAATGAGGAAATTGCATCGCATTGTCTGAGTAGGTGTCATTCT
ATTCTGGGGGGTGGGGTGGGGCAGGACAGCAAGGGGGAGGATTGGGAAGACAATAGCAG
GCATGCTGGGGATGCGGTGGGCTCTATGGGCGGCCGCAGGAACCCCTAGTGATGGAGTT
GGCCACTCCCTCTCTGCGCGCTCGCTCGCTCACTGAGGCCGGGCGACCAAAGGTCGCCC
GACGCCCGGGCTTTGCCCGGGCGGCCTCAGTGAGCGAGCGAGCGCGCAG

Sequence of C-term v2em PE3-AAV (5' to 3'), 4978 bp

ITR-Cbh promoter-SV40NLS-NpuC-C-term PEmax (SpCas9-RT-SV40NLS)-W3-bGHpolyA-ITR
(Sequences in grey contain restriction sites for cloning)

CTGCGCGCTCGCTCGCTCACTGAGGCCGCCCGGGCAAAGCCCGGGCGTCGGGCGACCTT
TGGTCGCCCGGCCTCAGTGAGCGAGCGAGCGCGCAGAGAGGGAGTGGCCAACTCCATCA
CTAGGGGTTCCTGCGGCCTCTAGATCAGGGTACCCGTTACATAACTTACGGTAAATGGCCC
GCCTGGCTGACCGCCCAACGACCCCCGCCCATTGACGTCAATAGTAACGCCAATAGGGAC
TTTCCATTGACGTCAATGGGTGGAGTATTTACGGTAAACTGCCCACTTGGCAGTACATCAA
GTGTATCATATGCCAAGTACGCCCCCTATTGACGTCAATGACGGTAAATGGCCCGCCTGGC
ATTGTGCCCAGTACATGACCTTATGGGACTTTCCTACTTGGCAGTACATCTACGTATTAGTC
ATCGCTATTACCATGGTCGAGGTGAGCCCCACGTTCTGCTTCACTCTCCCCATCTCCCCCC
CCTCCCCACCCCCAATTTTGTATTTATTTATTTTTTAATTATTTTGTGCAGCGATGGGGGCG
GGGGGGGGGGGGGGCGCGCGCCAGGCGGGGCGGGGCGGGGCGAGGGGCGGGGCG
GGGCGAGGCGGAGAGGTGCGGCGGCAGCCAATCAGAGCGGCGCGCTCCGAAAGTTTCC
TTTTATGGCGAGGCGGCGGCGGCGGCGGCCCTATAAAAAGCGAAGCGCGCGGCGGGCG
GGAGTCGCTGCGCGCTGCCTTCGCCCCGTGCCCCGCTCCGCCGCCGCCTCGCGCCGCC
CGCCCCGGCTCTGACTGACCGCGTTACTCCCACAGGTGAGCGGGCGGGACGGCCCTTCT
CCTCCGGGCTGTAATTAGCTGAGCAAGAGGTAAGGGTTTAAGGGATGGTTGGTTGGTGGG
GTATTAATGTTTAATTACCTGGAGCACCTGCCTGAAATCACTTTTTTTCAGGTTGGACCGGT
GCCACCATGAAACGGACAGCCGACGGAAGCGAGTTCGAGTCACCAAAGAAGAAGCGGAA
AGTCATCAAGATTGCTACACGGAAATACCTGGGAAAGCAGAACGTGTACGACATCGGCGT
GGAGCGGGATCACAACTTCGCCCTGAAGAATGGCTTTATCGCCAGCAATTGTTTCAACGAA
ATCGGCAAGGCTACCGCCAAGTACTTCTTCTACAGCAACATCATGAACTTTTTCAAGACCG
AGATTACCCTGGCCAACGGCGAGATCCGGAAGCGGCCTCTGATCGAGACAAACGGCGAAA
CCGGGGAGATCGTGTGGGATAAGGGCCGGGATTTTGCCACCGTGCGGAAAGTGCTGAGC
ATGCCCCAAGTGAATATCGTGAAAAAGACCGAGGTGCAGACAGGCGGCTTCAGCAAAGAG
TCTATCCTGCCCAAGAGGAACAGCGATAAGCTGATCGCCAGAAAGAAGGACTGGGACCCT
AAGAAGTACGGCGGCTTCGACAGCCCCACCGTGGCCTATTCTGTGCTGGTGGTGGCCAAA
GTGGAAAAGGGCAAGTCCAAGAAACTGAAGAGTGTGAAAGAGCTGCTGGGGATCACCATC
ATGGAAAGAAGCAGCTTCGAGAAGAATCCCATCGACTTTCTGGAAGCCAAGGGCTACAAA
GAAGTGAAAAAGGACCTGATCATCAAGCTGCCTAAGTACTCCCTGTTCGAGCTGGAAAACG
GCCGGAAGAGAATGCTGGCCTCTGCCGGCGAACTGCAGAAGGGAAACGAACTGGCCCTG
CCCTCCAAATATGTGAACTTCCTGTACCTGGCCAGCCACTATGAGAAGCTGAAGGGCTCCC
CCGAGGATAATGAGCAGAAACAGCTGTTTGTGGAACAGCACAAGCACTACCTGGACGAGA
TCATCGAGCAGATCAGCGAGTTCTCCAAGAGAGTGATCCTGGCCGACGCTAATCTGGACA
AAGTGCTGTCCGCCTACAACAAGCACCGGGATAAGCCCATCAGAGAGCAGGCCGAGAATA
TCATCCACCTGTTTACCCTGACCAATCTGGGAGCCCCTGCCGCCTTCAAGTACTTTGACAC
CACCATCGACCGGAAGAGGTACACCAGCACCAAAGAGGTGCTGGACGCCACCCTGATCCA
CCAGAGCATCACCGGCCTGTACGAGACACGGATCGACCTGTCTCAGCTGGGAGGTGACTC
CGGCGGAAGCTCTGGTGGCAGCAAGCGGACCGCCGACGGCTCTGAATTCGAGAGCCCTA
AGAAGAAAAGAAAGGTGAGCGGAGGCTCTAGCGGCGGAAGCACCCTGAACATTGAAGAC
GAGTATAGACTGCATGAAACAAGCAAGGAACCCGACGTGTCCCTGGGCTCCACCTGGCTG
TCCGACTTTCCCCAGGCCTGGGCCGAGACAGGAGGAATGGGCCTGGCCGTGCGGCAGGC
ACCCCTGATCATCCCTCTGAAGGCCACCTCTACACCCGTGAGCATCAAGCAGTACCCTATG
TCTCAGGAGGCCAGACTGGGCATCAAGCCTCACATCCAGAGGCTGCTGGACCAGGGCATC
CTGGTGCCATGCCAGAGCCCCTGGAACACACCACTGCTGCCCGTGAAGAAGCCAGGCAC
CAATGACTATAGACCCGTGCAGGATCTGAGAGAGGTGAACAAGAGGGTGGAGGATATCCA

CCCCACCGTGCCCAACCCTTACAATCTGCTGTCCGGCCTGCCCCCTTCTCACCAGTGGTAT
ACAGTGCTGGACCTGAAGGATGCCTTCTTTTGTCTGAGACTGCACCCTACCAGCCAGCCAC
TGTTCGCCTTTGAGTGGAGGGACCCTGAGATGGGCATCTCTGGCCAGCTGACCTGGACAC
GCCTGCCTCAGGGCTTCAAGAATAGCCCAACACTGTTTAACGAGGCCCTGCACCGCGACC
TGGCAGATTTCCGGATCCAGCACCCAGATCTGATCCTGCTGCAGTACGTGGACGATCTGC
TGCTGGCCGCCACCAGCGAGCTGGATTGCCAGCAGGGAACACGCGCCCTGCTGCAGACC
CTGGGAAACCTGGGATATAGGGCATCCGCCAAGAAGGCCCAGATCTGTCAGAAGCAGGTG
AAGTACCTGGGCTATCTGCTGAAGGAGGGCCAGAGATGGCTGACAGAGGCCAGGAAGGA
GACAGTGATGGGCCAGCCAACACCCAAGACCCCAAGACAGCTGAGGGAGTTCCTGGGCA
AAGCAGGATTTTGCAGGCTGTTCATCCCAGGATTCGCAGAGATGGCAGCACCTCTGTACC
CACTGACCAAGCCGGGCACCCTGTTTAATTGGGGCCCTGACCAGCAGAAGGCCTATCAGG
AGATCAAGCAGGCCCTGCTGACAGCACCAGCCCTGGGCCTGCCAGACCTGACCAAGCCTT
TCGAGCTGTTTGTGGATGAGAAGCAGGGCTACGCCAAGGGCGTGCTGACCCAGAAGCTG
GGACCATGGAGACGGCCCGTGGCCTATCTGTCCAAGAAGCTGGACCCAGTGGCAGCAGG
ATGGCCACCATGCCTGAGGATGGTGGCAGCAATCGCCGTGCTGACAAAGGATGCCGGCA
AGCTGACCATGGGACAGCCACTGGTCATCCTGGCACCACACGCAGTGGAGGCCCTGGTG
AAGCAGCCTCCAGATCGCTGGCTGTCTAACGCCCGGATGACACACTACCAGGCCCTGCTG
CTGGACACCGATCGCGTGCAGTTTGGCCCTGTGGTGGCCCTGAATCCAGCCACCCTGCTG
CCTCTGCCAGAGGAGGGCCTGCAGCACAACTGTCTGGACATCCTGGCAGAGGCACACGG
AACAAGGCCAGACCTGACCGATCAGCCCCTGCCTGACGCCGATCACACATGGTATACCGA
TGGAAGCTCCCTGCTGCAGGAGGGCCAGAGGAAGGCAGGAGCAGCAGTGACCACAGAGA
CAGAAGTGATCTGGGCCAAGGCCCTGCCAGCAGGCACATCCGCCCAGCGGGCCGAGCTG
ATCGCCCTGACCCAGGCCCTGAAGATGGCCGAGGGCAAGAAGCTGAACGTGTACACAGA
CTCCAGATATGCCTTCGCCACCGCACACATCCACGGAGAGATCTACAGGCGCCGGGGCTG
GCTGACCTCTGAGGGCAAGGAGATCAAGAACAAGGATGAGATCCTGGCCCTGCTGAAGGC
CCTGTTTCTGCCCAAGCGGCTGAGCATCATCCACTGTCCTGGACACCAGAAGGGACACTC
CGCCGAGGCAAGGGGCAATCGGATGGCCGACCAGGCCGCCAGAAAGGCTGCTATTACTG
AAACTCCCGACACTTCCACTCTGCTGATTGAAAACTCCTCCCCTTCTGGCGGCTCAAAAAG
AACCGCCGACGGCAGCGAATTCGAGTCTCCCAAGAAGAAGAGGAAAGTCTAAGATCTGAT
AATCAACCTCTGGATTACAAAATTTGTGAAAGATTGACTGGTATTCTTAACTATGTTGCTCCT
TTTACGCTATGTGGATACGCTGCTTTAATGCCTTTGTATCATGCTATTGCTTCCCGTATGGC
TTTCATTTTCTCCTCCTTGTATAAATCCTGGTTAGTTCTTGCCACGGCGGAACTCATCGCCG
CCTGCCTTGCCCGCTGCTGGACAGGGGCTCGGCTGTTGGGCACTGACAATTCCGTGGTG
CGACTGTGCCTTCTAGTTGCCAGCCATCTGTTGTTTGCCCCTCCCCCGTGCCTTCCTTGAC
CCTGGAAGGTGCCACTCCCACTGTCCTTTCCTAATAAAATGAGGAAATTGCATCGCATTGT
CTGAGTAGGTGTCATTCTATTCTGGGGGGTGGGGTGGGGCAGGACAGCAAGGGGGAGGA
TTGGGAAGACAATAGCAGGCATGCTGGGGATGCGGTGGGCTCTATGGGCGGCCGCAGGA
ACCCCTAGTGATGGAGTTGGCCACTCCCTCTCTGCGCGCTCGCTCGCTCACTGAGGCCGG
GCGACCAAAGGTCGCCCGACGCCCGGGCTTTGCCCGGGCGGCCTCAGTGAGCGAGCGA
GCGCGCAG

Sequence of v2em EGFP:KASH pegRNA/sgRNA (5' to 3'), 3438 bp

ITR-Cbh promoter-EGFP:KASH -sgRNA (protospacer in bold)-mouse U6-epegRNA (protospacer in bold)-human U6-ITR
(Sequences in grey contain restriction sites for cloning)

CTGCGCGCTCGCTCGCTCACTGAGGCCGCCCGGGCAAAGCCCGGGCGTCGGGCGACCTT
TGGTCGCCCGGCCTCAGTGAGCGAGCGAGCGCGCAGAGAGGGAGTGGCCAACTCCATCA
CTAGGGGTTCCTGCGGCCTCTAGATCAGGGTACCCGTTACATAACTTACGGTAAATGGCCC
GCCTGGCTGACCGCCCAACGACCCCCGCCCATTGACGTCAATAGTAACGCCAATAGGGAC
TTTCCATTGACGTCAATGGGTGGAGTATTTACGGTAAACTGCCCACTTGGCAGTACATCAA
GTGTATCATATGCCAAGTACGCCCCCTATTGACGTCAATGACGGTAAATGGCCCGCCTGGC
ATTGTGCCCAGTACATGACCTTATGGGACTTTCCTACTTGGCAGTACATCTACGTATTAGTC
ATCGCTATTACCATGGTCGAGGTGAGCCCCACGTTCTGCTTCACTCTCCCCATCTCCCCCC
CCTCCCCACCCCCAATTTTGTATTTATTTATTTTTTAATTATTTTGTGCAGCGATGGGGGCG
GGGGGGGGGGGGGGCGCGCGCCAGGCGGGGCGGGGCGGGGCGAGGGGCGGGGCG
GGGCGAGGCGGAGAGGTGCGGCGGCAGCCAATCAGAGCGGCGCGCTCCGAAAGTTTCC
TTTTATGGCGAGGCGGCGGCGGCGGCGGCCCTATAAAAAGCGAAGCGCGCGGCGGGCG
GGAGTCGCTGCGCGCTGCCTTCGCCCCGTGCCCCGCTCCGCCGCCGCCTCGCGCCGCC
CGCCCCGGCTCTGACTGACCGCGTTACTCCCACAGGTGAGCGGGCGGGACGGCCCTTCT
CCTCCGGGCTGTAATTAGCTGAGCAAGAGGTAAGGGTTTAAGGGATGGTTGGTTGGTGGG
GTATTAATGTTTAATTACCTGGAGCACCTGCCTGAAATCACTTTTTTTCAGGTTGGACCGGT
GCCACCATGGTGAGCAAGGGCGAGGAGCTGTTCACCGGGGTGGTGCCCATCCTGGTCGA
GCTGGACGGCGACGTAAACGGCCACAAGTTCAGCGTGTCCGGCGAGGGCGAGGGCGATG
CCACCTACGGCAAGCTGACCCTGAAGTTCATCTGCACCACCGGCAAGCTGCCCGTGCCCT
GGCCCACCCTCGTGACCACCCTGACCTACGGCGTGCAGTGCTTCAGCCGCTACCCCGAC
CACATGAAGCAGCACGACTTCTTCAAGTCCGCCATGCCCGAAGGCTACGTCCAGGAGCGC
ACCATCTTCTTCAAGGACGACGGCAACTACAAGACCCGCGCCGAGGTGAAGTTCGAGGGC
GACACCCTGGTGAACCGCATCGAGCTGAAGGGCATCGACTTCAAGGAGGACGGCAACATC
CTGGGGCACAAGCTGGAGTACAACTACAACAGCCACAACGTCTATATCATGGCCGACAAG
CAGAAGAACGGCATCAAGGTGAACTTCAAGATCCGCCACAACATCGAGGACGGCAGCGTG
CAGCTCGCCGACCACTACCAGCAGAACACCCCCATCGGCGACGGCCCCGTGCTGCTGCC
CGACAACCACTACCTGAGCACCCAGTCCGCCCTGAGCAAAGACCCCAACGAGAAGCGCGA
TCACATGGTCCTGCTGGAGTTCGTGACCGCCGCCGGGATCACTCTCGGCATGGACGAGCT
GTACAAGTCCGGACTCAGATCTCGAGAGGAGGAGGAGGAGACAGACAGCAGGATGCCCC
ACCTCGACAGCCCCGGCAGCTCCCAGCCGAGACGCTCCTTCCTCTCAAGGGTGATCAGG
GCAGCGCTACCGTTGCAGCTGCTTCTGCTGCTGCTGCTGCTCCTGGCCTGCCTGCTACCT
GCCTCTGAAGATGACTACAGCTGCACCCAGGCCAACAACTTTGCCCGATCCTTCTACCCCA
TGCTGCGGTACACCAACGGGCCACCTCCCACCTAGATCTGATAATCAACCTCTGGATTACA
AAATTTGTGAAAGATTGACTGGTATTCTTAACTATGTTGCTCCTTTTACGCTATGTGGATACG
CTGCTTTAATGCCTTTGTATCATGCTATTGCTTCCCGTATGGCTTTCATTTTCTCCTCCTTGT
ATAAATCCTGGTTAGTTCTTGCCACGGCGGAACTCATCGCCGCCTGCCTTGCCCGCTGCT
GGACAGGGGCTCGGCTGTTGGGCACTGACAATTCCGTGGTGCGACTGTGCCTTCTAGTTG
CCAGCCATCTGTTGTTTGCCCCTCCCCCGTGCCTTCCTTGACCCTGGAAGGTGCCACTCC
CACTGTCCTTTCCTAATAAAATGAGGAAATTGCATCGCATTGTCTGAGTAGGTGTCATTCTA
TTCTGGGGGGTGGGGTGGGGCAGGACAGCAAGGGGGAGGATTGGGAAGACAATAGCAG
GCATGCTGGGGATGCGGTGGGCTCTATGGAAGCTTGCAAAAAAAGCACCGACTCGGTGCC
ACTTTTTCAAGTTGATAACGGACTAGCCTTATTTTAACTTGCTATTTCTAGCTCTAAAAC**CGG**
CTTTTTCGCGCGCGCGGCAAACAAGGCTTTTCTCCAAGGGATATTTATAGTCTCAAAACAC
ACAATTACTTTACAGTTAGGGTGAGTTTCCTTTTGTGCTGTTTTTTAAAATAATAATTTAGTAT

TTGTATCTCTTATAGAAATCCAAGCCTATCATGTAAAATGTAGCTAGTATTAAAAAGAACAGA
TTATCTGTCTTTTATCGCACATTAAGCCTCTATAGTTACTAGGAAATATTATATGCAAATTAA
CCGGGGCAGGGGAGTAGCCGAGCTTCTCCCACAAGTCTGTGCGAGGGGGCCGGCGCGG
GCCTAGAGATGGCGGCGTCGGATCGCGAATTCAAAAAAATTCTAGTTGGTTTAACGCGTAA
CTAGATAGAACCGCGGTGTGTTTCTGGAGCTGTTCGGGCTGGCATCTTGCACCGACTCGG
TGCCACTTTTTCAAGTTGATAACGGACTAGCCTTATTTTAACTTGCTATTTCTAGCTCTAAAA
C**GCGCGAACAGCTCCAGCCCGC**GGTGTTTCGTCCTTTCCACAAGATATATAAAGCCAAGA
AATCGAAATACTTTCAAGTTACGGTAAGCATATGATAGTCCATTTTAAAACATAATTTTAAAA
CTGCAAACTACCCAAGAAATTATTACTTTCTACGTCACGTATTTTGTACTAATATCTTTGTGT
TTACAGTCAAATTAATTCTAATTATCTCTCTAACAGCCTTGTATCGTATATGCAAATATGAAG
GAATCATGGGAAATAGGCCCTCTTCCTGCCCGACCTTGCGGCCGCAGGAACCCCTAGTGA
TGGAGTTGGCCACTCCCTCTCTGCGCGCTCGCTCGCTCACTGAGGCCGGGCGACCAAAG
GTCGCCCGACGCCCGGGCTTTGCCCGGGCGGCCTCAGTGAGCGAGCGAGCGCGCAG

Sequence of N-term v3em PE3-AAV (5' to 3'), 4,740 bp

ITR-Cbh promoter-N-term PEmax (start codon-SV40NLS-SpCas9)-NpuN-SV40NLS-SV40 late polyA-ITR
(Sequences in grey contain restriction sites for cloning)

CTGCGCGCTCGCTCGCTCACTGAGGCCGCCCGGGCAAAGCCCGGGCGTCGGGCGACCTT
TGGTCGCCCGGCCTCAGTGAGCGAGCGAGCGCGCAGAGAGGGAGTGGCCAACTCCATCA
CTAGGGGTTCCTGCGGCCTCTAGATCAGGGTACCCGTTACATAACTTACGGTAAATGGCCC
GCCTGGCTGACCGCCCAACGACCCCCGCCCATTGACGTCAATAGTAACGCCAATAGGGAC
TTTCCATTGACGTCAATGGGTGGAGTATTTACGGTAAACTGCCCACTTGGCAGTACATCAA
GTGTATCATATGCCAAGTACGCCCCCTATTGACGTCAATGACGGTAAATGGCCCGCCTGGC
ATTGTGCCCAGTACATGACCTTATGGGACTTTCCTACTTGGCAGTACATCTACGTATTAGTC
ATCGCTATTACCATGGTCGAGGTGAGCCCCACGTTCTGCTTCACTCTCCCCATCTCCCCCC
CCTCCCCACCCCCAATTTTGTATTTATTTATTTTTTAATTATTTTGTGCAGCGATGGGGGCG
GGGGGGGGGGGGGGCGCGCGCCAGGCGGGGCGGGGCGGGGCGAGGGGCGGGGCG
GGGCGAGGCGGAGAGGTGCGGCGGCAGCCAATCAGAGCGGCGCGCTCCGAAAGTTTCC
TTTTATGGCGAGGCGGCGGCGGCGGCGGCCCTATAAAAAGCGAAGCGCGCGGCGGGCG
GGAGTCGCTGCGCGCTGCCTTCGCCCCGTGCCCCGCTCCGCCGCCGCCTCGCGCCGCC
CGCCCCGGCTCTGACTGACCGCGTTACTCCCACAGGTGAGCGGGCGGGACGGCCCTTCT
CCTCCGGGCTGTAATTAGCTGAGCAAGAGGTAAGGGTTTAAGGGATGGTTGGTTGGTGGG
GTATTAATGTTTAATTACCTGGAGCACCTGCCTGAAATCACTTTTTTTCAGGTTGGACCGGT
GCCACCATGAAACGGACAGCCGACGGAAGCGAGTTCGAGTCACCAAAGAAGAAGCGGAA
AGTCGACAAGAAGTACAGCATCGGCCTGGACATCGGCACCAACTCTGTGGGCTGGGCCGT
GATCACCGACGAGTACAAGGTGCCCAGCAAGAAATTCAAGGTGCTGGGCAACACCGACCG
GCACAGCATCAAGAAGAACCTGATCGGAGCCCTGCTGTTCGACAGCGGCGAAACAGCCGA
GGCCACCCGGCTGAAGAGAACCGCCAGAAGAAGATACACCAGACGGAAGAACCGGATCT
GCTATCTGCAAGAGATCTTCAGCAACGAGATGGCCAAGGTGGACGACAGCTTCTTCCACA
GACTGGAAGAGTCCTTCCTGGTGGAAGAGGATAAGAAGCACGAGCGGCACCCCATCTTCG
GCAACATCGTGGACGAGGTGGCCTACCACGAGAAGTACCCCACCATCTACCACCTGAGAA
AGAAACTGGTGGACAGCACCGACAAGGCCGACCTGCGGCTGATCTATCTGGCCCTGGCC
CACATGATCAAGTTCCGGGGCCACTTCCTGATCGAGGGCGACCTGAACCCCGACAACAGC
GACGTGGACAAGCTGTTCATCCAGCTGGTGCAGACCTACAACCAGCTGTTCGAGGAAAAC
CCCATCAACGCCAGCGGCGTGGACGCCAAGGCCATCCTGTCTGCCAGACTGAGCAAGAG
CAGAAAGCTGGAAAATCTGATCGCCCAGCTGCCCGGCGAGAAGAAGAATGGCCTGTTCGG
AAACCTGATTGCCCTGAGCCTGGGCCTGACCCCCAACTTCAAGAGCAACTTCGACCTGGC
CGAGGATGCCAAACTGCAGCTGAGCAAGGACACCTACGACGACGACCTGGACAACCTGCT
GGCCCAGATCGGCGACCAGTACGCCGACCTGTTTCTGGCCGCCAAGAACCTGTCCGACG
CCATCCTGCTGAGCGACATCCTGAGAGTGAACACCGAGATCACCAAGGCCCCCCTGAGCG
CCTCTATGATCAAGAGATACGACGAGCACCACCAGGACCTGACCCTGCTGAAAGCTCTCG
TGCGGCAGCAGCTGCCTGAGAAGTACAAAGAGATTTTCTTCGACCAGAGCAAGAACGGCT
ACGCCGGCTACATTGACGGCGGAGCCAGCCAGGAAGAGTTCTACAAGTTCATCAAGCCCA
TCCTGGAAAAGATGGACGGCACCGAGGAACTGCTCGTGAAGCTGAAGAGAGAGGACCTG
CTGCGGAAGCAGCGGACCTTCGACAACGGCAGCATCCCCCACCAGATCCACCTGGGAGA
GCTGCACGCCATTCTGCGGCGGCAGGAAGATTTTTACCCATTCCTGAAGGACAACCGGGA
AAAGATCGAGAAGATCCTGACCTTCCGCATCCCCTACTACGTGGGCCCTCTGGCCAGGGG
AAACAGCAGATTCGCCTGGATGACCAGAAAGAGCGAGGAAACCATCACCCCCTGGAACTT
CGAGGAAGTGGTGGACAAGGGCGCTTCCGCCCAGAGCTTCATCGAGCGGATGACCAACTT
CGATAAGAACCTGCCCAACGAGAAGGTGCTGCCCAAGCACAGCCTGCTGTACGAGTACTT
CACCGTGTATAACGAGCTGACCAAAGTGAAATACGTGACCGAGGGAATGAGAAAGCCCGC

CTTCCTGAGCGGCGAGCAGAAAAAGGCCATCGTGGACCTGCTGTTCAAGACCAACCGGAA
AGTGACCGTGAAGCAGCTGAAAGAGGACTACTTCAAGAAAATCGAGTGCTTCGACTCCGT
GGAAATCTCCGGCGTGGAAGATCGGTTCAACGCCTCCCTGGGCACATACCACGATCTGCT
GAAAATTATCAAGGACAAGGACTTCCTGGACAATGAGGAAAACGAGGACATTCTGGAAGAT
ATCGTGCTGACCCTGACACTGTTTGAGGACAGAGAGATGATCGAGGAACGGCTGAAAACC
TATGCCCACCTGTTCGACGACAAAGTGATGAAGCAGCTGAAGCGGCGGAGATACACCGGC
TGGGGCAGGCTGAGCCGGAAGCTGATCAACGGCATCCGGGACAAGCAGTCCGGCAAGAC
AATCCTGGATTTCCTGAAGTCCGACGGCTTCGCCAACAGAAACTTCATGCAGCTGATCCAC
GACGACAGCCTGACCTTTAAAGAGGACATCCAGAAAGCCCAGGTGTCCGGCCAGGGCGAT
AGCCTGCACGAGCACATTGCCAATCTGGCCGGCAGCCCCGCCATTAAGAAGGGCATCCTG
CAGACAGTGAAGGTGGTGGACGAGCTCGTGAAAGTGATGGGCCGGCACAAGCCCGAGAA
CATCGTGATCGAAATGGCCAGAGAGAACCAGACCACCCAGAAGGGACAGAAGAACAGCCG
CGAGAGAATGAAGCGGATCGAAGAGGGCATCAAAGAGCTGGGCAGCCAGATCCTGAAAG
AACACCCCGTGGAAAACACCCAGCTGCAGAACGAGAAGCTGTACCTGTACTACCTGCAGA
ATGGGCGGGATATGTACGTGGACCAGGAACTGGACATCAACCGGCTGTCCGACTACGATG
TGGACGCTATCGTGCCTCAGAGCTTTCTGAAGGACGACTCCATCGACAACAAGGTGCTGA
CCAGAAGCGACAAGAACCGGGGCAAGAGCGACAACGTGCCCTCCGAAGAGGTCGTGAAG
AAGATGAAGAACTACTGGCGGCAGCTGCTGAACGCCAAGCTGATTACCCAGAGAAAGTTC
GACAATCTGACCAAGGCCGAGAGAGGCGGCCTGAGCGAACTGGATAAGGCCGGCTTCAT
CAAGAGACAGCTGGTGGAAACCCGGCAGATCACAAAGCACGTGGCACAGATCCTGGACTC
CCGGATGAACACTAAGTACGACGAGAATGACAAGCTGATCCGGGAAGTGAAAGTGATCAC
CCTGAAGTCCAAGCTGGTGTCCGATTTCCGGAAGGATTTCCAGTTTTACAAAGTGCGCGAG
ATCAACAACTACCACCACGCCCACGACGCCTACCTGAACGCCGTCGTGGGAACCGCCCTG
ATCAAAAAGTACCCTAAGCTGGAAAGCGAGTTCGTGTACGGCGACTACAAGGTGTACGAC
GTGCGGAAGATGATCGCCAAGTGCCTGTCCTACGAGACAGAGATCCTGACAGTGGAGTAT
GGCCTGCTGCCAATCGGCAAGATCGTGGAGAAGAGGATCGAGTGTACCGTGTACTCTGTG
GATAACAATGGCAACATCTATACACAGCCCGTGGCACAGTGGCACGATAGGGGAGAGCAG
GAGGTGTTCGAGTATTGCCTGGAGGACGGCAGCCTGATCAGGGCAACCAAGGACCACAA
GTTCATGACAGTGGATGGCCAGATGCTGCCCATCGACGAGATTTTCGAGCGGGAGCTGGA
CCTGATGAGAGTGGATAACCTGCCTAATTCTGGCGGCTCAAAAAGAACCGCCGACGGCAG
CGAATTCGAGTCTCCCAAGAAGAAGAGGAAAGTCTAAGTCGACCTTTATTTGTGAAATTTGT
GATGCTATTGCTTTATTTGTAACCATTATAAGCTGCAATAAACAAGTTAACAACAACAATTGC
ATTCATTTTATGTTTCAGGTTCAGGGGGAGATGTGGAGGTTTTTTAAAGCGCGGCCGCAG
GAACCCCTAGTGATGGAGTTGGCCACTCCCTCTCTGCGCGCTCGCTCGCTCACTGAGGCC
GGGCGACCAAAGGTCGCCCGACGCCCGGGCTTTGCCCGGGCGGCCTCAGTGAGCGAGC
GAGCGCGCAG

Sequence of C-term v3em PE3-AAV (5' to 3'), 4,968 bp

ITR-Cbh promoter-SV40NLS-NpuC-C-term PEmax (SpCas9-RT-SV40NLS)-SV40 late polyA-sgRNRNA (protospacer in bold)-mouse U6-epegRNA (protospacer in bold)-human U6-ITR

(Sequences in grey contain restriction sites for cloning)

CTGCGCGCTCGCTCGCTCACTGAGGCCGCCCGGGCAAAGCCCGGGCGTCGGGCGACCTT
TGGTCGCCCGGCCTCAGTGAGCGAGCGAGCGCGCAGAGAGGGAGTGGCCAACTCCATCA
CTAGGGGTTCCTGCGGCCTCTAGATCAGGGTACCCGTTACATAACTTACGGTAAATGGCCC
GCCTGGCTGACCGCCCAACGACCCCCGCCCATTGACGTCAATAGTAACGCCAATAGGGAC
TTTCCATTGACGTCAATGGGTGGAGTATTTACGGTAAACTGCCCACTTGGCAGTACATCAA
GTGTATCATATGCCAAGTACGCCCCCTATTGACGTCAATGACGGTAAATGGCCCGCCTGGC
ATTGTGCCCAGTACATGACCTTATGGGACTTTCCTACTTGGCAGTACATCTACGTATTAGTC
ATCGCTATTACCATGGTCGAGGTGAGCCCCACGTTCTGCTTCACTCTCCCCATCTCCCCCC
CCTCCCCACCCCCAATTTTGTATTTATTTATTTTTTAATTATTTTGTGCAGCGATGGGGGCG
GGGGGGGGGGGGGGCGCGCGCCAGGCGGGGCGGGGCGGGGCGAGGGGCGGGGCG
GGGCGAGGCGGAGAGGTGCGGCGGCAGCCAATCAGAGCGGCGCGCTCCGAAAGTTTCC
TTTTATGGCGAGGCGGCGGCGGCGGCGGCCCTATAAAAAGCGAAGCGCGCGGCGGGCG
GGAGTCGCTGCGCGCTGCCTTCGCCCCGTGCCCCGCTCCGCCGCCGCCTCGCGCCGCC
CGCCCCGGCTCTGACTGACCGCGTTACTCCCACAGGTGAGCGGGCGGGACGGCCCTTCT
CCTCCGGGCTGTAATTAGCTGAGCAAGAGGTAAGGGTTTAAGGGATGGTTGGTTGGTGGG
GTATTAATGTTTAATTACCTGGAGCACCTGCCTGAAATCACTTTTTTTCAGGTTGGACCGGT
GCCACCATGAAACGGACAGCCGACGGAAGCGAGTTCGAGTCACCAAAGAAGAAGCGGAA
AGTCATCAAGATTGCTACACGGAAATACCTGGGAAAGCAGAACGTGTACGACATCGGCGT
GGAGCGGGATCACAACTTCGCCCTGAAGAATGGCTTTATCGCCAGCAATTGTTTCAACGAA
ATCGGCAAGGCTACCGCCAAGTACTTCTTCTACAGCAACATCATGAACTTTTTCAAGACCG
AGATTACCCTGGCCAACGGCGAGATCCGGAAGCGGCCTCTGATCGAGACAAACGGCGAAA
CCGGGGAGATCGTGTGGGATAAGGGCCGGGATTTTGCCACCGTGCGGAAAGTGCTGAGC
ATGCCCCAAGTGAATATCGTGAAAAAGACCGAGGTGCAGACAGGCGGCTTCAGCAAAGAG
TCTATCCTGCCCAAGAGGAACAGCGATAAGCTGATCGCCAGAAAGAAGGACTGGGACCCT
AAGAAGTACGGCGGCTTCGACAGCCCCACCGTGGCCTATTCTGTGCTGGTGGTGGCCAAA
GTGGAAAAGGGCAAGTCCAAGAAACTGAAGAGTGTGAAAGAGCTGCTGGGGATCACCATC
ATGGAAAGAAGCAGCTTCGAGAAGAATCCCATCGACTTTCTGGAAGCCAAGGGCTACAAA
GAAGTGAAAAAGGACCTGATCATCAAGCTGCCTAAGTACTCCCTGTTCGAGCTGGAAAACG
GCCGGAAGAGAATGCTGGCCTCTGCCGGCGAACTGCAGAAGGGAAACGAACTGGCCCTG
CCCTCCAAATATGTGAACTTCCTGTACCTGGCCAGCCACTATGAGAAGCTGAAGGGCTCCC
CCGAGGATAATGAGCAGAAACAGCTGTTTGTGGAACAGCACAAGCACTACCTGGACGAGA
TCATCGAGCAGATCAGCGAGTTCTCCAAGAGAGTGATCCTGGCCGACGCTAATCTGGACA
AAGTGCTGTCCGCCTACAACAAGCACCGGGATAAGCCCATCAGAGAGCAGGCCGAGAATA
TCATCCACCTGTTTACCCTGACCAATCTGGGAGCCCCTGCCGCCTTCAAGTACTTTGACAC
CACCATCGACCGGAAGAGGTACACCAGCACCAAAGAGGTGCTGGACGCCACCCTGATCCA
CCAGAGCATCACCGGCCTGTACGAGACACGGATCGACCTGTCTCAGCTGGGAGGTGACTC
CGGCGGAAGCTCTGGTGGCAGCAAGCGGACCGCCGACGGCTCTGAATTCGAGAGCCCTA
AGAAGAAAAGAAAGGTGAGCGGAGGCTCTAGCGGCGGAAGCACCCTGAACATTGAAGAC
GAGTATAGACTGCATGAAACAAGCAAGGAACCCGACGTGTCCCTGGGCTCCACCTGGCTG
TCCGACTTTCCCCAGGCCTGGGCCGAGACAGGAGGAATGGGCCTGGCCGTGCGGCAGGC
ACCCCTGATCATCCCTCTGAAGGCCACCTCTACACCCGTGAGCATCAAGCAGTACCCTATG
TCTCAGGAGGCCAGACTGGGCATCAAGCCTCACATCCAGAGGCTGCTGGACCAGGGCATC

CTGGTGCCATGCCAGAGCCCCTGGAACACACCACTGCTGCCCGTGAAGAAGCCAGGCAC
CAATGACTATAGACCCGTGCAGGATCTGAGAGAGGTGAACAAGAGGGTGGAGGATATCCA
CCCCACCGTGCCCAACCCTTACAATCTGCTGTCCGGCCTGCCCCCTTCTCACCAGTGGTAT
ACAGTGCTGGACCTGAAGGATGCCTTCTTTTGTCTGAGACTGCACCCTACCAGCCAGCCAC
TGTTCGCCTTTGAGTGGAGGGACCCTGAGATGGGCATCTCTGGCCAGCTGACCTGGACAC
GCCTGCCTCAGGGCTTCAAGAATAGCCCAACACTGTTTAACGAGGCCCTGCACCGCGACC
TGGCAGATTTCCGGATCCAGCACCCAGATCTGATCCTGCTGCAGTACGTGGACGATCTGC
TGCTGGCCGCCACCAGCGAGCTGGATTGCCAGCAGGGAACACGCGCCCTGCTGCAGACC
CTGGGAAACCTGGGATATAGGGCATCCGCCAAGAAGGCCCAGATCTGTCAGAAGCAGGTG
AAGTACCTGGGCTATCTGCTGAAGGAGGGCCAGAGATGGCTGACAGAGGCCAGGAAGGA
GACAGTGATGGGCCAGCCAACACCCAAGACCCCAAGACAGCTGAGGGAGTTCCTGGGCA
AAGCAGGATTTTGCAGGCTGTTCATCCCAGGATTCGCAGAGATGGCAGCACCTCTGTACC
CACTGACCAAGCCGGGCACCCTGTTTAATTGGGGCCCTGACCAGCAGAAGGCCTATCAGG
AGATCAAGCAGGCCCTGCTGACAGCACCAGCCCTGGGCCTGCCAGACCTGACCAAGCCTT
TCGAGCTGTTTGTGGATGAGAAGCAGGGCTACGCCAAGGGCGTGCTGACCCAGAAGCTG
GGACCATGGAGACGGCCCGTGGCCTATCTGTCCAAGAAGCTGGACCCAGTGGCAGCAGG
ATGGCCACCATGCCTGAGGATGGTGGCAGCAATCGCCGTGCTGACAAAGGATGCCGGCA
AGCTGACCATGGGACAGCCACTGGTCATCCTGGCACCACACGCAGTGGAGGCCCTGGTG
AAGCAGCCTCCAGATCGCTGGCTGTCTAACGCCCGGATGACACACTACCAGGCCCTGCTG
CTGGACACCGATCGCGTGCAGTTTGGCCCTGTGGTGGCCCTGAATCCAGCCACCCTGCTG
CCTCTGCCAGAGGAGGGCCTGCAGCACAACTGTCTGGACTCTGGCGGCTCAAAAAGAACC
GCCGACGGCAGCGAATTCGAGTCTCCCAAGAAGAAGAGGAAAGTCTAAGTCGACCTTTATT
TGTGAAATTTGTGATGCTATTGCTTTATTTGTAACCATTATAAGCTGCAATAAACAAGTTAAC
AACAACAATTGCATTCATTTTATGTTTCAGGTTCAGGGGGAGATGTGGGAGGTTTTTTAAAG
CAAGCTTGCAAAAAAAGCACCGACTCGGTGCCACTTTTTCAAGTTGATAACGGACTAGCCT
TATTTTAACTTGCTATTTCTAGCTCTAAAAC**CGGCTTTTTCGCGCGCGCGGC**AAACAAGGCT
TTTCTCCAAGGGATATTTATAGTCTCAAAACACACAATTACTTTACAGTTAGGGTGAGTTTCC
TTTTGTGCTGTTTTTTAAAATAATAATTTAGTATTTGTATCTCTTATAGAAATCCAAGCCTATC
ATGTAAAATGTAGCTAGTATTAAAAAGAACAGATTATCTGTCTTTTATCGCACATTAAGCCTC
TATAGTTACTAGGAAATATTATATGCAAATTAACCGGGGCAGGGGAGTAGCCGAGCTTCTC
CCACAAGTCTGTGCGAGGGGGCCGGCGCGGGCCTAGAGATGGCGGCGTCGGATCGCGA
ATTCAAAAAAATTCTAGTTGGTTTAACGCGTAACTAGATAGAACCGCGGTGTGTTTCTGGAG
CTGTTCGGGCTGGCATCTTGCACCGACTCGGTGCCACTTTTTCAAGTTGATAACGGACTAG
CCTTATTTTAACTTGCTATTTCTAGCTCTAAAAC**GCGCGAACAGCTCCAGCCCGC**GGTGTT
TCGTCCTTTCCACAAGATATATAAAGCCAAGAAATCGAAATACTTTCAAGTTACGGTAAGCA
TATGATAGTCCATTTTAAAACATAATTTTAAAACTGCAAACTACCCAAGAAATTATTACTTTCT
ACGTCACGTATTTTGTACTAATATCTTTGTGTTTACAGTCAAATTAATTCTAATTATCTCTCTA
ACAGCCTTGTATCGTATATGCAAATATGAAGGAATCATGGGAAATAGGCCCTCTTCCTGCC
CGACCTTGCGGCCGCAGGAACCCCTAGTGATGGAGTTGGCCACTCCCTCTCTGCGCGCTC
GCTCGCTCACTGAGGCCGGGCGACCAAAGGTCGCCCGACGCCCGGGCTTTGCCCGGGC
GGCCTCAGTGAGCGAGCGAGCGCGCAG

Supplementary References

1. Lapinaite, A., *et al.* DNA capture by a CRISPR-Cas9-guided adenine base editor. *Science* **369**, 566-571 (2020).
2. Huang, T.P., *et al.* Circularly permuted and PAM-modified Cas9 variants broaden the targeting scope of base editors. *Nature Biotechnology* **37**, 626-631 (2019).

3. Oakes, B.L., *et al.* CRISPR-Cas9 Circular Permutants as Programmable Scaffolds for Genome Modification. *Cell* **176**, 254-267.e216 (2019).
4. Zettler, J., Schutz, V. & Mootz, H.D. The naturally split Npu DnaE intein exhibits an extraordinarily high rate in the protein trans-splicing reaction. *FEBS letters* **583**, 909-914 (2009).
5. Levy, J.M., *et al.* Cytosine and adenine base editing of the brain, liver, retina, heart and skeletal muscle of mice via adeno-associated viruses. *Nat Biomed Eng* **4**, 97-110 (2020).
6. Wright, A.V., *et al.* Rational design of a split-Cas9 enzyme complex. *Proceedings of the National Academy of Sciences* **112**, 2984-2989 (2015).
7. Chen, P.J., *et al.* Enhanced prime editing systems by manipulating cellular determinants of editing outcomes. *Cell* **184**, 5635-5652.e5629 (2021).
8. Thomas, D.C., Roberts, J.D. & Kunkel, T.A. Heteroduplex repair in extracts of human HeLa cells. *Journal of Biological Chemistry* **266**, 3744-3751 (1991).
9. Doman, J.L., Sousa, A.A., Randolph, P.B., Chen, P.J. & Liu, D.R. Designing and executing prime editing experiments in mammalian cells. *Nat Protoc* (2022).
10. Liu, P., *et al.* Improved prime editors enable pathogenic allele correction and cancer modelling in adult mice. *Nat Commun* **12**, 2121 (2021).
11. Grünewald, J., *et al.* Engineered CRISPR prime editors with compact, untethered reverse transcriptases. *Nature Biotechnology* (2022).
12. Kotewicz, M.L., Sampson, C.M., D'Alessio, J.M. & Gerard, G.F. Isolation of cloned Moloney murine leukemia virus reverse transcriptase lacking ribonuclease H activity. *Nucleic acids research* **16**, 265-277 (1988).
13. Böck, D., *et al.* In vivo prime editing of a metabolic liver disease in mice. *Science Translational Medicine* **14**, eabl9238 (2022).
14. Zheng, C., *et al.* A flexible split prime editor using truncated reverse transcriptase improves dual-AAV delivery in mouse liver. *Molecular Therapy* **30**, 1343-1351 (2022).
15. Anzalone, A.V., *et al.* Search-and-replace genome editing without double-strand breaks or donor DNA. *Nature* **576**, 149-157 (2019).
16. Nelson, J.W., *et al.* Engineered pegRNAs improve prime editing efficiency. *Nat Biotechnol* **40**, 402-410 (2022).
17. Davidoff, A.M., Ng, C.Y., Zhou, J., Spence, Y. & Nathwani, A.C. Sex significantly influences transduction of murine liver by recombinant adeno-associated viral vectors through an androgen-dependent pathway. *Blood* **102**, 480-488 (2003).
18. Villiger, L., *et al.* Treatment of a metabolic liver disease by in vivo genome base editing in adult mice. *Nat Med* **24**, 1519-1525 (2018).
19. Zaid, A., *et al.* Proprotein convertase subtilisin/kexin type 9 (PCSK9): hepatocyte-specific low-density lipoprotein receptor degradation and critical role in mouse liver regeneration. *Hepatology* **48**, 646-654 (2008).
20. Musunuru, K., *et al.* In vivo CRISPR base editing of PCSK9 durably lowers cholesterol in primates. *Nature* **593**, 429-434 (2021).
21. Rothgangl, T., *et al.* In vivo adenine base editing of PCSK9 in macaques reduces LDL cholesterol levels. *Nature Biotechnology* **39**, 949-957 (2021).
22. Davis, J.R., *et al.* Efficient in vivo base editing via single adeno-associated viruses with size-optimized genomes encoding compact adenine base editors. *Nature Biomedical Engineering* **6**, 1272-1283 (2022).
23. Vozenilek, A.E., *et al.* AAV8-mediated overexpression of mPCSK9 in liver differs between male and female mice. *Atherosclerosis* **278**, 66-72 (2018).
24. Jarrett, K.E., *et al.* Somatic Editing of Ldlr With Adeno-Associated Viral-CRISPR Is an Efficient Tool for Atherosclerosis Research. *Arterioscler Thromb Vasc Biol* **38**, 1997-2006 (2018).

25. Robinet, P., *et al.* Consideration of Sex Differences in Design and Reporting of Experimental Arterial Pathology Studies—Statement From ATVB Council. *Arteriosclerosis, Thrombosis, and Vascular Biology* **38**, 292-303 (2018).
