# Supplementary material for: Efficient prime editing in mouse brain, liver and heart with dual AAVs
Source: Nat Biotechnol. 2023 May 4;42(2):253–64. doi: 10.1038/s41587-023-01758-z (PMC10869272; doi:10.1038/s41587-023-01758-z)
Supplement: Supplementary file 2 — Reporting Summary [file 41587_2023_1758_MOESM2_ESM.pdf]

nature portfolio

Corresponding author(s): David R. Liu

Last updated by author(s): Mar 6, 2023



# Reporting Summary

Nature Portfolio wishes to improve the reproducibility of the work that we publish. This form provides structure for consistency and transparency in reporting. For further information on Nature Portfolio policies, see our Editorial Policies and the Editorial Policy Checklist.

## Statistics

For all statistical analyses, confirm that the following items are present in the figure legend, table legend, main text, or Methods section.

| n/a | Confirmed | |
|---|---|---|
| ☐ | ☒ | The exact sample size (*n*) for each experimental group/condition, given as a discrete number and unit of measurement |
| ☐ | ☒ | A statement on whether measurements were taken from distinct samples or whether the same sample was measured repeatedly |
| ☐ | ☒ | The statistical test(s) used AND whether they are one- or two-sided<br>*Only common tests should be described solely by name; describe more complex techniques in the Methods section.* |
| ☒ | ☐ | A description of all covariates tested |
| ☐ | ☒ | A description of any assumptions or corrections, such as tests of normality and adjustment for multiple comparisons |
| ☐ | ☒ | A full description of the statistical parameters including central tendency (e.g. means) or other basic estimates (e.g. regression coefficient) AND variation (e.g. standard deviation) or associated estimates of uncertainty (e.g. confidence intervals) |
| ☐ | ☒ | For null hypothesis testing, the test statistic (e.g. *F*, *t*, *r*) with confidence intervals, effect sizes, degrees of freedom and *P* value noted<br>*Give P values as exact values whenever suitable.* |
| ☒ | ☐ | For Bayesian analysis, information on the choice of priors and Markov chain Monte Carlo settings |
| ☒ | ☐ | For hierarchical and complex designs, identification of the appropriate level for tests and full reporting of outcomes |
| ☒ | ☐ | Estimates of effect sizes (e.g. Cohen's *d*, Pearson's *r*), indicating how they were calculated |

*Our web collection on statistics for biologists contains articles on many of the points above.*

## Software and code

Policy information about availability of computer code

| | |
|---|---|
| Data collection | Miseq reporter software (v2.6) was used on the Illumina Miseq to demultiplex HTS data. Droplet digital PCR data was collected using QuantaSoft (v.14, Bio-Rad). Histology slides were digitized with a Leica Aperio Slide Scanner. Sony MA900 sorter was used for FACS with MA900 Cell Sorter software v3.1. |
| Data analysis | CRISPResso2 was used to analyze HTS data for quantifying editing activity at genomic sites. Droplet digital PCR data was analyzed using QuantaSoft (v.14-Bio-Rad) |

For manuscripts utilizing custom algorithms or software that are central to the research but not yet described in published literature, software must be made available to editors and reviewers. We strongly encourage code deposition in a community repository (e.g. GitHub). See the Nature Portfolio guidelines for submitting code & software for further information.

## Data

Policy information about availability of data

All manuscripts must include a data availability statement. This statement should provide the following information, where applicable:

- Accession codes, unique identifiers, or web links for publicly available datasets
- A description of any restrictions on data availability
- For clinical datasets or third party data, please ensure that the statement adheres to our policy

High-throughput DNA sequencing data files are available from NCBI SRA under accession code PRJNA898625. DNA sequences of AAV genomes are provided in the Supplementary Information. Key plasmids from this work are available from Addgene and other plasmids and raw data are available from the corresponding author on request.

## Human research participants

Policy information about studies involving human research participants and Sex and Gender in Research.

| | |
|---|---|
| Reporting on sex and gender | The study did not involve human research participants |
| Population characteristics | |
| Recruitment | |
| Ethics oversight | |

Note that full information on the approval of the study protocol must also be provided in the manuscript.

# Field-specific reporting

Please select the one below that is the best fit for your research. If you are not sure, read the appropriate sections before making your selection.

- [x] Life sciences
- [ ] Behavioural & social sciences
- [ ] Ecological, evolutionary & environmental sciences

For a reference copy of the document with all sections, see nature.com/documents/nr-reporting-summary-flat.pdf

# Life sciences study design

All studies must disclose on these points even when the disclosure is negative.

| | |
|---|---|
| Sample size | Sample sizes for tissue culture experiments were chosen in accordance with previous literature and standards in the field of genome-editing technologies (Anzalone 2019, Levy 2020) |
| Data exclusions | No data were excluded from analysis |
| Replication | All replicates reported are independent. All attempts at reproducibility succeeded, as measured by at least two or three positive results |
| Randomization | For tissue culture experiments, conditions were assigned to wells across 96- or 48-well plates and plate positioning is not expected to affect editing efficiency. Mice were assigned to groups randomly and were age-matched between conditions. No covariates were controlled for. |
| Blinding | All HTS data were analyzed by an unblinded operator by using an automated CRISPResso2 script with limited experimenter intervention. We do not anticipate handling to affect editing percentages so did not perform HTS blinded. Plasma analytes were analyzed by a blinded operator, with samples only identifiable through mouse IDs and not treatment. Liver toxicity markers and histology was analyzed by person blinded to treatment conditions. |

# Behavioural & social sciences study design

All studies must disclose on these points even when the disclosure is negative.

| | |
|---|---|
| Study description | *N/A* |
| Research sample | *N/A* |
| Sampling strategy | *N/A* |

| | |
|---|---|
| Data collection | N/A |
| Timing | N/A |
| Data exclusions | N/A |
| Non-participation | N/A |
| Randomization | N/A |

## Ecological, evolutionary & environmental sciences study design

All studies must disclose on these points even when the disclosure is negative.

| | |
|---|---|
| Study description | N/A |
| Research sample | N/A |
| Sampling strategy | N/A |
| Data collection | N/A |
| Timing and spatial scale | N/A |
| Data exclusions | N/A |
| Reproducibility | N/A |
| Randomization | N/A |
| Blinding | N/A |

Did the study involve field work? ☐ Yes ☒ No

## Field work, collection and transport

| | |
|---|---|
| Field conditions | N/A |
| Location | N/A |
| Access & import/export | N/A |
| Disturbance | N/A |

# Reporting for specific materials, systems and methods

We require information from authors about some types of materials, experimental systems and methods used in many studies. Here, indicate whether each material, system or method listed is relevant to your study. If you are not sure if a list item applies to your research, read the appropriate section before selecting a response.

## Materials & experimental systems

| n/a | Involved in the study |
|---|---|
| ☐ | ☒ Antibodies |
| ☐ | ☒ Eukaryotic cell lines |
| ☒ | ☐ Palaeontology and archaeology |
| ☐ | ☒ Animals and other organisms |
| ☒ | ☐ Clinical data |
| ☒ | ☐ Dual use research of concern |

## Methods

| n/a | Involved in the study |
|---|---|
| ☒ | ☐ ChIP-seq |
| ☐ | ☒ Flow cytometry |
| ☒ | ☐ MRI-based neuroimaging |

## Antibodies

Antibodies used: LDLR (Proteintech, 10785-1-AP), β-actin (Cell Signaling Technology, 3700)

Validation: Each antibody was validated by the manufacturers for western blot on cells (LDLR on NIH-3T3 cells, β-actin on C1C12 cells) and for LDLR, tissue (mouse brain) to yield clean bands at the expected molecular weight.

## Eukaryotic cell lines

Policy information about cell lines and Sex and Gender in Research

Cell line source(s): HEK293T, Neuro-2A cells were obtained from ATCC. Mouse astrocytes were a gift from Prof. Bradley Hyman's lab

Authentication: Cell lines were authenticated by the manufacturer and are not commonly misidentified cell lines.

Mycoplasma contamination: All cells initially tested negative for mycoplasma by the manufacturer. Cells were periodically tested during experimentation. No mycoplasma contamination was found.

Commonly misidentified lines (See ICLAC register): No commonly misidentified cell lines were used

## Palaeontology and Archaeology

Specimen provenance: N/A

Specimen deposition:

Dating methods:

☐ Tick this box to confirm that the raw and calibrated dates are available in the paper or in Supplementary Information.

Ethics oversight:

Note that full information on the approval of the study protocol must also be provided in the manuscript.

## Animals and other research organisms

Policy information about studies involving animals; ARRIVE guidelines recommended for reporting animal research, and Sex and Gender in Research

Laboratory animals: Mice were of either the C57BL/6 or APOE4 transgenic C57BL/6 background lines, and were sourced from The Jackson Laboratory, Charles River or Taconic. The mice were P0 to P56 of age during injection.

| | |
|---|---|
| Wild animals | The study did not involve wild animals |
| Reporting on sex | Both male and female mice were used for each condition. |
| Field-collected samples | The study did not involve samples collected from the field. |
| Ethics oversight | The Broad Institute IACUC provided ethical approval for animal experiments (D16-00903; 0048-04-15-2) |

Note that full information on the approval of the study protocol must also be provided in the manuscript.

# Clinical data

Policy information about clinical studies

All manuscripts should comply with the ICMJE guidelines for publication of clinical research and a completed CONSORT checklist must be included with all submissions.

| | |
|---|---|
| Clinical trial registration | N/A |
| Study protocol | N/A |
| Data collection | N/A |
| Outcomes | N/A |

# Dual use research of concern

Policy information about dual use research of concern

## Hazards

Could the accidental, deliberate or reckless misuse of agents or technologies generated in the work, or the application of information presented in the manuscript, pose a threat to:

| No | Yes | |
|---|---|---|
| ☐ | ☐ | Public health |
| ☐ | ☐ | National security |
| ☐ | ☐ | Crops and/or livestock |
| ☐ | ☐ | Ecosystems |
| ☐ | ☐ | Any other significant area |

## Experiments of concern

Does the work involve any of these experiments of concern:

| No | Yes | |
|---|---|---|
| ☐ | ☐ | Demonstrate how to render a vaccine ineffective |
| ☐ | ☐ | Confer resistance to therapeutically useful antibiotics or antiviral agents |
| ☐ | ☐ | Enhance the virulence of a pathogen or render a nonpathogen virulent |
| ☐ | ☐ | Increase transmissibility of a pathogen |
| ☐ | ☐ | Alter the host range of a pathogen |
| ☐ | ☐ | Enable evasion of diagnostic/detection modalities |
| ☐ | ☐ | Enable the weaponization of a biological agent or toxin |
| ☐ | ☐ | Any other potentially harmful combination of experiments and agents |

## ChIP-seq

### Data deposition

☐ Confirm that both raw and final processed data have been deposited in a public database such as GEO.

☐ Confirm that you have deposited or provided access to graph files (e.g. BED files) for the called peaks.

| | |
|---|---|
| Data access links<br>*May remain private before publication.* | N/A |
| Files in database submission | N/A |
| Genome browser session<br>(e.g. UCSC) | N/A |

### Methodology

| | |
|---|---|
| Replicates | |
| Sequencing depth | |
| Antibodies | |
| Peak calling parameters | |
| Data quality | |
| Software | |

## Flow Cytometry

### Plots

Confirm that:

☒ The axis labels state the marker and fluorochrome used (e.g. CD4-FITC).

☒ The axis scales are clearly visible. Include numbers along axes only for bottom left plot of group (a 'group' is an analysis of identical markers).

☒ All plots are contour plots with outliers or pseudocolor plots.

☒ A numerical value for number of cells or percentage (with statistics) is provided.

### Methodology

| | |
|---|---|
| Sample preparation | Preparation of nuclei from tissue for FACS is described in the methods section of this manuscript. |
| Instrument | Sony MA900 Cell Sorter (Sony Biotechnology) |
| Software | SH800S software |
| Cell population abundance | Between 20-60% of sorted cells were GFP-positive |
| Gating strategy | Singlet DyeCycle Ruby-positive events were collected and back-gated to establish FSC/SSC gates. GFP-positive events were clearly separated from GFP-negative events; every experiment used a GFP-negative brain control to define populations. The gating strategy is presented in SI figure 17 |

☒ Tick this box to confirm that a figure exemplifying the gating strategy is provided in the Supplementary Information.

## Magnetic resonance imaging

### Experimental design

| | |
|---|---|
| Design type | N/A |

Design specifications

N/A

Behavioral performance measures

## Acquisition

Imaging type(s)

Field strength

Sequence & imaging parameters

Area of acquisition

Diffusion MRI ☐ Used

## Preprocessing

Preprocessing software

Normalization

Normalization template

Noise and artifact removal

Volume censoring

## Statistical modeling & inference

Model type and settings

Effect(s) tested

Specify type of analysis: ☐ Whole brain ☐ ROI-based ☐ Both

Statistic type for inference
(See Eklund et al. 2016)

Correction

## Models & analysis

| n/a | Involved in the study |
| --- | --- |
| ☐ | ☐ Functional and/or effective connectivity |
| ☐ | ☐ Graph analysis |
| ☐ | ☐ Multivariate modeling or predictive analysis |

Functional and/or effective connectivity

Graph analysis

Multivariate modeling and predictive analysis
